# Targeting immune checkpoint LAIR1 with antibody blockade or 3-in-1 CAR T cells enhances antitumor response

**DOI:** 10.1172/JCI184043

**Published:** 2025-07-01

**Authors:** Haipeng Tao, Dongjiang Chen, Changlin Yang, Duy T. Nguyen, Georges Abboud, Ruixuan Liu, Tianyi Liu, Avirup Chakraborty, Alicia Y. Hou, Nicole A. Petit, Muhammad Abbas, Robert W. Davis, Janie Zhang, Christina Von Roemeling, Mohammed O. Gbadamosi, Linchun Jin, Tongjun Gu, Tuo Lin, Pengchen Wang, Alfonso Pepe, Diego Ivan Pedro, Hector R. Mendez-Gomez, Chao Xie, Aida Karachi, Frances Weidert, Dan Jin, Chenggang Wang, Kaytora Long-James, Elizabeth K. Molchan, Paul Castillo, John A. Ligon, Ashley P. Ghiaseddin, Elias J. Sayour, Maryam Rahman, Loic P. Deleyrolle, Betty Y.S. Kim, Duane A. Mitchell, W. Gregory Sawyer, Jianping Huang

**Affiliations:** 1 Lillian S. Wells Department of Neurosurgery and; 2 The Department of Mechanical and Aerospace Engineering, University of Florida Health Cancer Center, Gainesville, Florida, USA.; 3 Department of Pediatrics, Division of Pediatric Hematology Oncology, and; 4 Preston A. Wells, Jr. Center for Brain Tumor Therapy, University of Florida, Gainesville, Florida, USA.; 5 Division of Quantitative Sciences, Department of Biostatistics and; 6 Bioinformatics, Interdisciplinary Center for Biotechnology Research, University of Florida Health Cancer Center, Gainesville, Florida, USA.; 7 Department of Neurosurgery, The University of Texas MD Anderson Cancer Center, Houston, Texas, USA.

**Keywords:** Oncology, Therapeutics, Cancer immunotherapy, Macrophages, T cells

## Abstract

Tumor-associated macrophages (TAMs) are abundant in the tumor microenvironment (TME) and dampen the immune response, negatively affecting patient survival. Therefore, targeting TAMs could address the limitations of current cancer treatments. However, drug development in this area remains limited. The leukocyte-associated Ig-like receptor 1 (LAIR1), also called CD305, is prominently expressed on the surface of TAMs. We have uncovered what we believe to be a previously unrecognized immunosuppressive LAIR1/factor XIII A/collagen IV pathway across various cancer types. Inhibition of LAIR1, either through knockout (*Lair1*^–/–^), antibody blockade (anti-Lair1 antibody), or a chimeric antigen receptor (CAR) design (3-in-1 CAR by combining tumor targeting, T cell trafficking, and remodeling of the immunosuppressive TME in 1 CAR construct) provided an enhanced antitumor response. LAIR1 inhibition enhanced peripheral and intratumoral CD8 memory T cell populations, induced a phenotypic shift of M2-like macrophages toward M1 macrophages, and normalized tumor collagen IV and structural components in the TME, facilitating effective tumor–T cell interactions and tumor suppression. Enhanced antitumor responses were observed when *Lair1*^–/–^ or anti-Lair1 antibody was used alone or in combination with CAR T cells or when the 3-in-1 CAR T cells were used solely in tumor models resistant to chemotherapy–radiation–programmed cell death protein 1 (PD-1) blockade. These findings position LAIR1 inhibition as a promising strategy for cancer immunotherapies.

## Abstract 2

We uncovered a tumor immunosuppressive pathway involving LAIR1 and developed therapeutic approaches, including antibody blockade and a new signal-delivering CAR to reverse its effect.

## Graphical abstract

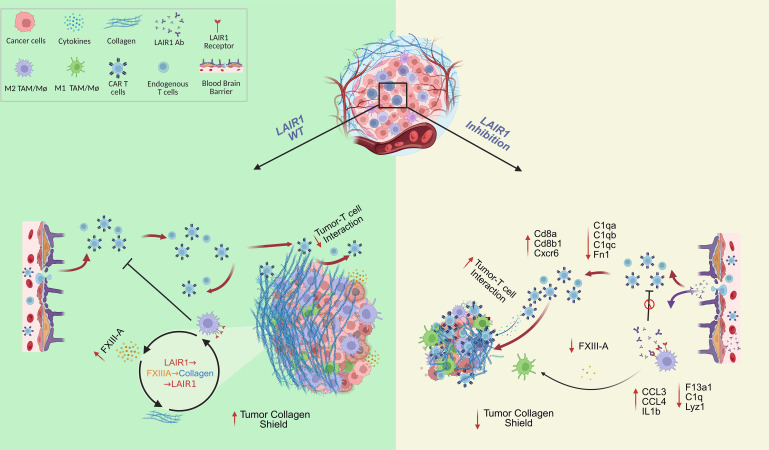

## Introduction

Treating patients with cancer poses significant challenges due to the highly variable clinical behavior and invasive nature of the tumor cells, resulting in failures of standard therapies (1). The presence of residual tumors after treatment often leads to disease recurrence and malignant progression. Cancer immunotherapy has emerged as a promising avenue in the field of precision medicine to address these challenges (2, 3). The widespread applicability of effective immunotherapies for most cancers faces many hurdles, including maintaining effectively engaged tumor-specific T cells within the tumor microenvironment (TME) and preventing them from becoming unresponsive. The remarkable antitumor response elicited by immune checkpoint inhibitors, such as programmed cell death protein 1 (PD-1) blockade, against metastatic cancers of different histologies has generated an unprecedented wave of interest in identifying factors that liberate tumor-specific T cells to exert their effector functions against tumor cells (4–6). These immunotherapeutic antibodies have realized advantages in patient care, including the relatively easy-to-use (off-the-shelf), broad applicability across cancer types and a durable clinical response when treatment is effective (4, 5, 7). However, even the cancer types most responsive to immune checkpoint blockade therapies still result in a significant proportion of refractory patients, suggesting that other inhibitory factors and resistance mechanisms are operative within these tumor-bearing hosts. Emerging evidence indicates that tumor-associated myeloid cells (TAMCs), mainly represented by tumor-associated macrophages (TAMs), predominate the immune TME and may comprise up to 50% of total tumor mass (8–10). TAMs are often considered M2-like macrophages (MΦ) but are specifically tied to the TME (11). To precisely define MΦ within the context of cancer, we use the term “M2-like MΦ” to describe MΦ exhibiting M2-like polarization characteristics generated in vitro, whereas “M2-like TAMs” refers specifically to MΦ exhibiting these characteristics detected inside tumors in this study. These cells are critical in mediating treatment resistance in tumors and affecting cancer progression by interacting directly with tumor cells and/or indirectly enabling a tumor stroma, ultimately reducing patients’ survival (12). Thus, understanding the mechanisms that control the functional activity of M2-MΦ is critical for overcoming the hurdles of treatment and developing therapeutic opportunities to improve the efficacy of cancer therapy.

We have identified leukocyte-associated Ig-like receptor 1 (LAIR1), also known as CD305, as a crucial functional modulator of M2-like MΦ. LAIR1 is a type I transmembrane protein comprising an extracellular Ig domain, a transmembrane region, and a cytoplasmic tail (13). The extracellular domain interacts with its ligands, which include collagens and fibrillar collagens (14). Collagen is a major component of the tumor extracellular matrix (ECM), which plays a significant role in tumor progression by influencing the migration of metastatic cells and providing structural support. Studies have indicated that tumoral collagens serve as functional ligands for LAIR1 (15). LAIR1 is an inhibitory receptor that binds to collagen and contains immunoreceptor tyrosine–based inhibition motifs (ITIMs) (16). It is predominantly expressed on myeloid lineage cells (13, 17–19), particularly M2-like MΦ, which are abundantly presented in cancers and play a central role in tumor progression (20, 21). Previous reports and our results in this study associate LAIR1 expression on M2-like MΦ with clinical outcomes (22), suggesting LAIR1 is a vital molecule that influences the functionality of M2-like MΦ. As a result, there has been a growing interest over the past 5 years among scientists in investigating the role of LAIR1 in cancer progression and its potential as a target for therapeutic drug development (15, 22–26). Nevertheless, our current knowledge regarding the roles of distinct myeloid cell subsets in tumor progression and the effect of LAIR1 on M2-like MΦ functions and response to therapeutic drugs remains limited. Future research is necessary to understand how LAIR1 contributes to M2-like MΦ-mediated immunosuppression/tumor progression and to develop innovative therapeutic strategies targeting LAIR1 signaling.

Our study provides evidence that elucidates the previously uncharacterized LAIR1 signaling pathway in cancer immunosuppression and highlights the role of LAIR1 in enhancing antitumor effectiveness in models. The findings suggest that LAIR1 inhibition, either through antibody blockade or a CAR design, could serve as a standalone treatment or be combined with other therapeutic approaches for effective cancer treatments.

## Results

### LAIR1 is mainly expressed on M2-like MΦ.

**Figure 1:**
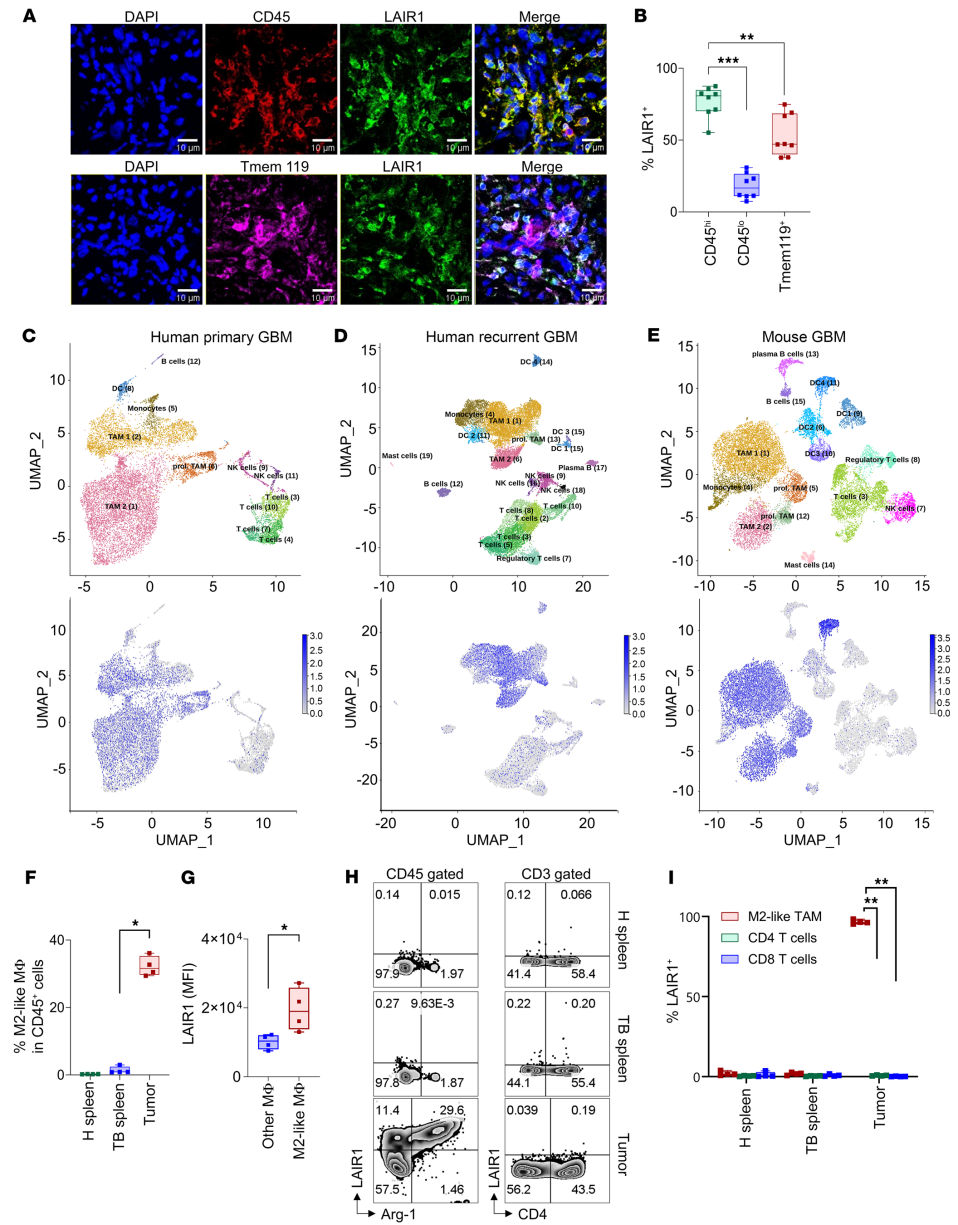
LAIR1 is mainly on CD45^hi^ myeloid cells in human GBM. (**A**) IF images of tumors from a patient with GBM. Scale bars: 10 μm. (**B**) Summary of the percentage of LAIR1^+^ cells on CD45^hi^, CD45^lo^, and Tmem119^+^ cells across 8 human GBMs. The numbers of LAIR1^+^, CD45^+^, and Tmem119^+^ cells were assessed individually. CD45^+^ cells were divided into CD45^hi^ (top 50%) and CD45^lo^ (bottom 50%) populations. The counts of CD45^hi^LAIR1^+^, CD45^lo^LAIR1^+^, and Tmem119^+^LAIR1^+^ cells were determined. (**C**) Uniform manifold approximation and projection (UMAP) presents scRNA-Seq analysis of CD45^+^ cells from 7 human primary GBMs. (**D** and **E**) Results for 4 recurrent human GBMs and 3 GL261 GBMs were obtained using the same approach. The upper panels represent the cell subset maps, and the lower panels show enriched cell populations expressing LAIR1 (blue dots). (**F**) Four C57BL/6 female mice were i.c. injected with cells from the KR158B-Luc GBM line (1 × 10^4^ cells/mouse). Splenic (TB spleen) and tumor tissues were collected on day 35 following tumor implantation. Spleens obtained from healthy (H) age- and sex-matched mice (H spleens, *n* = 4) were used as a control. The samples were evaluated by flow cytometry (FC) to determine the percentage of M2-like TAMs among total CD45^+^ cells, with gating on CD45^+^CD11b^+^F4/80^+^Arg-1^+^ cells. (**G**) M2-like TAMs expressed relatively higher LAIR1 levels than did other MΦ in tumors (all non-M2-like TAMs). (**H** and **I**) High levels of LAIR1 were detected on M2-like TAMs in tumors, whereas minimal-to-no LAIR1 was detected on CD4^+^ or CD8^+^ T cells. The samples in **F** were analyzed by FC for LAIR1 expression in **G**–**I**. The experiments shown in **F**–**I** were repeated at least twice. Data in **B**, **F**, **G**, and **I** are shown as box-and-whisker plots. Statistical significance in **B**, **F**, **G**, and **I** was assessed by Mann-Whitney *U* test. FDR correction was applied in **B**, **F**, and **I**). *P < 0.05, **P < 0.01, and ***P < 0.001.

We found that in primary glioblastoma multiforme (GBM), LAIR1 is positively associated with other immune checkpoints, such as PDCD1 (aka PD-1), CD274 (aka PD-L1), PDCD1LG2 (aka PD-L2), CTLA4, and TIGIT in primary GBM using The Cancer Genome Atlas (TCGA) dataset (Supplemental Figure 1A; supplemental material available online with this article; https://doi.org/10.1172/JCI184043DS1). Furthermore, LAIR1^+^ cells were prominently detected in all 5 GBM specimens by immunofluorescence (IF), while the 5 normal brain control samples exhibited little to no LAIR1 expression (Supplemental Figure 1B). LAIR1^+^ cells extended beyond GBM and were observed in various cancer tissues. According to data from the Human Protein Atlas, these LAIR1^+^ cells were predominantly noncancerous, except for lymphoma, which consisted of malignant immune cells (Supplemental Figure 1C) (28). Furthermore, IF staining of human GBM tissues revealed that LAIR1^+^ cells were predominantly CD45^+^, with no significant overlap with EGFR-expressing tumor cells (Supplemental Figure 1D), a marker in most GBMs (29, 30). Interestingly, we found that the expression pattern of LAIR1 on normal tissues was quite similar to that of PD-L1 (Supplemental Figure 2, A and B) (31). Next, we performed IF staining of GBM specimens, attempting to determine if LAIR1 was on tumor-infiltrating MΦ or resident microglia using CD45 and transmembrane protein 119 (Tmem119), a microglia marker (32). Our findings revealed that most LAIR1^+^ cells (80.9%) were CD45^hi^, whereas a smaller fraction (16.6%) of these cells were CD45^lo^. However, the results regarding Tmem119 were less definitive, as 46.6% of LAIR1^+^ cells were Tmem119^+^, indicating a notable, yet not exclusive, overlap between LAIR1 and Tmem119 expression (Figure 1, A and B). Overall, the data suggest that LAIR1 was mainly expressed on CD45^hi^ cells, and there were some LAIR1^+^Tmem119^+^ cells that were potentially microglia. We then delved into the specific cell type exhibiting LAIR1 expression within the tumor by using single-cell RNA-Seq (scRNA-Seq) data acquired from the Brain Immune Atlas (https://www.brainimmuneatlas.org/) (33). We found that LAIR1 was overexpressed on TAMs on human primary and recurrent GBM samples (Figure 1, C and D) as well as in murine GBM (Figure 1E). We confirmed that a higher proportion of M2-like TAMs were present in the tumors of tumor-bearing (TB) mice (Figure 1F). Furthermore, LAIR1 expression was significantly higher on M2-like TAMs (CD45^+^CD11b^+^F4/80^+^Arg-1^+^ cells) compared with other MΦ (e.g., non-M2-like MΦ, CD45^+^CD11b^+^F4/80^+^Arg-1^–^ cells) (Figure 1G). In contrast, tumor-infiltrating T cells showed minimal or no expression of LAIR1 (Figure 1, H and I). Collectively, these findings suggest that LAIR1 was not predominantly expressed on tumor cells but rather on M2-like TAMs. Together, the gene and protein expression profiles of LAIR1 lay the groundwork for further investigation of this molecule.

### LAIR1-knockout (Lair1^–/–^) mice show enhanced immunity against tumor.

**Figure 2:**
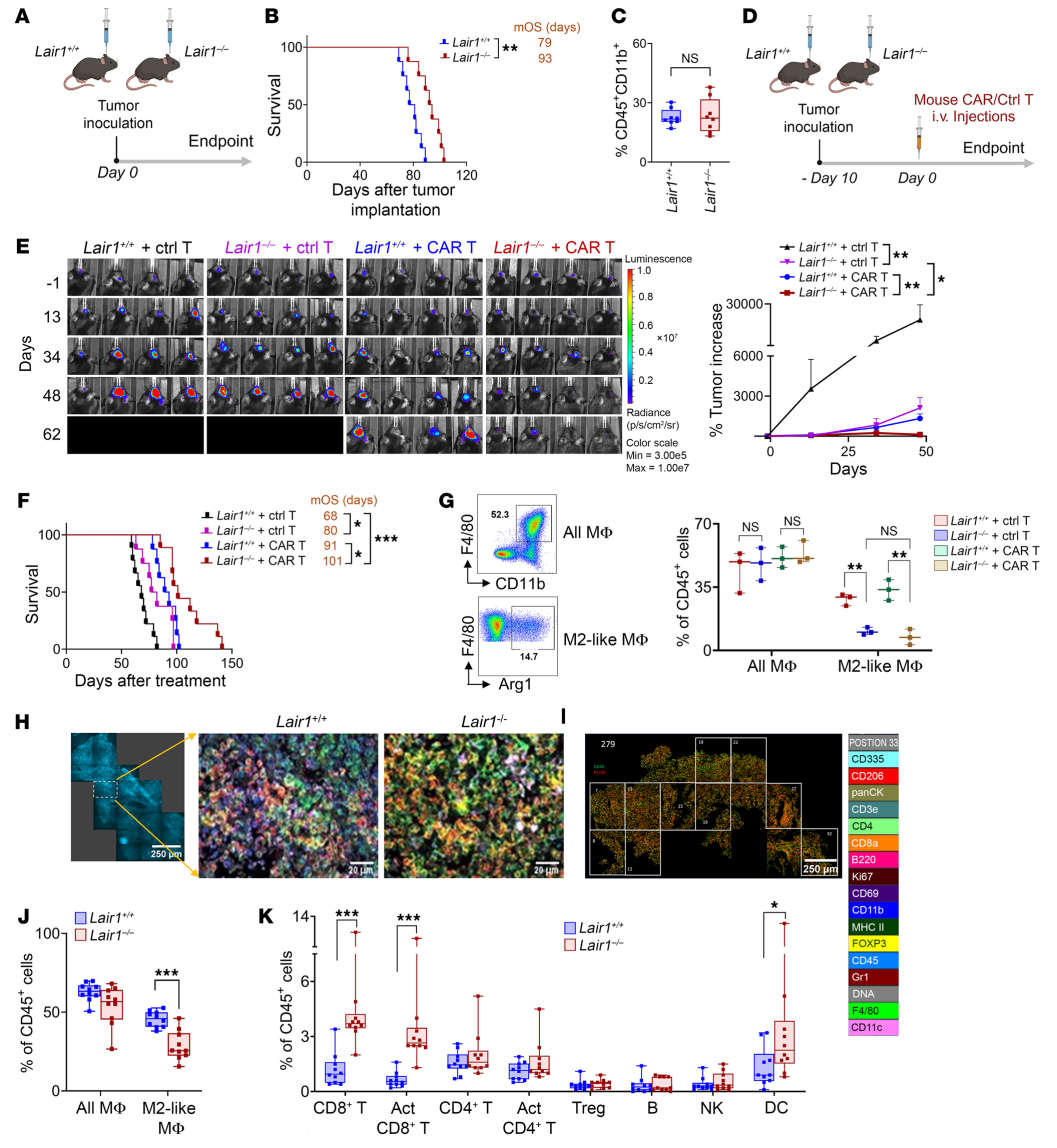
*Lair1* knockout enhances antitumor immunity. (**A**) Experimental design. Female and male LAIR1 WT (*Lair1^+/+^*) and knockout (*Lair1^–/–^*) C57/B6 mice were respectively implanted i.c. with KR158B-CD70-Luc murine glioma cells (1 × 10^4^ cells/mouse, *n* = 8 mice/group) on day 0. (**B**) Survival of the mice was measured. (**C**) Comparison of CD45^+^CD11b^+^ cells in the blood of mice from the 2 groups. Blood was collected from the facial vein 30 days after tumor implantation. (**D**) Experimental design. *Lair1^+/+^* and *Lair1^–/–^* mice were implanted via i.c. with KR158B-CD70-Luc line (1 × 10^4^/mouse, *n* = 8–9/group) 10 days before the treatment (day –10). Mice were treated with either control or mouse CD70CAR T cells (1 × 10^7^/mouse) on day 0 through the tail vein. (**E**) IVIS monitored tumor sizes on the indicated days (left), and a percentage increase was calculated relative to baseline on the day –1 (right). (**F**) Survival was estimated for the groups, with the median overall survival (mOS) shown. (**G**) Evaluation of all-MΦ and M2-like TAMs in tumors. The treatment described in **D** was used. The tumors were collected from mice (*n* = 3 mice/group) 48 days after treatment. (**H**) Comparison of intratumoral immune cells between *Lair1^+/+^* and *Lair1^–/–^* TB mice using ChIP cytometry. Forty days after tumor implantation, tumors were collected from a randomly selected mouse in each group in **A**. Cell types in *Lair1^+/+^* and *Lair1^–/–^* tumors are shown. Scale bars: 250 µm (left) and 20µm (middle and right). (**I**) CD45^+^ cells were analyzed in the 10 ROIs within each tumor. (**J** and **K**) Each cell type was compared between *Lair1^+/+^* and *Lair1^–/–^* TB mice. The in vivo experiments were repeated at least twice. Data are represented as box-and-whiskers plots (**C**, **G**, **J**, and **K**) and the mean ± SEM (**E**). Statistical significance was determined by log-rank test (**B** and **F**), Mann-Whitney *U* test (**C**, **G**, **J**, and **K**), and GEE models (**E**). FDR correction was applied (**E**–**G**). *P < 0.05, **P < 0.01, and ***P < 0.001. Schematics in **A** and **D** were created using BioRender.com.

To explore the role of LAIR1 in tumor progression, we created a *Lair1^–/–^* GBM TB mouse model. We then compared these mice with their WT counterparts (*Lair1^+/+^*) to examine differences in antitumor responses and the immune landscape. Our findings revealed that *Lair1^–/–^* mice survived significantly longer than *Lair1^+/+^* mice (Figure 2, A and B). Additionally, we observed no significant differences in the percentage of CD11b^+^CD45^+^ myeloid cells within PBMCs of animals from either group (Figure 2C). To further explore the potential synergy between *Lair1^–/–^* and CAR T cells in TB mice, we administered murine CD70CAR T cells derived from *Lair1^+/+^* splenocytes to both *Lair1^+/+^* and *Lair1^–/–^* TB mice to target murine CD70^+^ tumors (Supplemental Figure 3A). We observed a synergistic antitumor effect in the *Lair1^–/–^* group combined with mice that underwent CD70CAR T cell treatment, as shown in Figure 2, D–F. Next, we sought to understand how the tumor immune landscape was affected by *Lair1* knockout. We discovered that M2-like TAMs (percentage of CD11b^+^F4/80^+^Arg-1^+^ in CD45^+^ cells) were significantly reduced in *Lair1^–/–^* mice tumors compared with *Lair1^+/+^* mice. However, total MΦ (all-MΦ, percentage of CD11b^+^F4/80^+^ in CD45^+^ cells) remained unchanged. Additionally, our observations revealed that both control and CD70CAR T cell treatments (34) led to a significant decrease in M2-like TAMs in *Lair1^–/–^* TB mice compared with *Lair1^+/+^* TB mice (Figure 2G). To comprehensively understand TME affected by *Lair1^–/–^*, we performed a ChIP cytometric analysis of tumors from *Lair1^+/+^* and *Lair1^–/–^* TB mice using a 16-plex mouse spatial immune cell phenotyping panel (Figure 2H and Supplemental Figures 4 and 5). Tumors were harvested 40 days after implantation, at which point tumor burden in both groups was evident, as determined by our pilot experiment. This analysis confirmed a reduced presence of M2-like TAMs, but not all-MΦ, in tumors from *Lair1^–/–^* mice compared with those from *Lair1^+/+^* mice. Moreover, we found higher quantities of total CD8^+^ T cells, activated CD8^+^ T cells, and DCs in *Lair1^–/–^* tumors than in LAIR*^+/+^* tumors (Figure 2, I–K). Staining for the individual markers CD45 and CD206 was performed (Supplemental Figure 6). Our findings suggest that elimination of LAIR1 contributed to immune landscape changes, e.g., a reduction of M2-like TAMs and an increase in CD8^+^ T cells and DCs, and led to an enhanced antitumor response. The evidence offers promising insights into the therapeutic potential of targeting LAIR1 in cancer treatment.

### anti-Lair1 antibody reverses the M2-like, MΦ-mediated T cell suppression in vitro.

**Figure 3:**
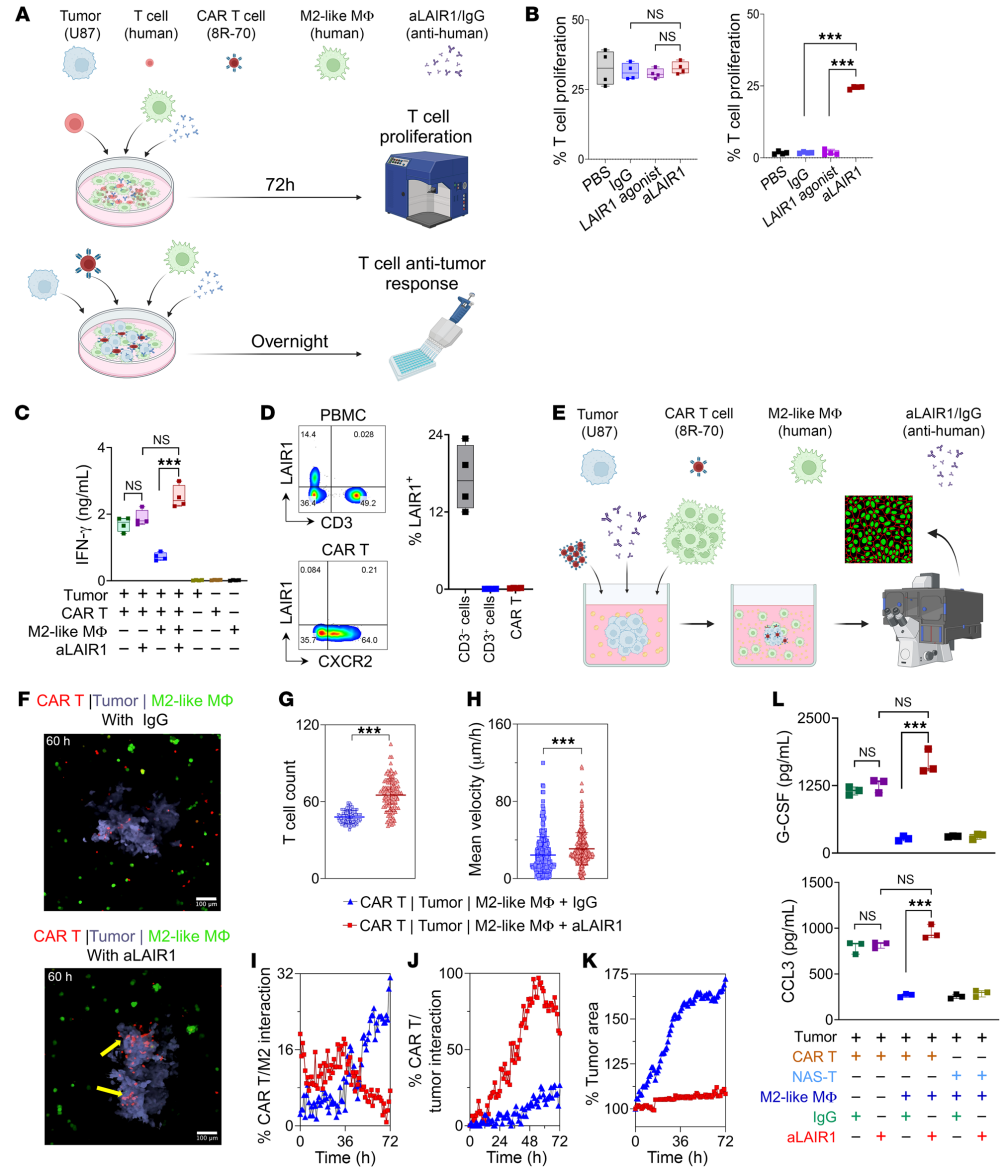
Human aLAIR1 reverses M2-like, MΦ-mediated T cell suppression. (**A**) Experimental design (2D). Human PBMCs were used for autologous 8R-70CAR T cells and the generation of M2-like MΦ. (**B**) CellTrace Violet–labeled T cells were cocultured (*n* = 4/group) without M2-like MΦ (left) or with M2-like MΦ (right) in the presence of PBS/IgG/LAIR1 agonist/anti-LAIR1 antibody (aLAIR1) (5 μg/mL/day). T cell proliferation (CellTrace Violet^–^) was assessed 72 hours after stimulation. (**C**) M2-like MΦ, 8R-70CAR T cells, and CD70^+^ U87 cells were cocultured (ratio: 2 × 10^5^ to 4 × 10^4^ to 1 × 10^5^, respectively) overnight (*n* = 4), and IFN-γ production by CAR T cells was measured. (**D**) PBMCs and CAR T cells derived for coculturing were tested for LAIR1 expression by FC. (**E**) Experimental design (3D). M2-like MΦ, 8R-70CAR T cells, and CD70^+^ U87 cells were cultured (ratio: 8 × 10^3^ to 1.6 × 10^3^ to 4 × 10^3^, respectively) with or without IgG or aLAIR1 (5 μg/mL/day) for 105 hours. (**F**) Video image at 60 hours after coculturing, showing enhanced CAR T cell–tumor interactions in aLAIR1-treated groups (yellow arrows) (see full, live videos in Supplemental Videos 1 and 2). Scale bars: 100 μm. (**G** and **H**) Mean velocity of CAR T cells (0–72 hours) with IgG or aLAIR1. (**I** and **J**) Kinetics of CAR T cell/M2-like MΦ and CAR T cell–tumor interaction in the presence of aLAIR1. The parameter was measured according to the overlapping region of CAR T, M2-like MΦ or according to CAR T/tumor signals as a percentage of total CAR T cells. (**K**) aLAIR1 suppressed tumor growth. Similarly, NAS T cells were analyzed in the presence of IgG or aLAIR1 (Supplemental Figure 8, A–E, and Supplemental Videos 3 and 4). (**L**) Chemokine and cytokine production was influenced by aLAIR1. The culture superannuants were collected 72 hours after the 3D coculture and tested using the Luminex 200. The experiments were repeated at least twice. Data are represented as box-and-whiskers plots (**B**–**D**), the mean ± SD (**G** and **H**), or as points and connecting lines (**H**–**J**). Statistical significance was determined by Mann-Whitney *U* test (**B**, **C**, **G**, **H**, **L**) and GEE models (**I**–**K**, *P* < 0.001). FDR correction was applied (**B** and **C**). ***P < 0.001. Schematics in **A** and **E** were created using BioRender.com.

To evaluate the ability of human anti-Lair1 antibody to affect M2-like, MΦ-mediated immunosuppression, we first assessed anti-Lair1 antibody inhibition of LAIR1-collagen interactions. We evaluated the specific binding of soluble human LAIR1-His protein to collagen using a functional ELISA. The results demonstrated that anti-Lair1 antibody inhibited the binding interaction between LAIR1 and collagen in a dose-dependent manner (Supplemental Figure 7, A–C). Next, autologous human 8R-70CAR T cells and M2-like MΦ were generated from donor PBMC. Both cell types were cocultured with human CD70^+^ GBM (U87) cells (Supplemental Figure 3B) in the presence of PBS, isotype IgG, human LAIR1 agonist, and anti-Lair1 antibody and measured for T proliferation. The results indicated that anti-Lair1 antibody reversed the suppression of T cell proliferation mediated by M2-like MΦ, but did not directly affect the T cells (Figure 3, A and B). Similarly, anti-Lair1 antibody enhanced IFN-γ production by CAR T cells only in the presence of M2-like MΦ; no such effect was observed in their absence (Figure 3, A and C). Additionally, under the experimental conditions used in this study, we detected no LAIR1 expression on CAR T cells (Figure 3D). Next, we leveraged 3D-printing technology to assess the role of LAIR1 in a model that better emulated the intricate physiological conditions and composition of the TME. We visualized the effect of anti-Lair1 antibody on interplay between CAR or non-antigen-specific (NAS) T cells, M2-like MΦ, and human GBM U87 tumor spheres (Figure 3E and Supplemental Videos 1–4). We chose a relatively low effector/target (E/T) ratio at 5:1:2.5 (M2-like MΦ/CAR T/cancer cells), so single cells could be easily tracked and quantified, enabling the detection of cell-cell interactions and CAR T cell infiltration in a more sensitive setting (35). The representative images captured from the videos 60 hours after coculturing showed more CAR T cell–tumor interactions by the anti-Lair1 antibody than by IgG (Figure 3F). Quantitative analysis revealed that anti-Lair1 antibody counteracted the inhibitory effects of M2-like MΦ on CAR T cells, leading to an increased overall number of CAR T cells and enhanced migratory speed (Figure 3, G and H). Additionally, anti-Lair1 antibody reduced CAR T cell–M2-like MΦ interactions after approximately 35 hours (Figure 3I) and promoted increased CAR T cell–tumor interactions (Figure 3J). Notably, this resulted in substantial tumor inhibition, as evidenced by a reduced tumor area (Figure 3K). The effect of the anti-Lair1 antibody on NAS T cells followed a similar trend but to a much lesser extent in terms of T cell–tumor interactions and tumor inhibition, likely due to the lack of antigen specificity (Supplemental Figure 8, A–E, and Supplemental Videos 3 and 4). Furthermore, we observed that cytokine and chemokine secretions that help proper T cell recognition (granulocyte-CSF [G-CSF]) (36) (Figure 3L) and intratumoral trafficking (CCL3) (37) were elevated by anti-Lair1 antibody in the culture supernatants at 72 hours compared with IgG (Figure 3L). These data show that LAIR1 directly contributed to M2-like, MΦ-mediated suppression of T cells (without directly affecting the T cells themselves) and that this suppression could be reversed through targeted blockade of LAIR1.

### Anti-Lair1 antibody enhances the antitumor response in tumor models.

**Figure 4:**
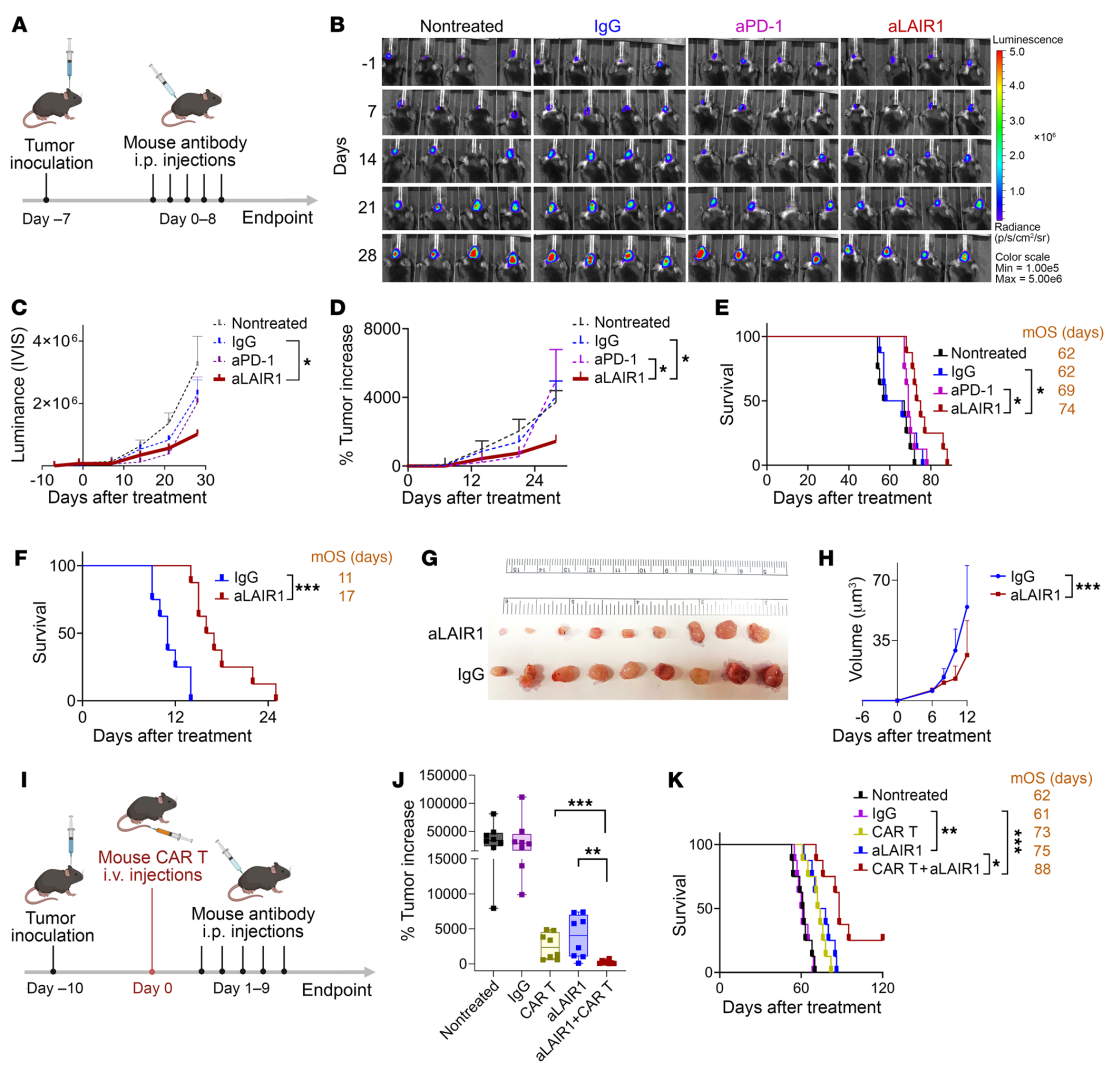
Mouse aLAIR1 enhances antitumor response in tumor models. (**A**) Experimental design for **B**–**H**. The GBM cell lines KR158B-CD70-Luc (1 × 10^4^ cells/mouse, females, *n* = 8/group) and GL-261-Luc (1 × 10^5^ cells/mouse, female, *n* = 8/group) were i.c. implanted. LLC1 (1 × 10^5^ cells/mouse, *n* = 9/group) was s.c. injected into female C57BL/6 mice 7 days before treatment. TB mice received a 5-dose treatment (200 μg/dose/mouse) of aLAIR1, aPD-1, or IgG via i.p. injection every other day from day 0 to day 8. (**B** and **C**) IVIS was performed to monitor tumor size on the indicated days before and after treatment. (**D**) Tumor increase after treatment was compared by IVIS relative to baseline values recorded on day –1. (**E** and **F**) Survival of KR158B-CD70-Luc and GL261-Luc mice after aLAIR1, aPD-1, or IgG treatment. (**G** and **H**) aLAIR1 improved the antitumor response in the LLC1 model. Tumor volume was calculated by the ellipsoidal formula: V = ½ (width^2^ × length) from day –7 to day 12 after aLAIR1 or IgG treatment. Tumors were collected on day 13 after treatment. (**I**) Experimental design for the combination therapy using aLAIR1 with mouse CD70CAR T cells. The KR158B-CD70-Luc cells were i.c. implanted (1 × 10^4^ cells/mouse, *n* = 8/group) into female C57BL/6 mice 10 days before the CAR T cell treatment. Subsequently, 1 × 10^7^ mouse CD70CAR T cells were injected via the tail vein on day 0, following 5 doses of aLAIR1 or IgG (200 μg/dose/mouse) administered to the mice by i.p. injection every other day from days 1–9. (**J**) Tumor increase following the indicated treatment at day 39 after treatment. All experiments were repeated at least twice. Data are represented as the mean ± SEM (**C**, **D**, and **H**) and box-and-whiskers plots (**J**). Statistical significance was determined using GEE models (**C** and **D**), the log-rank test (**E**, **F**, and **K**), GEE models (**H**), and the Mann-Whitney *U* test (**J**). FDR correction was applied (**C**–**E**, **J**, and **K**). *P < 0.05, **P < 0.01, and ***P < 0.001. Schematics in **A** and **I** were created using BioRender.com.

Next, we evaluated the effect of anti-Lair1 antibody on the antitumor response using tumor models. We first tested the antitumor effect of the anti-Lair1 antibody in a PD-1 blockade–resistant GBM model (27). KR158B-CD70-Luc murine GBM was orthotopically implanted into C57BL/6 mice. Isotype IgG, anti-Lair1 antibody, or aPD-1 antibodies (5 doses of 200 μg/dose) were administered to the animals as indicated, and outcomes for the treated animals were measured (Figure 4A). The results demonstrated that anti-Lair1 antibody treatment more effectively inhibited tumor growth than did the IgG control, and it surpassed aPD-1 in tumor shrinkage at later time points (Figure 4, B–D). A notable extension of survival was observed when comparing the untreated, IgG, and anti-Lair1 antibody–treated groups. Crucially, anti-Lair1 antibody treatment had a more pronounced effect than did aPD-1 treatment in the KR158B-CD70-Luc GBM model (Figure 4E). Prolonged survival was also seen in GL261-Luc, another GBM model (38) (Figure 4F). To determine if anti-Lair1 antibody is able to result in tumor regression in other cancer types, a Lewis lung carcinoma (LLC1) syngeneic model was tested. The result revealed a significant antitumor response after anti-Lair1 antibody treatment compared with the IgG control (Figure 4, G and H), suggesting that the antitumor capability of anti-Lair1 antibody extended beyond GBM. Next, we aimed to investigate whether anti-Lair1 antibody treatment could enhance the effectiveness of CAR T cell therapy. We conducted experiments using our first generation of mouse CD70CAR T cells (34). These cells demonstrated only modest efficacy compared with the 8R-70CAR T cells currently being used in a phase I clinical trial for patients with primary GBM (NCT05353530). The unique advantage of 8R-70CAR T cells lies in the incorporation of the IL-8 receptor, which functions as a “GPS signal” within the CAR design. This enhancement significantly improves T cell intratumoral trafficking, thereby substantially enhancing the antitumor efficacy of these CAR T cells (39). Five doses of anti-Lair1 antibody (200 μg/dose) were administered after tumor establishment followed by 5 doses of anti-Lair1 antibody (200ug/dose) and 1 dose of mouse CD70CAR T cells (Figure 4I). The results showed that CAR T cells and anti-Lair1 antibody treatment alone inhibited tumor growth, and a synergistic antitumor effect was seen when anti-Lair1 antibody was combined with the CAR T cells (Figure 4, J and K). The results highlight a promising strategy for optimizing CAR T cell therapy by integrating anti-Lair1 antibody treatment. These findings support our hypothesis that LAIR1 functions as a myeloid checkpoint and that inhibiting its activity enhances antitumor responses, particularly in aPD-1–resistant tumor models.

### Delivery of LAIR1 inhibition by 3-in-1 CAR T cells reshapes the TME and enhances the antitumor response.

**Figure 5:**
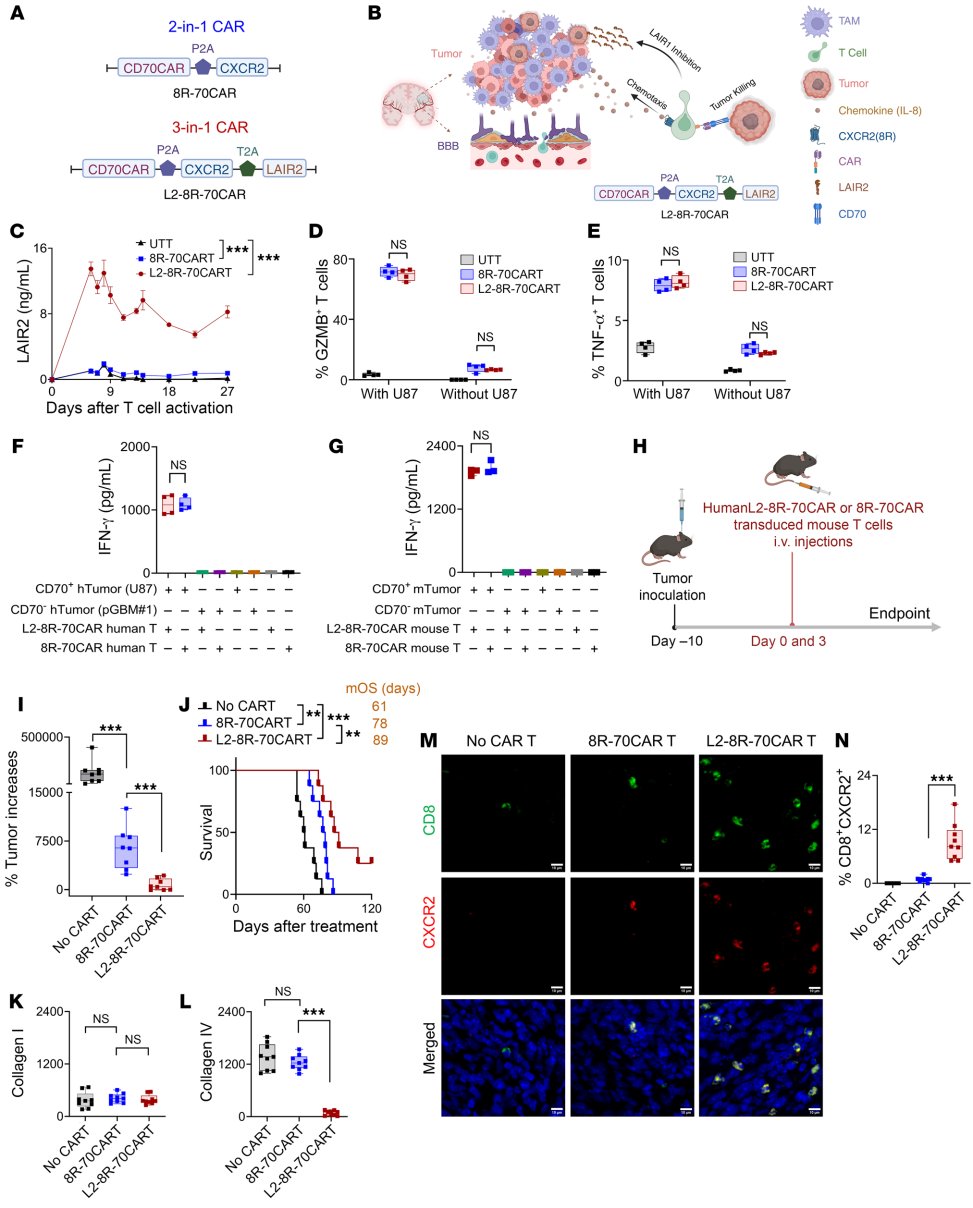
L2-8R-70CAR T cells enhance the antitumor response. (**A**) Diagram of human L2-8R-70CAR and 8R-70CAR constructs. (**B**) Schematic representation of the 3-in-1 functionality of L2-8R-70CAR T cells. (**C**) T cells derived from a donor’s PBMCs were transduced with L2-8R-70CAR T cells (*n* = 3), 8R-70CAR T cells (*n* = 1), or untransduced T cells (UTT) (*n* = 1), respectively. Supernatants from T cell cultures were collected over a 27-day period for LAIR2 secretion by ELISA. (**D** and **E**) GZMB and TNF-α expression in T cells was assessed by FC following coculturing of CAR T cells (1 × 10^5^) with CD70^+^U87 (1 × 10^5^) cells (*n* = 4) overnight. (**F** and **G**) Human (h) and mouse (m) CAR T cells specifically recognized their respective CD70-expressing tumor origins (hTumor: CD70^+^U87 and CD70^–^ pGBM1; mTumor: KR158B-CD70-Luc and KR158B-Luc). (**H**) Experimental design. C57BL/6 splenic T cells were transduced with 8R-70CAR or L2-8R-70CAR T cells. Mice (*n* = 3 groups, *n* = 8 mice/group) received i.c. injections of 1 × 10^4^ CD70^+^ mTumor cells. Two of the groups were i.v. administered 1 × 10^7^ CAR T cells on days 0 and 3, and the third group was untreated. (**I** and **J**) The percentage of tumor increase (day 38 a treatment) and survival were monitored. (**K** and **L**) mTumor sections (day 39 after treatment, **H**) were stained for collagen I and collagen IV. MFI was calculated as follows: (total intensity – background)/nuclei. (**M**) Comparison of intratumoral T cell infiltration after treatment. IF images of intratumoral CAR T cells were visualized, showing mouse CD8, human CXCR2, and DAPI staining in tumor sections. Scale bars: 10 μm. (**N**) Summary of tumor samples from 3 mice (*n* = 3 randomly selected sections per mouse) in each treatment group, showing the percentage of CD8^+^CXCR2^+^ cells relative to total nuclei. All experiments were repeated twice. Data are represented as the mean ± SEM (**C**) and box-and-whiskers plots (**D**–**G**, **I**, and **K**–**M**). Statistical significance was determined using GEE (**C**, **K**, **L**, and **N**), the Mann-Whitney *U* test (**D**–**G**, and **I**), and the log-rank test (**J**). FDR correction was applied for all comparisons. **P < 0.01 and ***P < 0.001. Schematics in **A**, **B**, and **H** were created using BioRender.com.

To address the significant challenges posed by the blood-brain barrier (BBB) (40) and the tumor physical barrier (TPB) in delivering antibody drugs for brain tumors and other cancers, we developed a 3-in-1 CAR construct, the human L2-8R-70CAR. This new CAR design combines targeting of CD70 on tumors (34, 39), enhancement of T cell tumor trafficking via expression of the IL-8 receptor (39), and inhibition of LAIR1 by secreting soluble LAIR2 to remodel the immunosuppressive TME (22, 24, 26) (Figure 5, A and B). This advancement builds upon our previous 8R-70CAR (2-in-1 CAR) construct (39) used in the clinical trial. The L2-8R-70CAR–transduced human T cells secreted a significantly higher and sustained amount of LAIR2 than the 8R-70CAR T cells in vitro (Figure 5C), while maintaining a similar effector function, as measured by granzyme B (GZMB), TNF-α, and IFN-γ, when encountering tumors (Figure 5, D–F). To further evaluate the efficacy of the human L2-8R-70CAR construct in mice, we conducted in vitro experiments using mouse T cells transduced with the human L2-8R-70CAR and 8R-70CAR constructs. The results showed that both CAR T cells equally recognized mouse KR158B-CD70-Luc tumors (Figure 5G). Since the collagen-binding site on LAIR1 is conserved between humans and mice (41), and human LAIR2 has been shown to block murine LAIR1 in previous studies (22, 24), we evaluated the CAR T cells in the immunocompetent KR158B-CD70-Luc mouse model (Figure 5H). The results demonstrated that L2-8R-70 CAR T cells were superior to 8R-70 CAR T cells in terms of the antitumor response in mice (Figure 5, I and J). Furthermore, we found that L2-8R-70 CAR T cells significantly reduced collagen IV but not collagen I levels in the tumors compared with 8R-70CAR T cells and nontreated (no–CAR T) control cells (Figure 5, K and L). Last, we observed a greater number of tumor-infiltrating L2-8R-70 CAR T cells (CD8^+^CXCR2^+^, defined by mouse CD8 and human CXCR2 markers) compared with 8R-70 CAR T cells and the no–CAR T control cells (Figure 5, M and N) (34, 39). In summary, our animal data provide evidence that human 3-in-1 CAR T cells can have multiple functional effects, including tumor targeting, T cell trafficking, and TME remodeling by reducing tumor collagen IV levels. These combined effects can significantly enhance the antitumor response, offering promising therapeutic potential for treating the most challenging cancers.

### LAIR1 inhibition breaks the LAIR1/FXIII-A/collagen immunosuppressive pathway.

**Figure 6:**
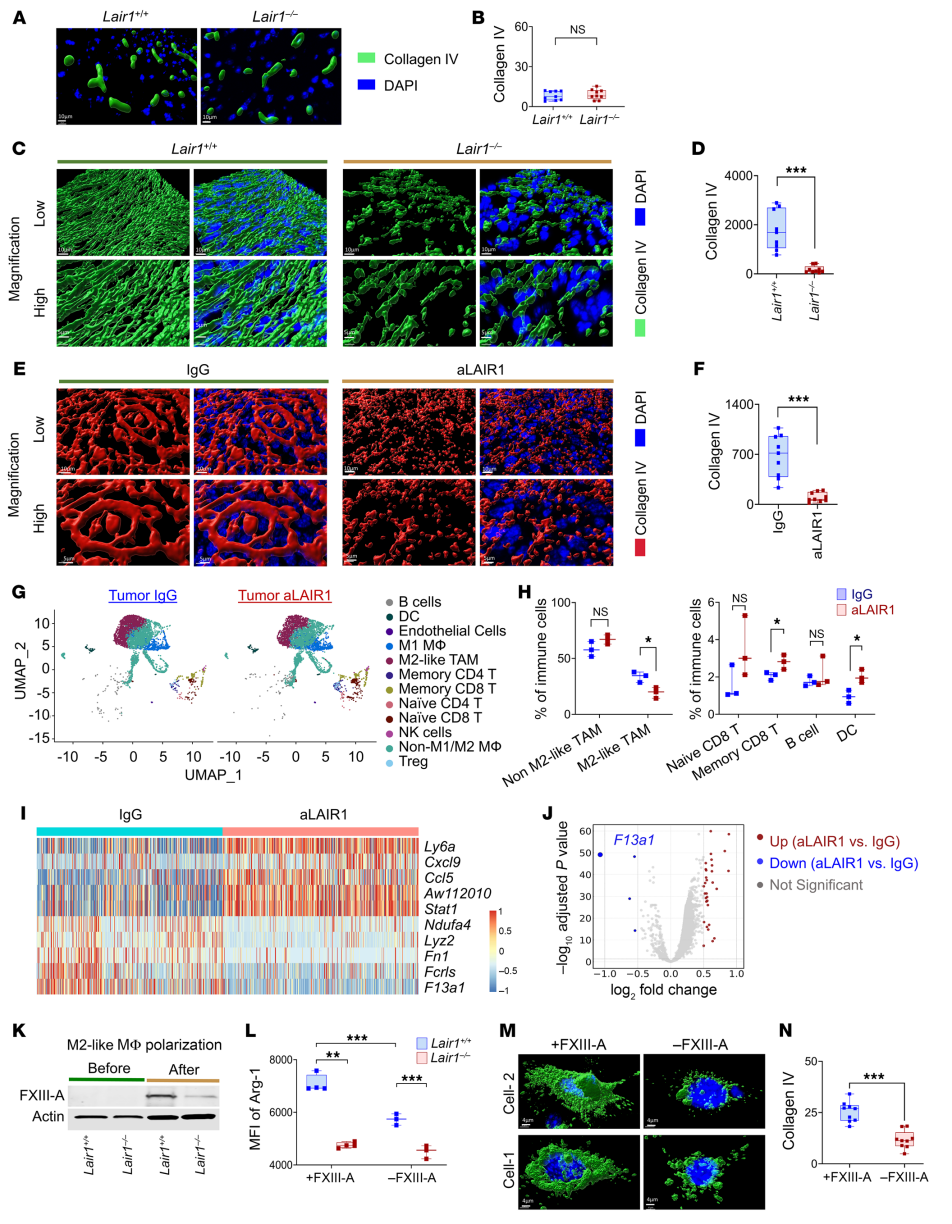
LAIR1 inhibition reduces tumor collagen IV by lowering FXIII-A in M2-like TAMs and MΦ. (**A** and **B**) Collagen IV IF staining was performed on normal brain tissues from *Lair1^+/+^* and *Lair1^–/–^* mice, and MFI was quantified. Scale bars: 10 μm. (**C** and **D**) Tumors from *Lair1^+/+^* and *Lair1^–/–^* TB mice (KR158B-CD70-Luc) were collected 40 days after treatment (Figure 2A) and stained for collagen IV. Scale bars: 10 μm (top), 5 μm (bottom). (**E** and **F**) IF images showing collagen IV staining of tumor tissue from C57BL/6 TB mice (LLC1) treated with IgG or aLAIR1, and tumors were collected on post-treatment day 13 (Figure 4G). Scale bars: 10 μm (top), 5 μm (bottom). The 3D image reconstruction (**A**, **C**, and **E**) and quantification (**B**, **D**, and **F**) were based on 2D images (*n* = 3 mice/group, *n* = 9 sections total) were performed. (**G** and **H**) C57BL/6 TB mice (KR158B-CD70-Luc, 3/group) with or without IgG or aLAIR1 (experimental design as in Figure 4A). Tumor scRNA-Seq was performed 49 days after treatment. UMAPs illustrate immune cell clusters with population summaries (>1%) shown. (**I**) Heatmap of the top 5 up- and downregulated genes in all-MΦ clusters comparing the aLAIR1- versus IgG-treated groups. (**J**) Volcano plot highlighting DEGs in M2-like TAMs, identifying *F13a1*, which encodes FXIII-A. (**K**) BM monocytes from *Lair1^+/+^* and *Lair1^–/–^* C57BL/6 mice were cultured with M-CSF (100 ng/mL, 7 days), followed by IL-4, IL-10, and IL-13 (20 ng/mL, 2 days) to induce M2-like MΦ. Control cells were cultured without cytokines. Cells were harvested on day 9, and FXIII-A expression was assessed by Western blotting. (**L**) Arg-1 MFI was measured by FC in BM-derived M2-like MΦ (*n* = 3~4) treated with or without 10 μg/mL murine FXIII-A for 48 hours. M2-like MΦ were gated on CD45^+^CD11b^+^F4/80^+^Arg-1^+^cells. (**M** and **N**) KR158B-CD70-Luc cells (*n* = 9/group) were incubated with or without 10 μg/mL FXIII-A for 24 hours, and collagen IV levels were quantified and expression imaged. Scale bars: 4 μm. All data were reproduced 2 or more times. Data are shown as box-and-whisker plots (**B**, **D**, **F**, **H**, **L**, and **N**). Statistical significance was determined by GEE (**B**, **D**, and **F**), a 2-sample *t* test (**H** and **J**), and the Mann-Whitney *U* test (**L**). FDR correction was applied (**J**). *P < 0.05, **P < 0.01 and ***P < 0.001.

We observed a notable reduction in collagen IV levels in mouse GBM following L2-8R-70CAR T cell treatment. To further investigate whether LAIR1 inhibition influences tumor collagen deposition, normal brain and tumor tissues were collected from *Lair1^+/+^* and *Lair1^–/–^* mice and stained with collagen I and IV, respectively. The results showed that *Lair1^–/–^* did not affect collagen IV deposition in normal brain tissues (Figure 6, A and B). Differential collagen levels were initially assessed using 2D imaging, demonstrating the distribution of collagen and nuclei (Supplemental Figure 9). Remarkably, through 3D imaging reconstruction, we visualized increased collagen IV accumulation in *Lair1^+/+^* tumors, characterized by a dense “collagen web” structure that enveloped tumor cells and obscured tumor nuclei. In contrast, *Lair1^–/–^* tumors showed significantly reduced collagen deposition and clearly exposed tumor nuclei (Figure 6, C and D), more closely resembling normal brain tissue. In GBM-bearing mice treated with anti-Lair1 antibody, we observed a moderate reduction in collagen deposition compared with *Lair1^–/–^* mice, which could have been due to the delayed timing of sample collection (63 days after the final dose of anti-Lair1 antibody) (Supplemental Figure 10, A and B). Importantly, we consistently observed a more significant change in collagen IV expression levels and structure in anti-Lair1 antibody–treated LLC1 tumors (Figure 6, E and F), reproducing the findings from the GBM model in *Lair1^–/–^* mice. We observed no changes in collagen I levels in these tumors (Supplemental Figure 11). To investigate the mechanism of action of LAIR1 in M2-like MΦ, we performed scRNA-Seq analysis (the cell population categorization used in the analysis is shown in Supplemental Figure 12) using tumor samples from total 6 KR158B-CD70-Luc TB mice treated with IgG or anti-Lair1 antibody, which showed a trend of antitumor efficacy (Supplemental Figure 13A). The results demonstrated that anti-Lair1 antibody significantly increased naive CD8^+^ T cells in spleens (Supplemental Figure 13, B and C) and reduced M2-like TAMs and increased memory CD8^+^ T cells and DCs in tumors (Figure 6, G and H). The reduction in the presence of M2-like TAMs within tumors (at the cellular level) by anti-Lair1 antibody was correlated with increased survival. In contrast, no such association was observed in IgG-treated mice (Supplemental Figure 13, D and E), supporting our hypothesis that LAIR1 inhibition in M2-like TAMs enhances the antitumor response. In light of the observed differential antitumor responses between treatments with anti-Lair1 antibody and aPD-1 using the KR158B-Luc model, we aimed to analyze the tumors to pinpoint the unique attributes of each treatment. Our findings revealed that anti-Lair1 antibody led to reduced M2-like TAM frequencies (21.4% vs. 38.7%) and higher frequencies of naive CD8^+^ T cells (2.5% vs. 0.5%) and memory CD8^+^ T cells (2.5% vs. 1.4%) than aPD-1 treatment (Supplemental Figure 13F). However, given the limited sample size (3 samples/group pooled for scRNA-Seq analysis) in this study, further research is necessary to draw definitive conclusions. Additionally, our findings indicated that anti-Lair1 antibody did not directly affect the viability or phenotype of LAIR1^+^ myeloid cells (Supplemental Figure 14, A–C). Further analysis focused on identifying the top modulated genes in all MΦ affected by anti-Lair1 antibody treatment using data from Figure 6G. The results revealed a reduction in signature genes indicative of M2-like TAMs, such as *F13a1* (42), and *Fn1* (43). Conversely, we noted an increase in the expression of genes, e.g., *Cxcl9* (44) and *Stat1* (45*)*, associated with M1-MΦ following the treatment (Figure 6I). We observed a significant decrease in expression of the *F13a1* gene, which encodes the protein factor XIII-A (FXIII-A), following LAIR1 inhibition (Figure 6J). Notably, *F13a1* expression positively correlated with LAIR1 expression at the gene level, as demonstrated using primary GBM data from TCGA RNA-Seq database (Supplemental Figure 15A). Interestingly, we observed greater *F13a1* and *LAIR1* expression overlap in recurrent tumors compared with expression levels in primary tumors (Supplemental Figure 15B) when evaluating the same population of LAIR1^+^ M2-like TAMs shown in Figure 1, C and D. This discrepancy presents an intriguing puzzle for further investigation. Confirming this observation regarding the association between LAIR1 and FXIII-A, the protein expression of FXIII-A was reduced in M2-like MΦ derived from *Lair1*^–/–^ hosts compared with those from *Lair1^+/+^* counterparts (Figure 6K). The addition of FXIII-A protein to bone marrow–derived (BM-derived) monocytes during M2-like MΦ generation significantly elevated Arg-1 levels, a key murine M2-like MΦ marker, only in the *Lair1^+/+^* mice and not in the *Lair1^–/–^* mice (Figure 6L). These results suggest that LAIR1 is critical for FXIII-A–mediated M2-like MΦ generation and imply that FXIII-A may act as a downstream component of the LAIR1 signaling pathway. To investigate this further, we examined whether FXIII-A directly influences tumor collagen formation and deposition. The data demonstrated that treating murine KR158B-CD70-Luc GBM cells with FXIII-A in vitro for 24 hours significantly increased collagen IV density compared with untreated cells (Figure 6, M and N). These findings indicate that FXIII-A plays a role in tumor collagen IV deposition and structural remodeling. Summarizing our findings, we have demonstrated a mechanism involving LAIR1^+^ M2-like MΦ/TAMs in tumor immunosuppression and progression. We show that these MΦ secreted FXIII-A in the surrounding matrix, leading to collagen formation and deposition remodeling. This effect triggered LAIR1^+^ M2-like MΦ generation and polarization, stimulating the cells to secrete more FXIII-A, thereby creating a continuous loop that supported M2-like, TAM-mediated tumor immunosuppression in the TME. Breaking this pathway by LAIR1 inhibition was demonstrated in this study to reverse the immunosuppression mediated by M2-like TAMs, enhance T cell functions, and induce a significant antitumor response.

## Discussion

The relevance of TAMs in cancer progression and therapy has spurred interest in therapeutically targeting these cells. However, we still have an incomplete and potentially limited understanding of how the various myeloid cell subtypes function in vivo during tumor progression and how drugs alter the activity of these cells (46). Identification of the new TAMC immune checkpoint, LAIR1, in patients with cancer and tumor-burdened mice provides an opportunity to determine a related pathway that links with TAM-induced tumor immunosuppression and helps to enhance endogenous multiplex immunity and adoptive cell therapy, such as CAR T cell therapy, against cancer. Our journey began with a study that focused on the role of an immunosuppressive ligand, CD70, in GBM progression (34, 39, 47). Recent studies have evaluated the biological role of LAIR1 in solid tumors (15, 22–26), and more studies are proposed to explore and define the precise mechanisms underlying the functions of LAIR-1 in tumor immunology and biology.

There are 2 strategies to inhibit LAIR1 signaling: one is by eliminating the ligand (collagen) effect, and the other is by blocking the receptor (LAIR1) itself on LAIR1-expressing cells. Most recent studies have involved strategies to inhibit LAIR1 signaling through collagen inhibition using LAIR2. LAIR2 is a homolog of LAIR1, a naturally secreted soluble protein that acts as a decoy receptor by binding collagen with a higher affinity than is seen with LAIR1 (48), which has been shown to block the action of LAIR1-collagen interaction and improve the antitumor response (22, 24, 26). An early-phase clinical trial has been conducted using the LAIR2-Fc fusion protein or its combination with other drugs for patients with cancer (26, 49, 50). However, given collagen’s ubiquity, targeting collagens for cancer therapies poses several problems, including potential side effects in nontarget tissues. The heterogeneity of collagens makes specific targeting difficult, since the varied collagen types might compensate for targeted loss, which could lead to therapy resistance. Collagen’s location in the tumor ECM can also hinder precise therapeutic delivery. Additionally, the role of collagen in normal physiological processes could mean that targeting it may disrupt essential functions. Thus, inhibiting LAIR1 activity using LAIR1 antibody blockade (anti-Lair1 antibody), which mainly affects M2-like MΦ and TAMs in peripheral blood and tumors, or using CAR T cells that specifically deliver the LAIR1 inhibition within the TME could offer more effective and targeted approaches.

In this study, we primarily used GBM models because GBM represents one of the most complex and resistant cancers. GBMs is characterized by significant intratumoral heterogeneity, rapid progression, and treatment resistance (51–53). Immunologically, GBM is marked by a low presence of T cells and a high density of M2-like TAMs within the tumor (8, 9, 54). The limited T cell infiltration and low PD-L1 expression on tumor cells complicate the efficacy of PD-1 blockade (55), highlighting the need for more effective immune checkpoint blockades. Additionally, the BBB significantly hinders the delivery of therapeutic antibodies to the tumor. However, the BBB’s properties change during tumor growth and inflammation, becoming progressively disrupted in these contexts (56, 57), which helps open the BBB and facilitates the delivery of anti-Lair1 antibody. Notably, the innovation of using 3-in-1 CAR T cells to deliver soluble LAIR2 for LAIR1 inhibition enables more effective treatment of GBM. The peripheral effect of anti-Lair1 antibody on enhancing tumor immunity proves crucial for the observed in vivo antitumor response. Thus, GBM is a benchmark for cancer complexity, and overcoming its challenges could provide insights for treating other cancers.

The data of the *Lair1^–/–^* experiments established a solid foundation to untangle the role of LAIR1 in tumor immunosuppression and devise potential therapeutic strategies. We observed that *Lair1^–/–^* tumor-bearing mice had a more robust antitumor response than did their *Lair1^+/+^* counterparts. Significantly, we also observed this augmented antitumor response upon administration of CAR T cell therapy. This suggests that *Lair1^–/–^* creates a more favorable TME for the function of CAR T cells, leading to a synergistic effect. Because MΦ, a cell type typically dominant in the tumor, can be classified into M1 and M2 phenotypes with distinct functions. M1-MΦ are reported to be proinflammatory and antitumorigenic, while M2-like MΦ, often presented as TAMs or M2-like MΦ, are antiinflammatory and protumorigenic (58, 59). We found that LAIR1 was preferentially overexpressed on the M2-like MΦ compared with M1-MΦ, implying that LAIR1 would play a significant role in M2-like MΦ functions. This hypothesis was supported by the finding that *Lair1^–/–^* tumor-bearing mice exhibited a decrease in intratumoral M2-like TAMs, while the total number of MΦ remained unchanged. The result would also suggest that LAIR1 may be involved mainly in modulating the polarization of MΦ from M2 to M1 rather than eliminating the M2-like MΦ cell population entirely. Additionally, we saw an augmentation of activated CD8^+^ and CD4^+^ T cells, NK cells, and DCs in *Lair1^–/–^* versus *Lair1^+/+^* tumors. Our results of testing anti-Lair1 antibody or LAIR1 inhibition by delivering LAIR1 inhibition using our 3-in-1 CAR T cells confirmed these observations. The 3D-printing live imaging showed that M2-like MΦ substantially hindered CAR T cells from interacting with tumor cells. However, anti-Lair1 antibody counteracted the M2-like MΦ-induced inhibition of CAR T cells, leading to an increase in the total number and migratory speed of CAR T cells, a decrease in CAR T cell and M2-like MΦ interactions approximately 35 hours after anti-Lair1 antibody was given, and an increase in CAR T cell–tumor interactions. Consequently, this led to an increased capacity for CAR T cells to engage with and destroy tumor cells effectively. Interestingly, anti-Lair1 antibody also had a similar effect on NAS T cells, albeit to a lesser extent and with slightly distinct kinetics. This observation indicates that anti-Lair1 antibody had a broad effect on T cells, particularly by mitigating inhibitory interactions between T cells and M2-like MΦ. The antigen specificity provided by CAR T cells is essential for strengthening T cell–tumor interactions and promoting significant tumor inhibition. Furthermore, enhanced G-CSF (36) and chemokine (C-C motif) ligand 3 (CCL3) (37) were seen, which respectively facilitated proper T cell recognition and intratumoral trafficking by anti-Lair1 antibody, confirming our hypothesis that LAIR1 is directly involved in M2-like, MΦ-mediated T cell suppression. Our in vivo findings show a promising correlation with the in vitro observations, illustrating that anti-Lair1 antibody extends survival in preclinical models of aggressive tumors, such as GBM and lung cancer, which resist PD-1 blockade. The anti-Lair1 antibody therapy demonstrated superior antitumor efficacy compared with aPD-1 in the models tested. Notably, the synergy achieved by combining anti-Lair1 antibody with CAR T cells highlights its potential as a therapeutic strategy.

A question we ask is whether the results from animal studies can inform clinical translation to humans. A key factor is the biological relevance of LAIR1 across species. Previous studies have identified a conserved collagen-binding site on LAIR1, including in humans and mice (41). Our promising in vivo results in this study and molecular conservation support the pursuit of anti-Lair1 antibody as a therapeutic target in humans. Enhanced antitumor responses were confirmed through scRNA-Seq analysis, which showed a decrease in the number of M2-like TAMs and an increase in M2 macrophage signature genes after anti-Lair1 antibody treatment, suggesting a shift toward an antitumor M1 phenotype. This reprogramming of macrophage polarization was coupled with an increased T cell presence in both spleens and tumors, indicating that anti-Lair1 antibody modulated intratumoral immunity and boosted the systemic immune response. Additionally, distinct outcomes in the 3 pairs of mice treated with IgG or anti-Lair1 antibody suggest that a greater M2-like TAM presence might limit tumor reduction and immunological responses, possibly due to a minimal blocking effect of anti-Lair1 antibody. Interestingly, anti-Lair1 antibody treatment was effective in tumors resistant to chemotherapy, radiation, and aPD-1 therapies, highlighting its potential in targeting immune checkpoints relevant to M2-like TAMs, which may be a suitable strategy for cold cancers like glioma that contain more M2-like TAMs and fewer T cells.

Identifying FXIII-A as a pivotal molecule that affects the function of M2-like MΦ, influenced by LAIR1, provides a vital piece to the intricate puzzle of LAIR1’s mechanism of action, bringing coherence to our understanding of the dynamic pathway involving LAIR1, collagen, and MΦ within the TME. FXIII-A is a blood coagulant protein that has been linked to collagen crosslinking and proliferation (60) and that has mostly been studied in wound healing (61, 62) as well as in tumor progression and metastasis (63). Previous reports showed that in lung squamous cell cancer, there was a notable infiltration of M2-like MΦ with high FXIII-A expression, which was associated with decreased survival (42). Both the mRNA and protein levels of FXIII-A were substantially elevated in M2-like MΦ, suggesting that FXIII-A may serve as a marker for M2-like MΦ, which are a primary cellular source responsible for the abnormal elevation of plasma FXIII-A in patients with malignant solid tumors (64). The role of FXIII-A has been extensively studied in wound healing, where it has been demonstrated to facilitate the cross-linking of fibronectin to collagen and promote collagen proliferation (60–62). We found that the addition of FXIII-A to tumor cultures increased collagen IV levels in the tumor cells. Interestingly, GBMs in *Lair1^+/+^* mice had a shield composed of banded fibril structures of collagen IV — one of the most abundant and protumorigenic macromolecules found in GBMs (65). Our experiments showed that supplementing tumor cultures with FXIII-A led to an increase in collagen IV levels in tumor cells. Notably, *Lair1^+/+^* GBM exhibited a dense network of collagen IV fibrils. This network appeared to form a barrier that impeded T cell access to the tumor. Conversely, in *Lair1^–/–^* GBM, the tumor collagen structure more closely resembled that of normal brain tissue. The reduced collagen fibril shielding around tumor cells can facilitate enhanced interactions between T cells and tumors. Importantly, this effect was also evident in the LLC model following anti-Lair1 antibody treatment, underscoring the potential of LAIR1 inhibition using antibody blockade or 3-in-1 CAR T cells as a clinically relevant therapeutic strategy. Therefore, we have identified an immunosuppressive loop involving LAIR1 in the TME, which can be represented as follows: LAIR1 expression on M2-like MΦ↑ → FXIII-A secretion↑ → collagen IV deposition/proliferation↑ → activation of LAIR1^+^ M2-like MΦ↑ → tumor immunosuppression↑. The LAIR1 knockout or anti-Lair1 antibody effectively disrupted this loop, could reverse the immunosuppression, and enhanced antitumor therapeutics. The intervention using anti-Lair1 antibody resulted in multiple actions, including repolarization of M2-like MΦ to M1 MΦ for an antitumor effect, enhancement of tumor–T cell interaction for cytolytic killing, and reduction of tumor-associated collagen deposition to normalize the TME. These coordinated effects by anti-Lair1 antibody highlight the benefits of targeting these elements within the TME and provide insight into the utility of an integrated approach for better therapeutic intervention in cancer treatment.

In summary, this study elucidates the underlying mechanism of LAIR1 in tumor immunosuppression and presents a strategic approach to targeting LAIR1 through antibody blockade or signaling-deliverable CAR T cells. Given the role of LAIR1 as an immunosuppressive regulator across various cancers, it emerges as a promising focal point for targeted cancer therapy. Our findings have laid the groundwork for ongoing investigational new drug–enabling (IND- enabling) studies focused on developing a humanized LAIR1-blocking antibody or the 3-in-1 CAR T cells for cancer therapies. We believe these represent significant advancements in the pursuit of innovative therapeutic solutions.

## Methods

### Sex as a biological variable.

Our study examined male and female or only female animals in various experiments, and similar findings are reported for both sexes. Sex was not considered a biological variable in our study.

### Study design.

Our study presents findings on the underlying mechanism of LAIR1 in tumor immunosuppression and the strategies for targeting LAIR1 in cancer. We conducted in vitro and in vivo studies to assess how the function of the tumor microenvironment was influenced by LAIR1 and how therapies with LAIR1 inhibition affect the antitumor response of preclinical animal models. For the animal studies, detailed information on the cell and mouse models can be found in the respective methods sections below. The exact sample sizes used were based on our previous studies, as indicated in the experimental schemas and figure legends. Allocation was not randomized, as the experimental groups were separated by in vivo imaging systems (IVIS) to ensure uniformity in tumor size across groups before treatment commenced. The IVIS (Xenogen) and survival data analyses for antitumor efficiency were performed in a blinded manner. More replicates were included for readouts associated with greater variation. All in vivo experiments were approved and performed following the guidance of the IACUC of the University of Florida. The health status of each mouse was monitored daily, and mice that met the predefined humane endpoints were euthanized. All in vivo experiments were performed at least twice to achieve reproducibility.

### Cell lines.

The murine GBM cell lines KR158B-luciferase (KR158B-Luc) were provided courtesy of Karlyne M. Reilly at the National Cancer Institute (NCI), NIH (66, 67), and GL261-Luc, another GBM line, was purchased from DSMZCellDive (ACC802) and overexpressed with luciferase (Addgene, 17477). The murine LLC1 cells were provided by Christian Jobin’s laboratory at the University of Florida. The retroviral packaging lines GP2-293 (Takara Bio, 631458) and 293T/17 (American Type Culture Collection [ATCC], CRL-11268) were obtained. To overexpress CD70 and/or luciferase, the tumor lines were transduced by a lentiviral vector containing these genes (Addgene, 17477). The process of lentiviral transduction was described in our previous report (39). The human GBM cell line U87 was purchased from ATCC (HTB-14). The human primary GBM-derived cell line pGBM#1 was obtained from The Florida Center for Brain Tumor Research (FCBTR). The human acute monocytic leukemia line THP-1 was obtained from ATCC (TIB-202).

### Retroviral and lentiviral constructs.

The retroviral pMSGV8-CD70CAR-CXCR2 (8R-70CAR) (human) and pMSGV8-CD70CAR (CD70CAR) (mouse) constructs were previously described in our previous reports (34, 39). Human LAIR2 was synthesized by Integrated DNA Technologies (IDT) and subsequently subcloned downstream of the 8R-70CAR construct, which was linked with a modified 2A peptide (T2A) to generate constructs of pMSGV8-CD70CAR-CXCR2-LAIR2 (L2-8R-70CAR) (human). For the CD70-Luc lentiviral construct, the pLenti-CMV-Puro-LUC vector was purchased from Addgene (plasmid no. 17477), and the cDNA of CD70 was subcloned into the vector.

### Mice.

C57BL/6 (*Lair1^+/+^*, The Jackson Laboratory, 000664) and B6.Cg-Lair1tm1.1Jco/J (*Lair1^–/–^*, The Jackson Laboratory, 032788) mice (68) were used in this study. *Lair1^–/–^* mice were bred by the Animal Care Services (ACS) staff at the University of Florida, and genotyping for LAIR1 expression was performed by Transnet YX. Six- to 8-week-old male and female mice were implanted with murine GBM tumors via intracranial (i.c.) injection, using either 1 × 10^4^ KR158B-Luc, 1 × 10^4^ KR158B-CD70-Luc, or 1 × 10^5^ GL261-Luc cells (34). Inoculation of the murine LLC1 line was performed by s.c. injection at a dose of 1 × 10^4^ cells per mouse (69). Tumor size was assessed using 2 methods: IVIS imaging for brain tumors, with size calculated on the basis of the average radiance (p/s/cm²/sr) within the region of interest (ROI) for quantitative luminescence analysis (70), and caliper measurements for LLC1 tumors (71). Tumor volume for LLC1 tumors was determined using the ellipsoidal formula: volume (V) = ½ (width^2^ × length) (69).

### Statistics.

Statistical analysis was performed using GraphPad Prism 9 (GraphPad Software, RRID: SCR_002798) and R statistical programming software (version 4.4.1, R Core Team, 2021). All 2-group comparisons were assessed using the 2-sample *t* test or the Wilcoxon rank-sum test, as appropriate. Generalized estimating equation (GEE) models were used to analyze data with repeated measurements and to account for the number of biological replicates. Survival data were analyzed using the Kaplan-Meier method, with the log-rank test applied for 2-group comparisons. To control for multiple comparisons, the FDR correction was applied. A *P* value of less than 0.05 was considered statistically significant.

### Study approval.

In murine studies, mice were handled in accordance with the University of Florida animal care policy, and all protocols of the studies were approved by University of Florida’s IACUC (study no. 201809104, PI: J. Huang, T Cell Therapy Against Solid Tumors, and IACUC study no. 202111479, PI: J. Huang, Cancer Immunotherapy Therapy Using Lair1 Blockade). All constructs used in this report were evaluated and approved by the University Florida’s Division of Environmental Health and Safety, Biological Safety Office (RD-4231, PI: J. Huang, The Role of CD70 in Antitumor Response). Human materials were handled according to federal regulations and approved by the UF’s IRB (study IRB no. 201400101, PI: J. Huang, Testing the Function of Human Immune Cells Using Isolated PBMC from Healthy Donors).

### Data availability.

All data needed to evaluate the conclusions in this study are present in this article and/or the supplemental materials. (a) TCGA (72): UCSC Xena was used in this study, and TCGA Glioblastoma study was selected (a total of 631 samples) for analysis, in which 5 samples were of normal brains, 152 samples were of primary GBMs, and 12 samples were of recurrent GBMs. *Lair1* gene expression was measured, and a heatmap was downloaded from the UCSC Xena website (https://xena.ucsc.edu/). (b) GTEx Portal data were used for the analyses described in Supplemental Figure 1 (data were accessed August 5, 2023) and included data on bulk tissue gene expression for *LAIR1* (ENSG0000016761 3.15) and *CD274/PDL1* (ENSG0000012021 7.13). (c) The GBMSeq (31) website (http://www.gbmSeq.org/) was used to determine cell types expressing LAIR1 in GBM. This database can help identify infiltrating neoplastic cells and distinct myeloid cell populations in the tumor core and surrounding areas. The scRNA-Seq data included those from tissues from a cohort of 4 patients with GBM. LAIR1 data were specifically acquired and downloaded for our study. (d) The Brain Immune Atlas database (33) (https://www.brainimmuneatlas.org/) was also used in our research. This database offers information on human and mouse brain immune cells in both healthy and diseased states, including GBM (7 newly diagnosed and 4 recurrent human GBMs and 3 GL261 murine GBMs). CD45^+^ cells in these tumors were isolated and analyzed by scRNA-Seq. We obtained and downloaded data on *LAIR1* expression distribution among these cell types. (e) The scRNA-Seq data generated in this study have been deposited in the open-access BioProject database at https://ngdc.cncb.ac.cn/bioproject/browse/PRJCA038133 Reagents and animal models used in this study are available upon request and through an institutional material transfer agreement. The remaining data are available within the article. Values for all data points are provided in the Supporting Data Values file. Additional details on methods can be found in the Supplemental Methods.

### Code availability.

No custom code was created. All the packages used in this study are open-source R packages. The demultiplexed cells were aligned to the mouse mm10 genome using Cellranger 7.0. Subsequently, the sequencing data quality control, normalization, feature selection, dimension reduction, and visualization were analyzed using Seurat 4.0. SingleR 2.4.1 was applied for the scRNA-Seq cell type annotations. Moreover, CellChat 2.1.0 was utilized to explore cell-cell interactions. To identify differentially expressed genes (DEGs) on specific cell types between groups, the Limma-voom 3.58.1 method was applied. Subsequently, ggplot2 3.4.1 was used to generate relevant plots.

## Author contributions

HT and JH conceived and designed the study. HT, DC, DTN, GA, CVR, RL, LJ, AP, DIP, AC, MA, HRMG, CX, DJ, KLJ, PC, JAL, LPD, WGS, and JH developed the study methodology. HT, DC, DTN, CVR, RL, T Liu, AYH, NAP, RWD, JZ, LJ, AP, DIP, AC, HRMG, GA, AK, FW, DJ, CW, KLJ, MA, PC, JAL, LPD, and WGS performed experiments. HT, CY, DTN, DIP, TG, T Lin, PW, SY, CX, MOG, EKM, and JH analyzed and interpreted the data. HT, MA, APG, PC, JAL, APG, MR, EJS, BYSK, LPD, DAM, WGS, and JH wrote, reviewed, and revised the manuscript. HT and JH were responsible for project administration. JH supervised the study.

## Supplementary Material

Supplemental data

Unedited blot and gel images

Supplemental video 1

Supplemental video 2

Supplemental video 3

Supplemental video 4

Supporting data values

## Version 1 07/01/2025

In-Press Preview

## Version 2 08/15/2025

Electronic publication

## Version 3 09/10/2025

Typo corrected in correspondence address

## Version 4 07/07/2026

Added to the conflict of interest statement
