# Supplementary material for: Targeting immune checkpoint LAIR1 with antibody blockade or 3-in-1 CAR T cells enhances antitumor response
Source: J Clin Invest. 2025 Jul 1;135(16):e184043. doi: 10.1172/JCI184043 (PMC12352909; doi:10.1172/JCI184043)
Supplement: Supplemental data [file jci-135-184043-s007.pdf]

Supplemental Materials for

Blocking immune checkpoint LAIR1 with antibody blockade or 3-in-1 CAR T cells enhances antitumor response

Haipeng Tao et al.

*Corresponding author. Email: jianping.huang@neurosurgery.ufl.edu

This PDF file includes:

Supplemental Figures 1 to 15
Legend for Videos 1 to 4
Gating Strategy 1 to 9
Supplemental Methods
Supplemental Table 1

Other Supplementary Materials for this manuscript include the following:

Videos 1–4
Supporting data values
Unedited blot images

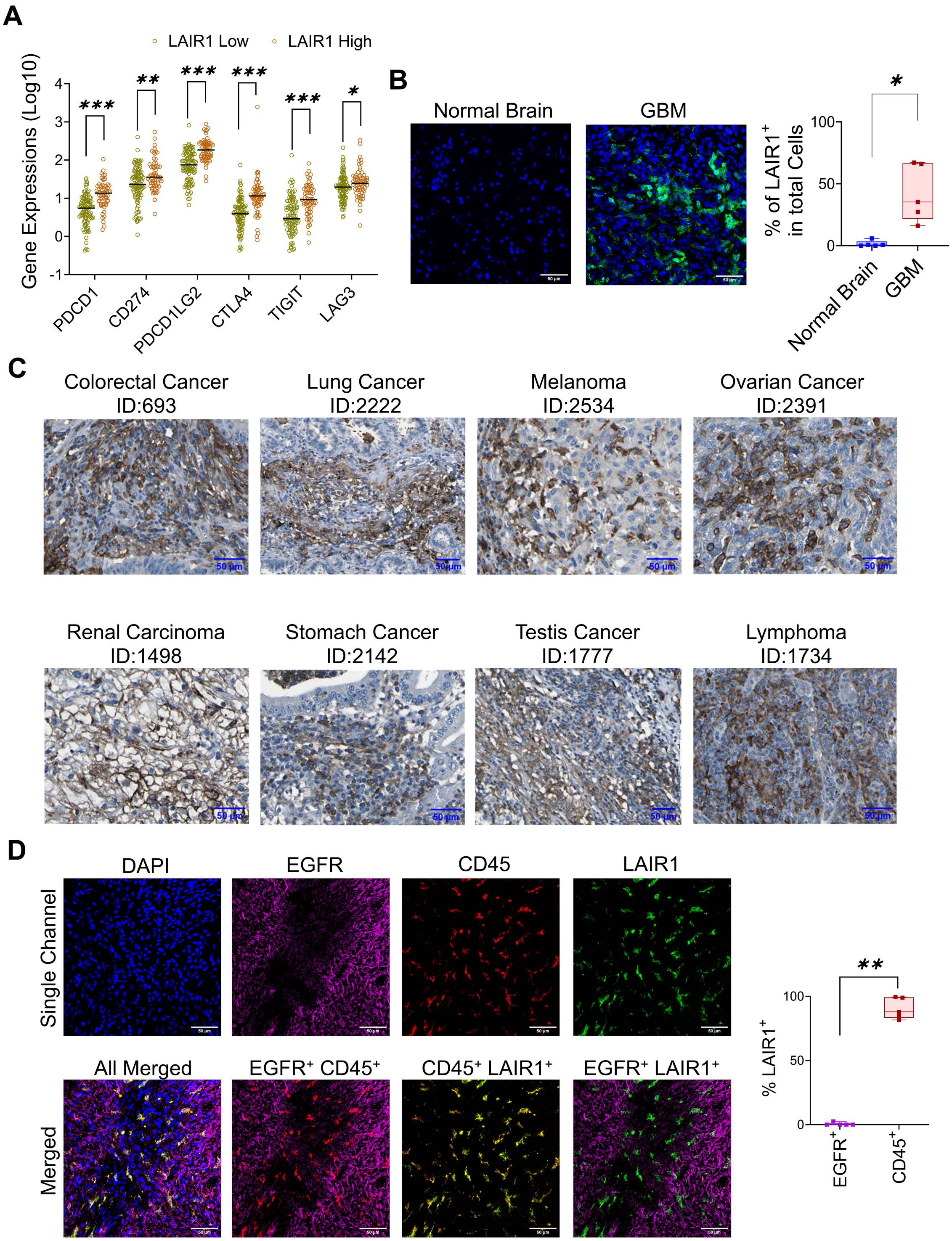
A
LAIR1 Low
LAIR1 High
Gene Expressions (Log10)
PDCD1
CD274
PDCD1LG2
CTLA4
TIGIT
LAG3
B
Normal Brain
GBM
% of LAIR1+ in total Cells
Normal Brain
GBM
C
Colorectal Cancer ID:693
Lung Cancer ID:2222
Melanoma ID:2534
Ovarian Cancer ID:2391
Renal Carcinoma ID:1498
Stomach Cancer ID:2142
Testis Cancer ID:1777
Lymphoma ID:1734
D
DAPI
EGFR
CD45
LAIR1
Single Channel
All Merged
EGFR+ CD45+
CD45+ LAIR1+
EGFR+ LAIR1+
Merged
% LAIR1+
EGFR+
CD45+

Supplemental Figure 1

LAIR1 expressing cells are present in tumors, and they are on CD45^{+} cells. (**A**) LAIR1 gene expression is correlated with other immune checkpoints, including PDCD1, CD274, PDCD1LG2, CTLA4, and TIGIT. RNA-seq data were analyzed using the TCGA dataset (n=172). The median value of LAIR1 gene expression was used to separate the LAIR1^{high} and LAIR1^{low} groups, and the indicated marker expressions [as in log2(x+1) transformed RSEM normalized count] were analyzed. (**B**) IF staining revealed LAIR1-expressing cells in 5 surgically resected primary GBM tumors (one is shown on the left), and 5 normal brains were utilized as a control. (**C**) IHC staining revealed LAIR1-expressing cells present in other cancer tissues. The images were acquired from the Human Protein Atlas (https://www.proteinatlas.org/). The origins of each image are documented in the Materials and Methods section. The depicted images are focused on regions containing LAIR1-positive cells. (**D**) LAIR1 expression is predominantly on CD45^{+} cells, not tumor cells. IF imaging of one of 5 distinct human GBM samples shows staining with LAIR1, EGFR (tumor marker), and CD45 (immune marker) (left). Quantitative analysis (right) summarizes the percentages of EGFR^{+}LAIR1^{+} and CD45^{+}LAIR1^{+} cells. These samples were stained using specific antibodies for CD45, EGFR, and LAIR1, with DAPI used for nuclear staining. Data are represented as Interleaved scatter with median in **A**, Violin plot with bar in **B** and **D.** Statistical significance for the presented findings was determined using the Mann–Whitney U test. $^{*}p < 0.05$, $^{**}p < 0.01$, $^{***}p < 0.001$.

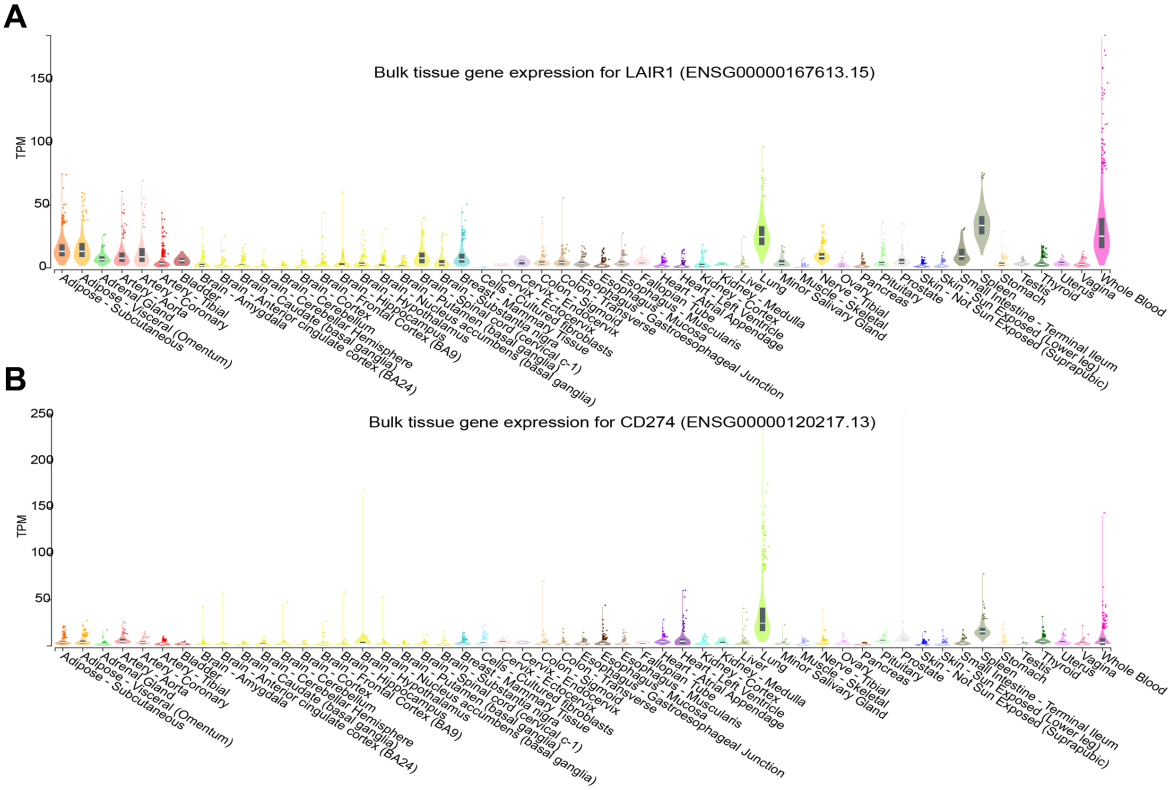

Supplemental Figure 2

Gene expression pattern of LAIR1 on normal tissues is similar to CD274 (PD-L1). Gene expression of LAIR1 (**A**) and CD274 (PD-L1) (**B**) in various normal tissues. The data were obtained from the GTEx Portal for LAIR1: https://gtexportal.org/home/gene/LAIR1, and CD274: https://gtexportal.org/home/ gene/CD274.

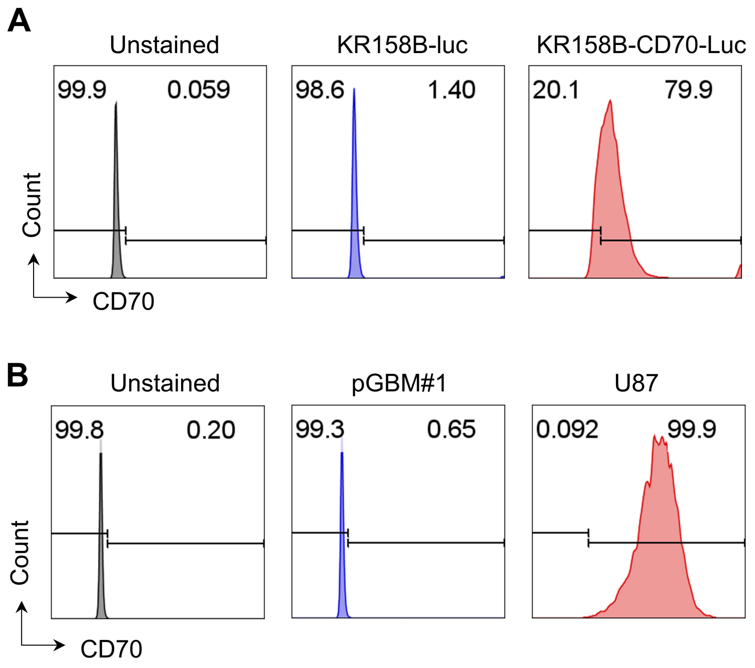

Supplemental Figure 3

CD70 expression on tumor lines. (**A**) Flow cytometry (FC) analysis of CD70 expression on murine GBM cell lines KR158B and KR158B-CD70-Luc. (**B**) FC analysis of CD70 expression on human GBM lines pGBM#1 and U87 lines.

A

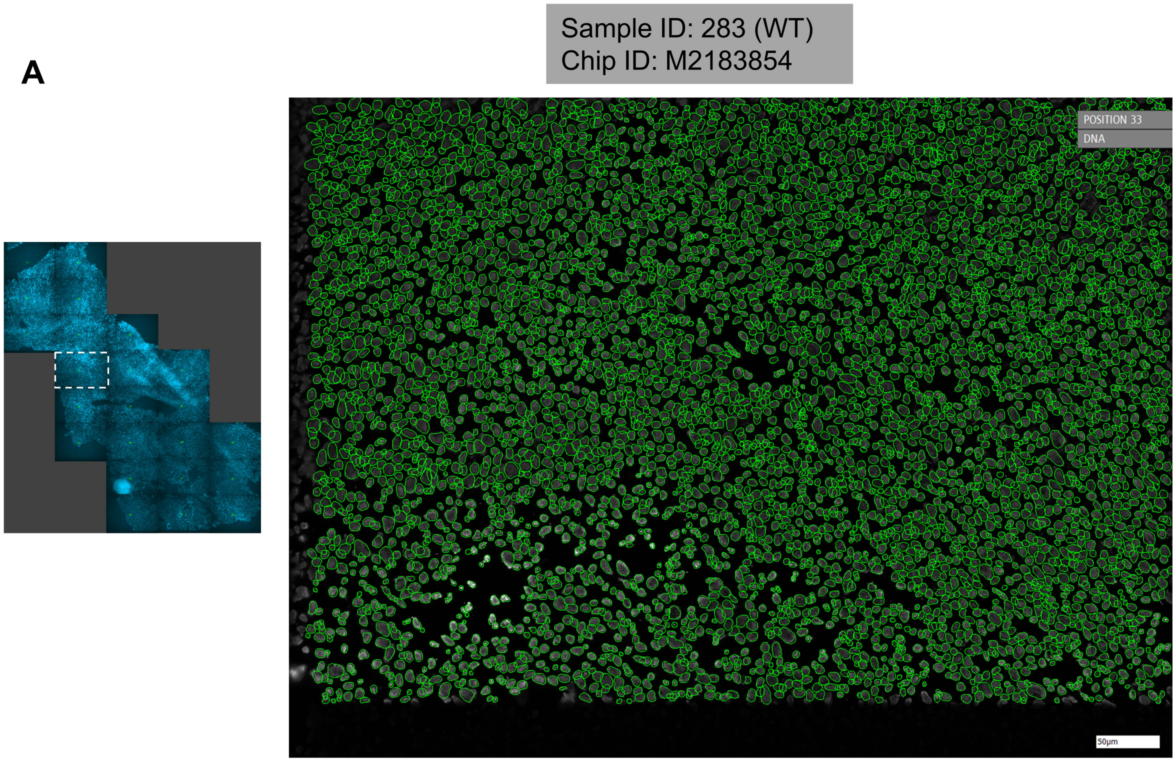

Sample ID: 283 (WT)
Chip ID: M2183854
POSITION 33
DNA
50μm

B

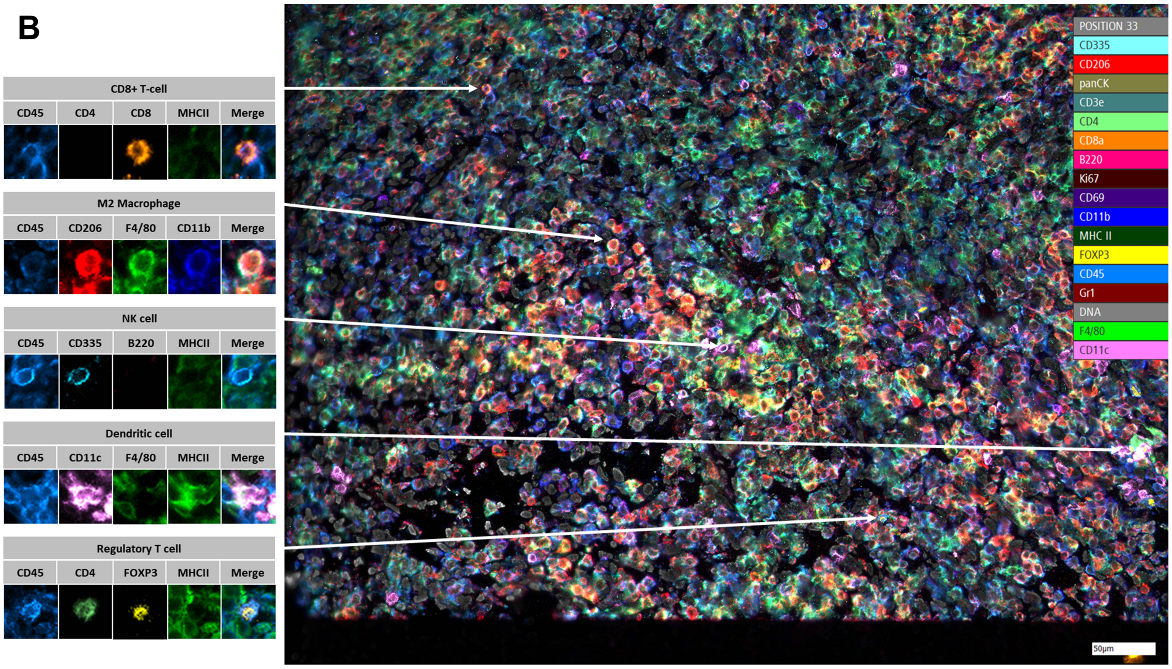

CD8+ T-cell
CD45
CD4
CD8
MHCII
Merge
M2 Macrophage
CD45
CD206
F4/80
CD11b
Merge
NK cell
CD45
CD335
B220
MHCII
Merge
Dendritic cell
CD45
CD11c
F4/80
MHCII
Merge
Regulatory T cell
CD45
CD4
FOXP3
MHCII
Merge
POSITION 33
CD335
CD206
panCK
CD3e
CD4
CD8a
B220
Ki67
CD69
CD11b
MHC II
FOXP3
CD45
Gr1
DNA
F4/80
CD11c
50μm

Supplemental Figure 4

Working flow used in **Figure 2H**. (**A**) A rectangular area of the sample was initially scanned, and regions-of-interest (ROI) and tissue integrity were assessed based on auto-fluorescence in pre-stain QC. Due to the small overall area of the tissue, all areas containing tissue were selected for subsequent scans. DNA stain was used as the basis for automated cell segmentation to identify cells in each sample, allowing cell populations to be identified and quantified. The image shows a DNA stain with segmented cells outlined in green. (**B**) After the selection of ROI based on autofluorescence images, all 17 markers were subsequently stained in cycles. The image to the right shows an overlay of all marker stains in pseudo colors. Fluorescence intensities are automatically calculated for each cell and each marker.

Sample: 283
Chip M2183854

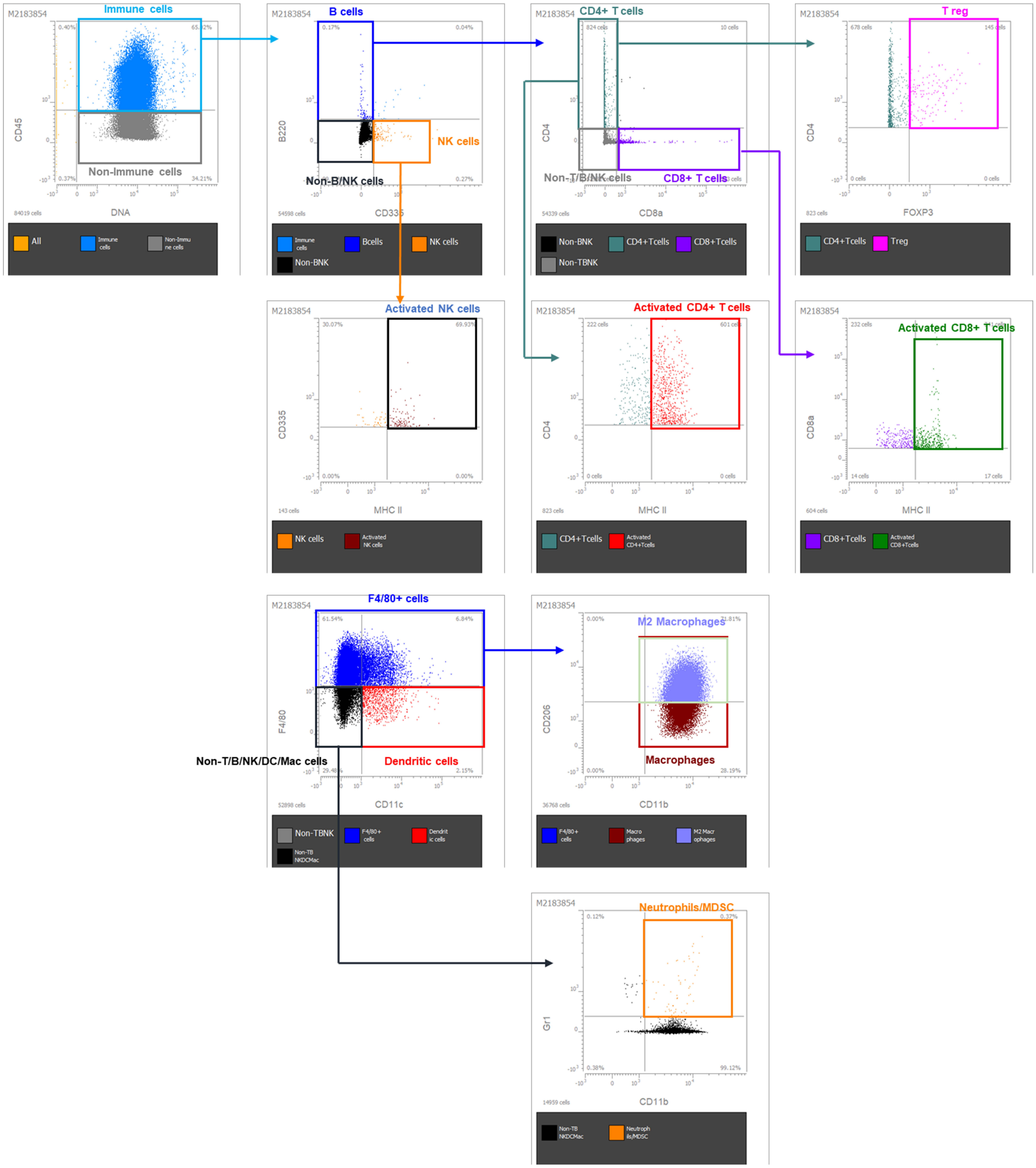

Supplemental Figure 5

The gating strategy in Figure 2H. After automatic segmentation and fluorescence quantification, cell type identification was carried out based on a bivariate index-sorting method, which is very similar to flow cytometry. Cells are assigned to specific phenotype gates guided based on the expression of defining markers. Phenotype population gates are then used as parent gates for further sub-phenotyping. This results in a hierarchical gating strategy followed until all desired population gates are quantified.

B-cells: $CD45^{+}B220^{+}CD335^{-}$;

NK-cells: $CD45^{+}B220^{-}CD335^{+}$;

CD4+ T-cells: $CD45^{+}B220^{-}CD335^{-}CD4^{+}CD8^{-}$;

CD8+ T-cells: $CD45^{+}B220^{-}CD335^{-}CD4^{-}CD8^{+}$;

T-reg: $CD45^{+}B220^{-}CD335^{-}CD4^{+}CD8^{-}FOXP3^{+}$;

Activated $CD4^{+}$ T-cells: $CD45^{+}B220^{-}CD335^{-}CD4^{+}CD8^{-}MHCII^{+}$;

Activated $CD8^{+}$ T-cells: $CD45^{+}B220^{-}CD335^{-}CD4^{-}CD8^{+}MHCII^{+}$;

Dendritic cells: $CD45^{+}B220^{-}CD335^{-}CD4^{-}CD8^{-}CD11c^{+}F4/80^{-}$;

Macrophages: $CD45^{+}B220^{-}CD335^{-}CD4^{-}CD8^{-}F4/80^{+}CD11b^{+}$;

M2 Macrophages: $CD45^{+}B220^{-}CD335^{-}CD4^{-}CD8^{-}F4/80^{+}CD11b^{+}CD206^{+}$;

Neutrophils/MDSCs: $CD45^{+}B220^{-}CD335^{-}CD4^{-}CD8^{-}CD11c^{-}F4/80^{-}CD11b^{+}Gr1^{+}$.

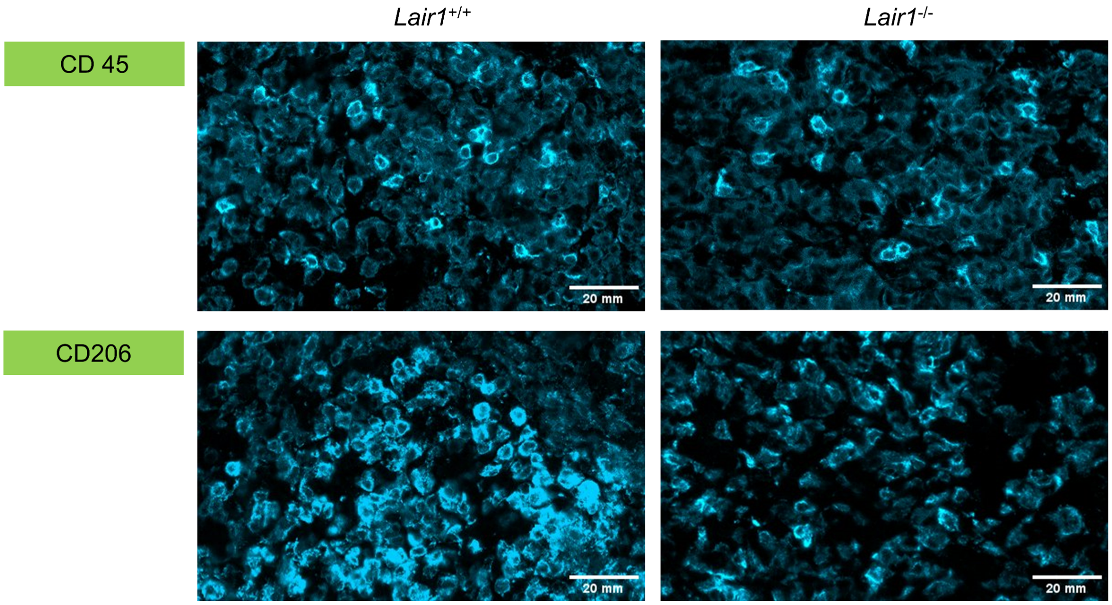

Supplemental Figure 6

Exemplary staining patterns illustrate stain signals from both *Lair1*$^{+/+}$ and *Lair1*$^{-/-}$ **and** *Lair1*$^{-/-}$ **tumors, as presented in Figure 2H.** Comparable expression levels were observed for CD45^{+} and CD206.

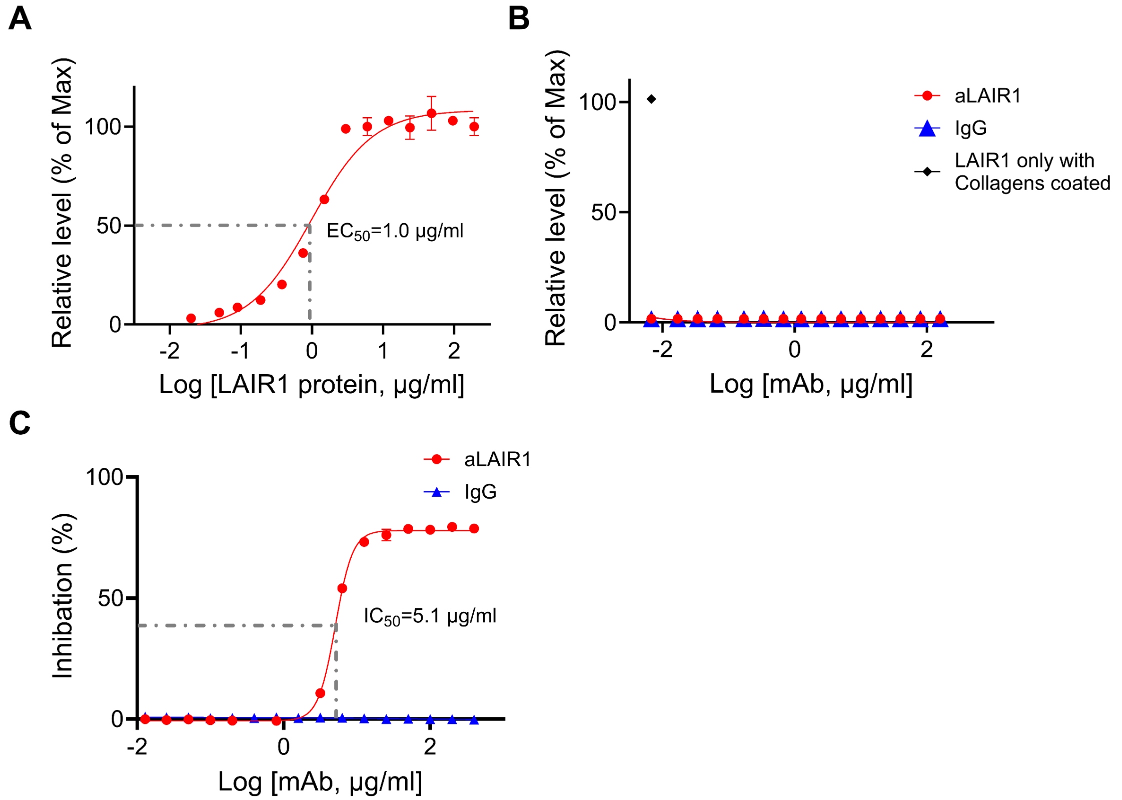

Supplemental Figure 7

Human aLAIR1 inhibits LAIR1–collagen interaction. (A) Soluble human LAIR1-His protein binds specifically to collagens. Increasing concentrations of LAIR1-His were incubated on collagen I/IV–-coated plates (10 µg/ml) for 2 hours at room temperature. Binding was quantified by His-tag ELISA, confirming the dose-dependent interaction between LAIR1 and collagens. EC_{50} was identified. (**B**) LAIR1-His showed no binding to uncoated plates, confirming its specific interaction with collagen. LAIR1-His (1 µg/ml) was pre-incubated with increasing concentrations of human aLAIR1 or IgG for 1 hour, then added to uncoated plates for 2 hours. Three collagen-coated wells served as maximal (Max) binding controls. Binding was quantified by His-tag ELISA and reported as % response relative to the LAIR1-only collagen-coated control. **(C)** aLAIR1 effectively inhibits LAIR1–collagen binding in a dose-dependent manner. Pre-incubation of LAIR1-His with aLAIR1 or IgG was performed as described in **B**, followed by incubation on collagen I/IV-coated plates for 2 hours at room temperature. Binding was quantified by His-tag ELISA. The % of inhibition was calculated as: 1 – (% of the relative level). All experiments were independently repeated at least twice. Data are presented as mean ± SD. The IC_{50} value was calculated by nonlinear regression using a four-parameter logistic model.

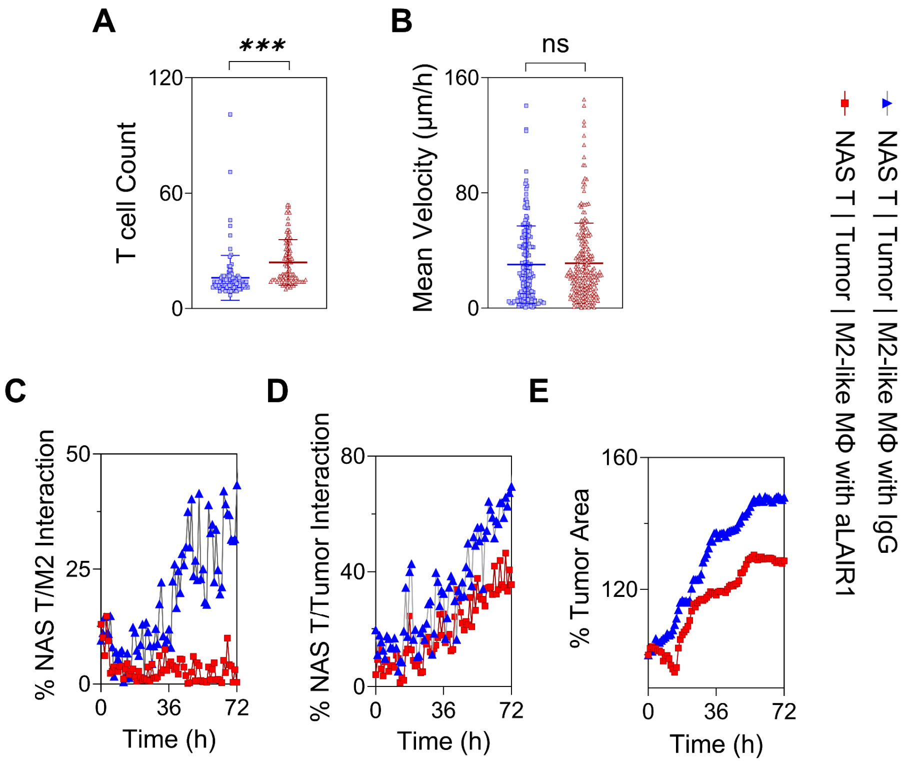

Supplemental Figure 8

Impact of aLAIR1 on Non-Antigen Specific (NAS) T cells in the presence of M2-like MΦ within the 3D printing system (The experimental design is shown in Figure 3D, and the data were calculated from **Vides 1**, **C** and **D**). (**A** and **B**) The total number or the mean velocity of NAS T cells in the presence of IgG or aLAIR1. (**C** and **D**) The kinetic change in % of NAS T Cell/Tumor interaction in the presence of IgG or aLAIR1. The parameter was measured by the overlapped region of NAS T/ tumor or CAR T/tumor signals as a percent of total T cells. (E) No impact on tumor inhibition for IgG and aLAIR1. The tumor area was measured as a percent of the original tumor mass. Data are represented as Mean ± SD in **A** and **B**; Points and connecting lines in **C-E**. Statistical significance for the presented findings was determined using the two-sample t-test in **A** and **B**. Generalized estimating equation (GEE) models in (**C** and **E**, $p < 0.001$, between IgG and aLAIR1). ***$p < 0.001$.

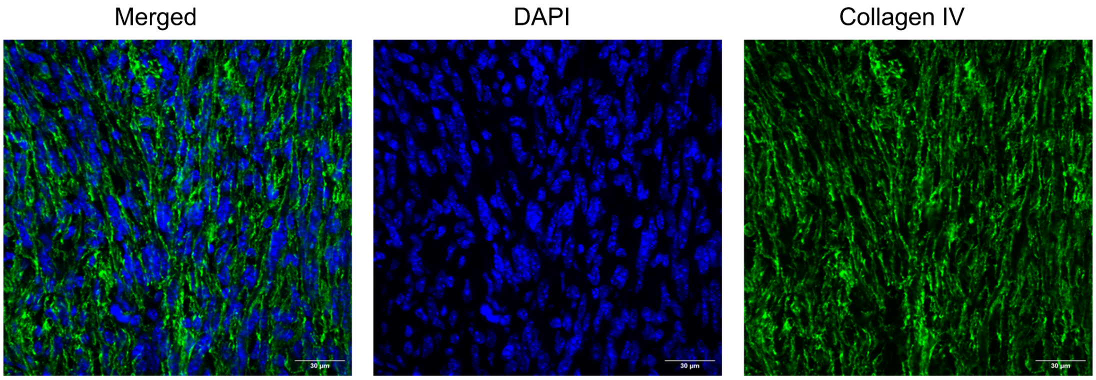

Supplemental Figure 9

A Representative image of collagen IV in the 2D model. Collagen IV expressions in tumor from **Figure 6C** before 3D reconstruction**.** Collagen IV (green) structure and DAPI (blue) for nuclei staining.

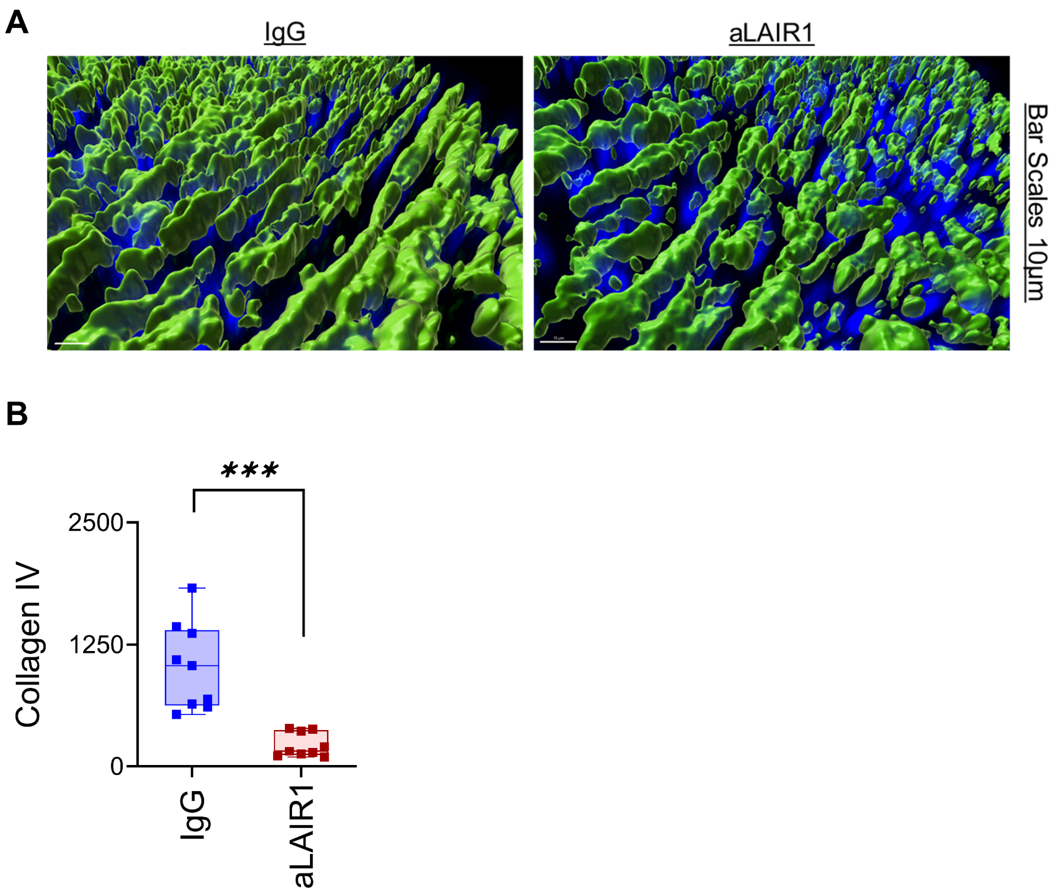

Supplemental Figure 10

Collagen IV expression in IgG and aLAIR1 treated GBM model. (**A** and **B)** Nine tumor tissue sections collected from 3 *Lair1*$^{+/+}$ KR158B-CD70-Luc GBM TB-mice 63 days post-treatment, respectively, described in **Figure 4A**, were stained with IF for collagen IV (green) and DAPI (blue) nuclei staining. The comparison of collagen IV fibers in relationship with tumor nuclei for the aLAIR1 and IgG control groups from representative images and quantitative comparisons are shown. Data are represented as a violin plot in **B.** Statistical significance for the presented findings was determined using the GEE model. ***$p < 0.001$.

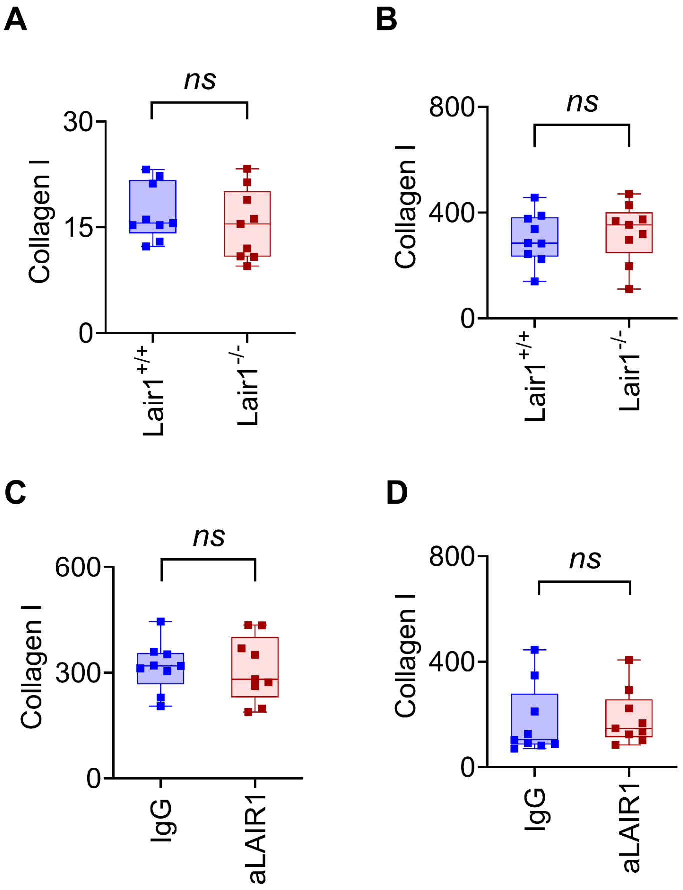

Supplemental Figure 11

LAIR1 inhibitions do not alter collagen I in normal brains and tumors. aLAIR1 doesn't impact collagen I expressions. The fluorescent intensity of collagen I in normal brains and tumors (n=9) was measured by IF. (**A-E**) Comparison of collagen I in normal brains using the same tissues in **Figure 6A** and tumors used in **Figure 6C** (GBM, $Lair1^{-/-}$) and **E** (LLC1, aLAIR1 treatment), and **Supplemental Figure 10** (GBM, aLAIR treatment). The expression of collagen I was measured by MFI. Data are represented as violin plots**.** Statistical significance for the presented findings was determined using the GEE model. *ns:* Not significant.

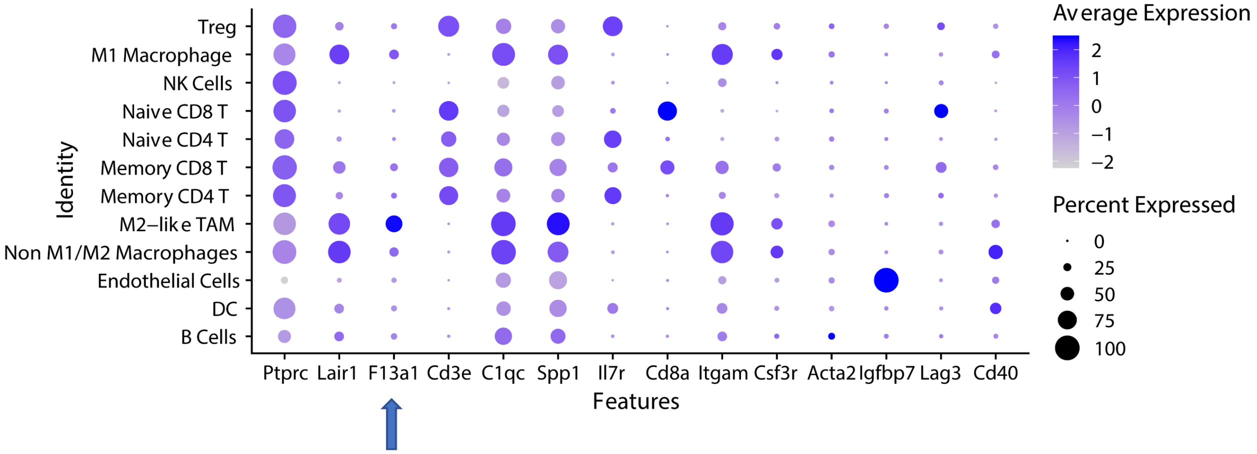

Supplemental Figure 12

Cell population identification and signature gene expressions. The dot plot defined the cell populations in scRNA seq described in **Figure 6, G-J** and **Supplemental Figure 13 B, C,** and **F**. The Y-axis depicts the names of the cell types; the X-axis depicts the gene identity. Expression is noted according to the number of cells that express the marker above the background (the diameter of the circle increases with the fraction of cells expressing the marker); The average intensity is represented by a color gradient varying from white (no expression) to purple (mid-intensity expression) and to blue (maximum expression). The F13a1 is indicated by the blue arrow.

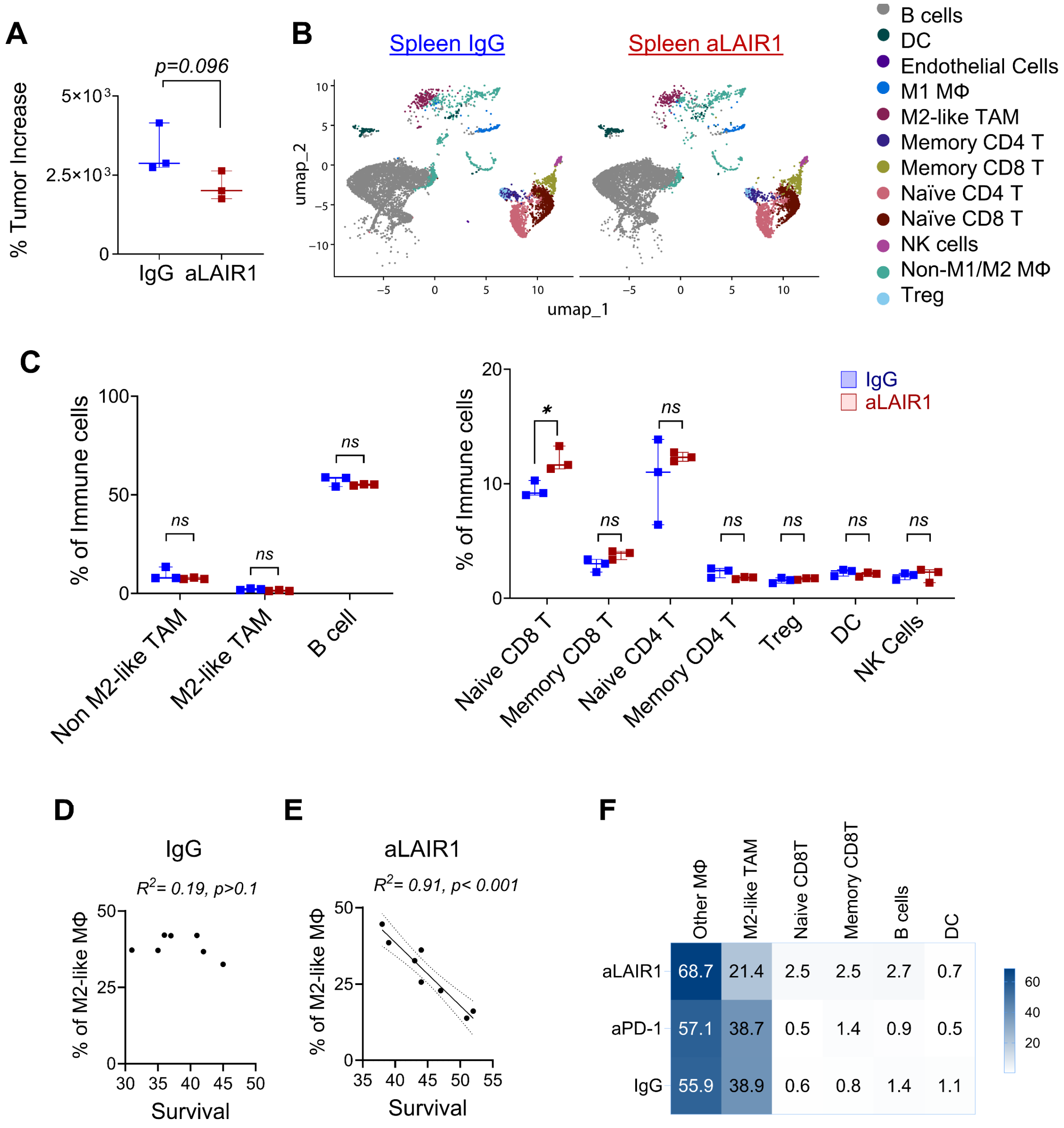

Supplemental Figure 13

aLAIR1 modulates immune cells in peripheral tissues and tumors. (**A**) C57BL/6 TB-mice (KR158B-CD70-Luc, 3/group) received IgG or aLAIR1 treatment (**Figure 4A**). Tumor increase after the treatment was calculated 48 days post-treatment using IVIS signals related to the baseline (day -7). (**B-C**) The tumor (**Figure 6G-H**) and spleen tissues were analyzed by scRNA-seq 49 days after treatment. UMAPs illustrate immune cell clusters from spleens (**B**), with population summaries (>1%) shown in **C**. (**D-E**) Female C57BL/6 mice (8/group) were implanted i.c. with KR158B-Luc (1×10^4 cells/mouse) and treated with 5 doses of aLAIR1 or IgG (200 µg/dose, i.p., every other day from day 0 to 8). Survival was monitored, and tumors were harvested at the endpoint. M2-like TAM frequencies were quantified by FC and correlated with survival by regression analysis. (**F**) The same tumor model as in (**D-E**) was treated with IgG, aLAIR1, or aPD-1 (200 µg/dose, 3/group, 5 doses every other day). Tumors were collected on day 28, and pooled cells from each group were analyzed by scRNA-seq. Cell populations >1% are summarized. Data are represented as Box-and-whiskers plots (**A** and **C**). Statistical significance was determined using two-sample t-tests (**A** and **C**), and Pearson analysis (**D-E**). ns: Not significant, $^* p < 0.05$.

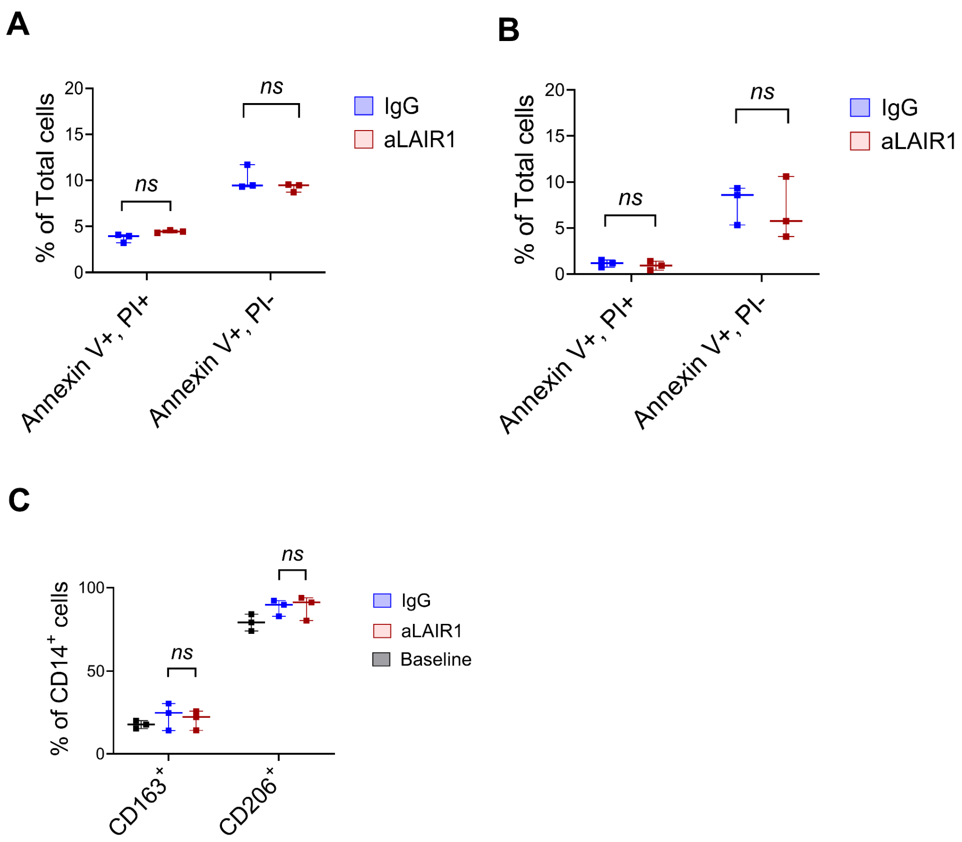

Supplemental Figure 14

aLAIR1 does not influence the apoptosis and phenotyping of LAIR1-expressing cells. (A and B) The apoptosis of LAIR1-expressing cells (n=3) was not impacted by aLAIR1. Human THP-1 cells (a LAIR1-expressing human monocyte line) and human PBMC-induced M2-like MΦ were treated daily with 5 μg/ml of IgG or aLAIR1 for 3 days; the viability of these cells was assessed by flow cytometry using Annexin V and Propidium Iodide (PI). Annexin V^{+}PI^{-} cells represent early apoptosis cells and Annexin V^{+}PI^{+} cells are late apoptosis cells. (**C**) The phenotyping of M2-like MΦ in **B** was assessed by flow cytometry using CD163 and CD206. Data are represented as violin plots. A two-sample t-test was used to compare. FDR correction was applied in **C**. *ns:* Not-significant

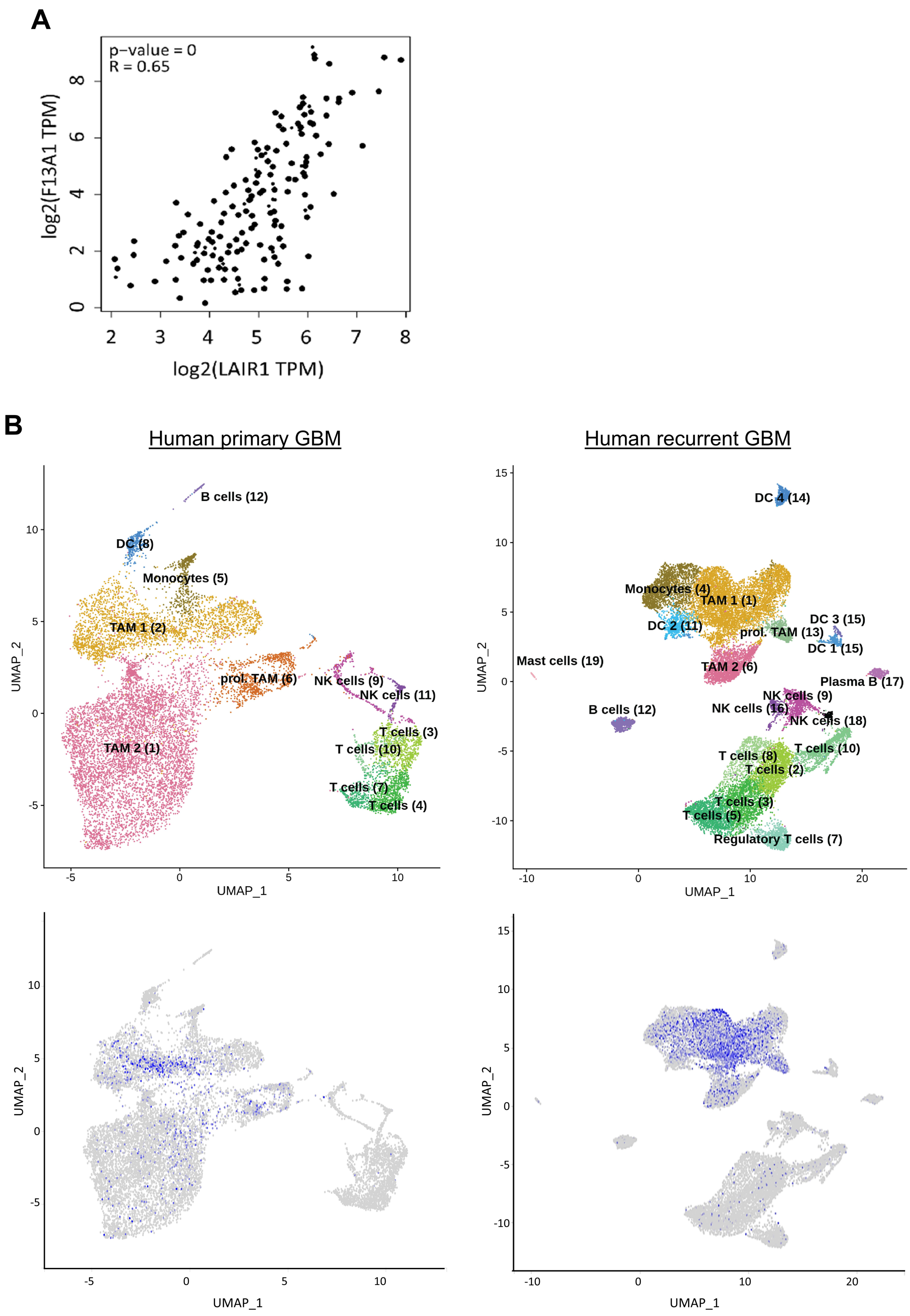

A
p−value = 0
R = 0.65
log2(F13A1 TPM)
log2(LAIR1 TPM)
B
Human primary GBM
Human recurrent GBM
B cells (12)
DC (8)
Monocytes (5)
TAM 1 (2)
prol. TAM (6)
NK cells (9)
NK cells (11)
T cells (3)
T cells (10)
T cells (7)
T cells (4)
TAM 2 (1)
DC 4 (14)
Monocytes (4)
TAM 1 (1)
DC 3 (15)
DC 2 (11)
prol. TAM (13)
DC 1 (15)
Mast cells (19)
TAM 2 (6)
Plasma B (17)
NK cells (9)
B cells (12)
NK cells (16)
NK cells (18)
T cells (10)
T cells (8)
T cells (2)
T cells (3)
T cells (5)
Regulatory T cells (7)
UMAP_1
UMAP_2

Supplemental Figure 15

F13a1 expression in GBM patients. (**A**) Regression analysis between F13a1 and LAIR1 gene expressions in GBM was analyzed using the Pearson correlation coefficient from the TCGA database(http://gepia.cancer-pku.cn/detail.php?gene=lair1). (**B**) The UMAP of F13a1 expressing cells represents TAM (M2-like MΦ) in primary (left) and recurrent (right) GBM using the Brain Immune Atlas database (https://www. brainimmuneatlas.org/index.php).

Legend for Videos 1–4

Real-Time Monitoring of aLAIR1 Reversing M2-Like Macrophage-Mediated Inhibition of CAR T Cells in a 3D Model. Both 8R-CD70CAR T cells and Non-Antigen Specific (NAS) T cells, as well as M2-like MΦ, were derived from the same human PBMC. The labeled M2-like MΦ (green) and 8R-70CAR T or NAS T cells (red) were co-cultured with the $CD70^{+}$U87 GBM line (blue-white) (cell ratio=5:1:2.5). Cultures were treated daily with either IgG or aLAIR1 (5 μg/ml/day) over 105 hours.

The experimental conditions were as follows:

(A) 1. 8R-70CAR T cells + M2-like MΦ + Tumors + aLAIR1
2. 8R-70CAR T cells + M2-like MΦ + Tumors + IgG
3. NAS T cells + M2-like MΦ + Tumors + aLAIR1
4. NAS T cells + M2-like MΦ + Tumors + IgG

Quantitative results are presented in **Figure 3G-K** and **Supplemental Figure 8A-E**.

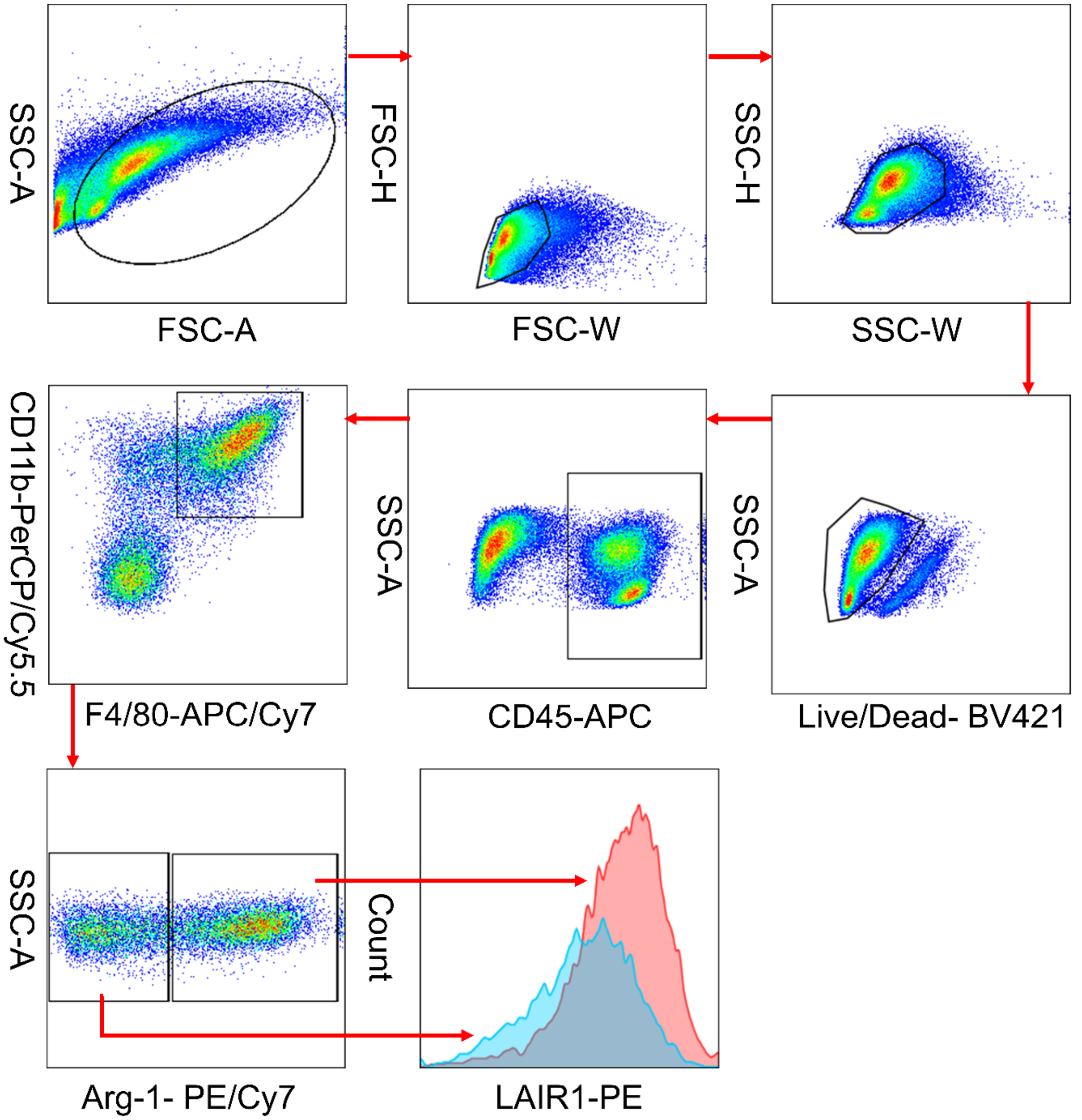

Gating Strategy 1

Gating strategy for Figure 1, **F** and **G.** Detection of the relative % of Arg-1$^+$ M2-like TAM in the total CD45$^+$ cells in **Figure 1F** and MFI of LAIR1 in M2-like TMA and Mon M2-like TAM depicted in **Figure 1G**.

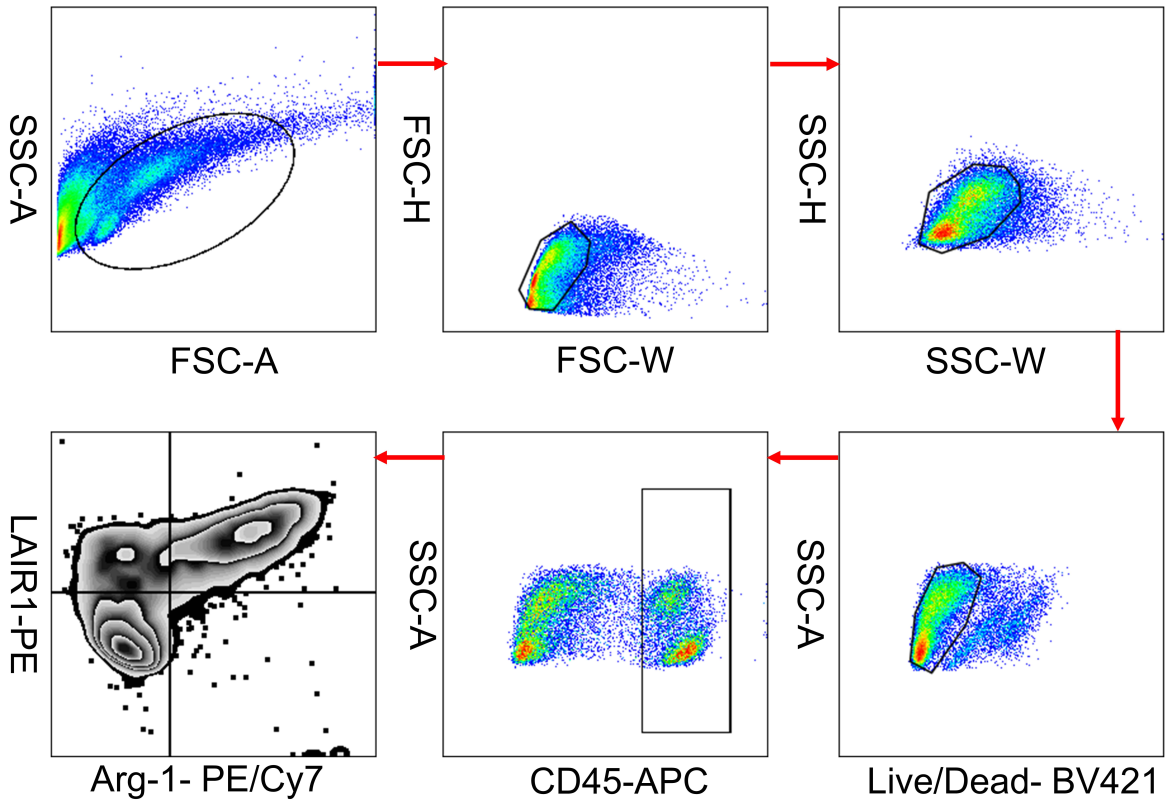

Gating Strategy 2

Gating strategy for Figure 1 H left. Detection of the LAIR1 expression on Arg-1^{+} M2-like TAMs.

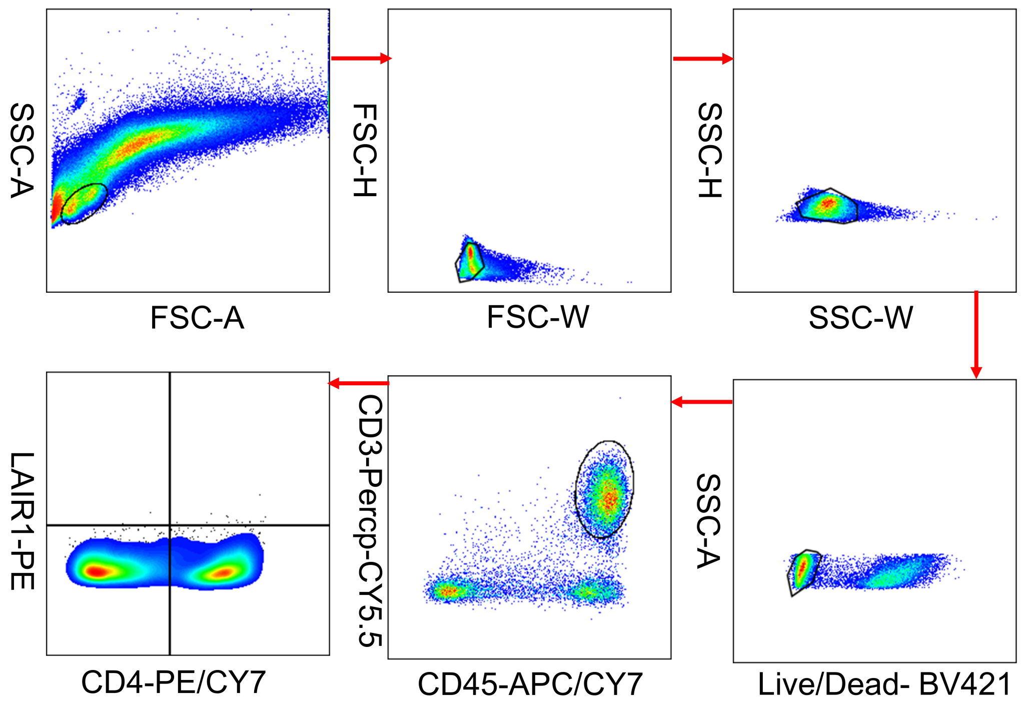

Gating Strategy 3

Gating strategy for Figure 1 H right. Detection of the LAIR1 expression on T cells.

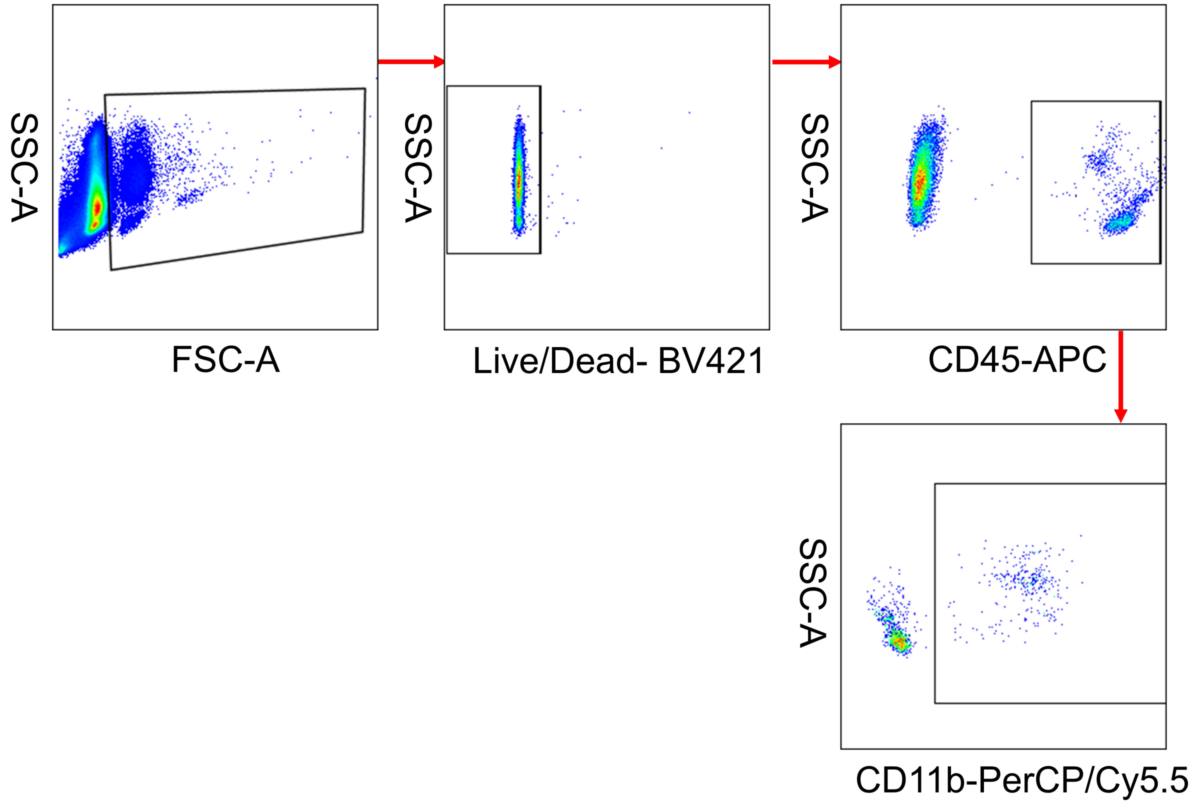

Gating Strategy 4

Gating strategy for Figure 2C. Detection % of $CD45^{+}CD11b^{+}$ myeloid cells in peripheral blood between *Lair1*$^{+/+}$ and *Lair1*$^{-/-}$ mice.

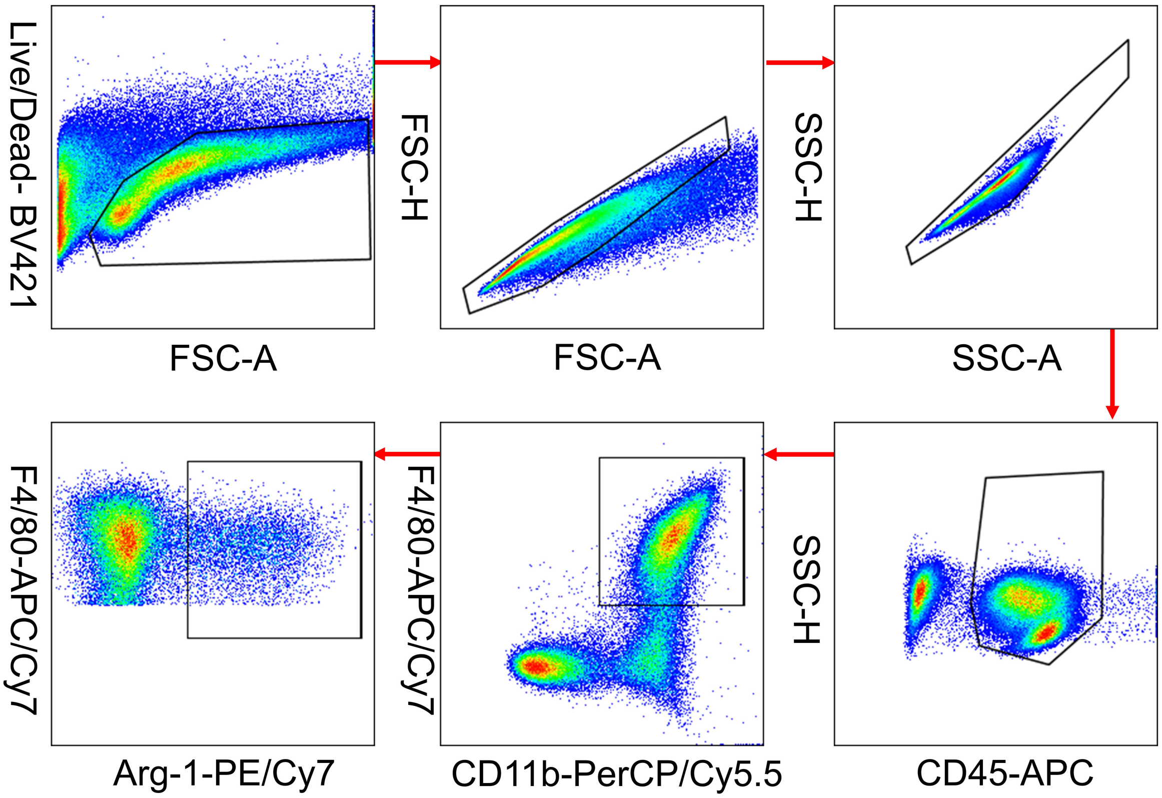

Gating Strategy 5

Gating strategy for Figure 2G and Figure 6F. Detection of MΦs, identified as % of CD45^{+}CD11b^{+} F4/80^{+}, and M2-like TAM, identified as % of CD45^{+}CD11b^{+} F4/80^{+}Arg-1^{+} using tumors from *Lair1*$^{-/-}$ mice.

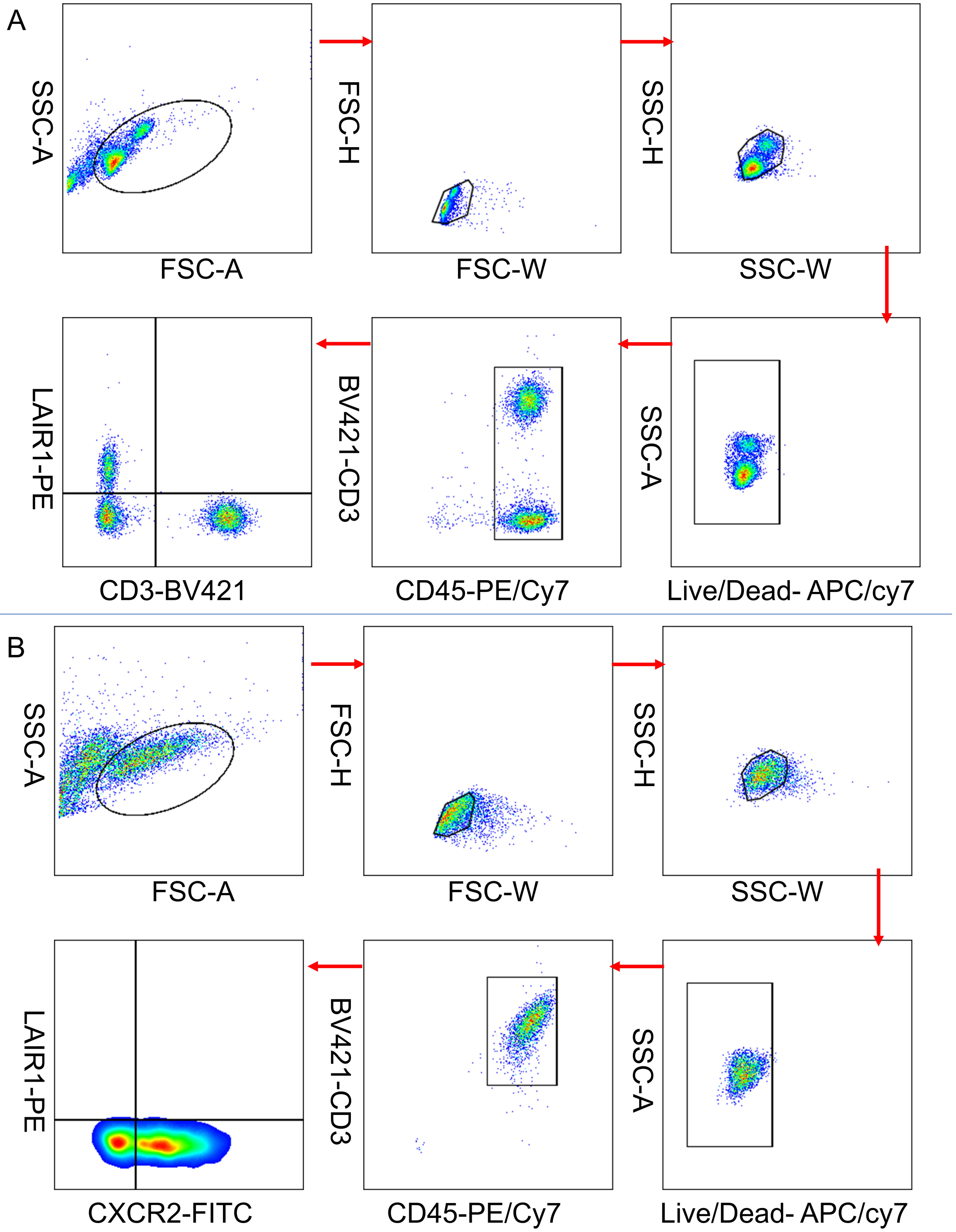

Gating Strategy 6

Gating strategy for Figure 3D. Detection of LAIR1 expression on PBMC (**A**) and 8R-70CAR T cells (**B**).

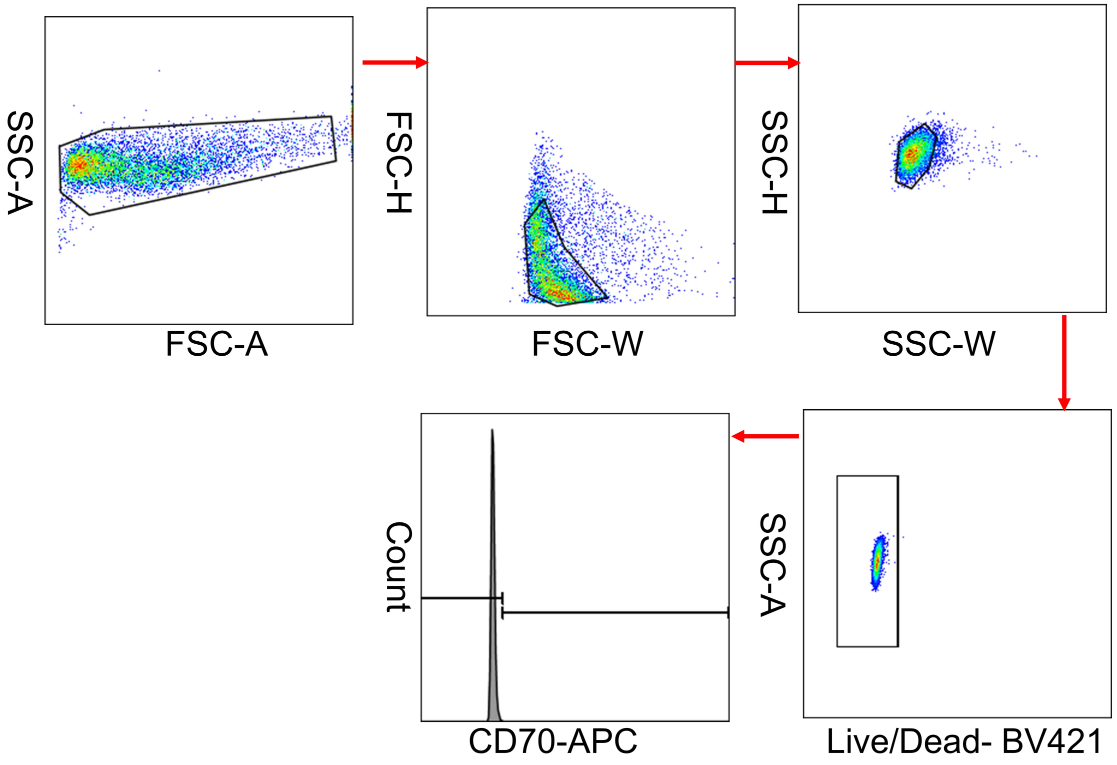

Gating Strategy 7

Gating strategy for Supplemental Figure 3. Detection of CD70 expression on tumor lines

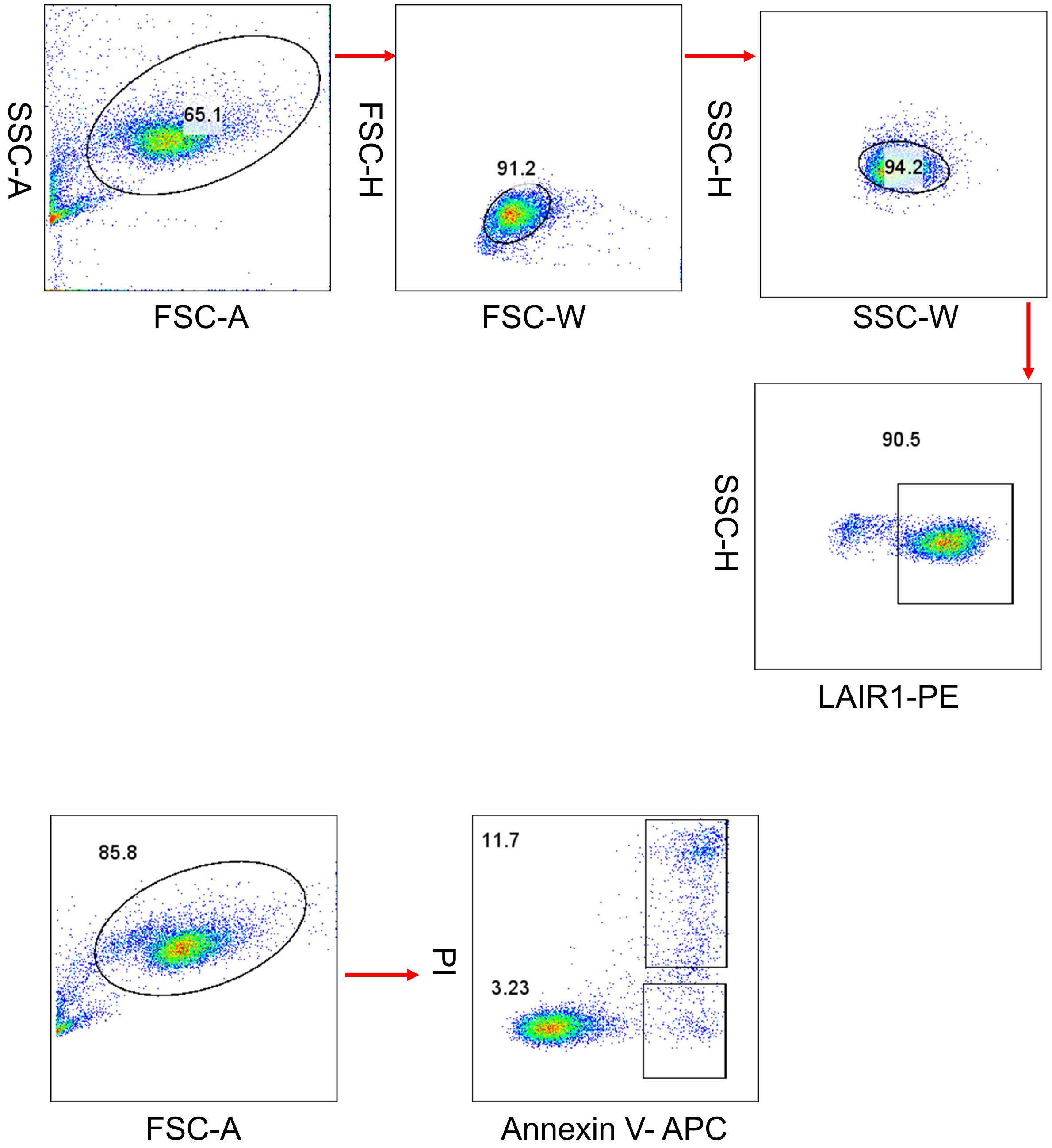

Gating Strategy 8

Gating strategy for Supplemental Figure 14, A and **B.** Detection of LAIR1 expression on Thp-1 and PBMC induced M2-like MΦ (**Upper**), and apoptosis of cells treated by IgG or aLAIR1 (**Downer**).

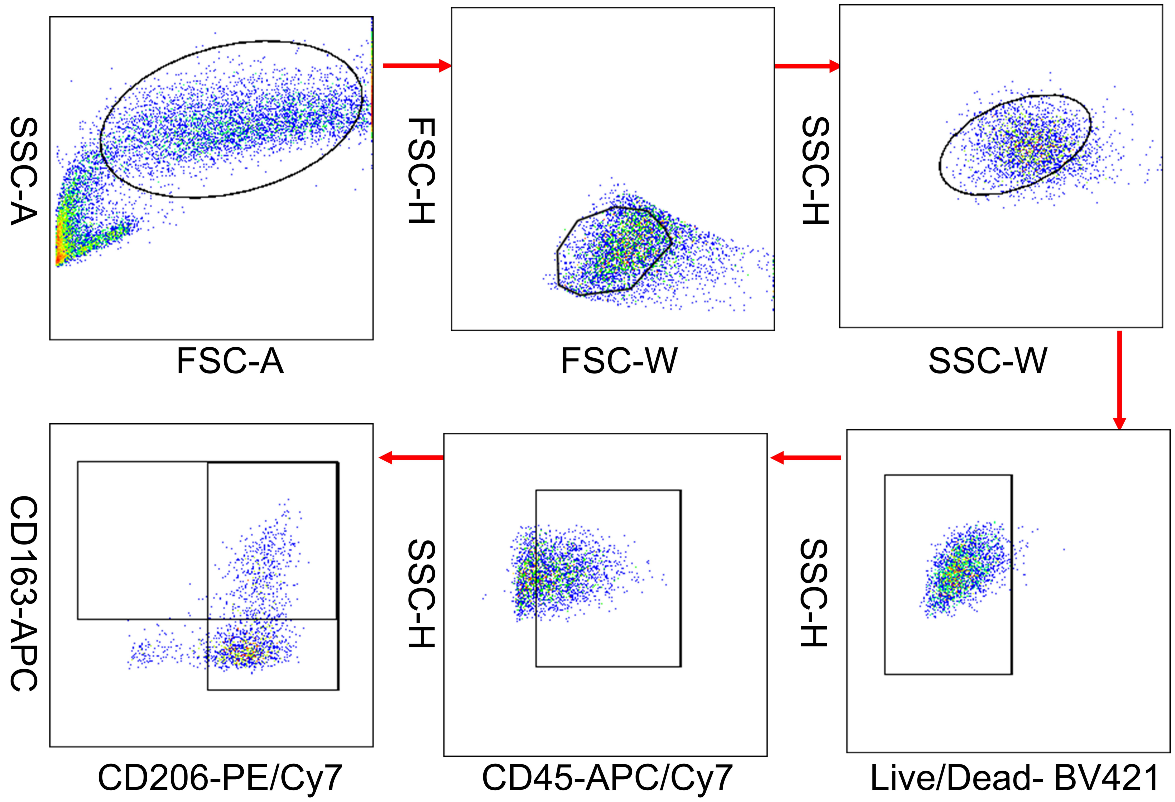

Gating Strategy 9

Gating strategy for Supplemental Figure 14C. Detection of Phenotyping of M2-like MΦ Treated with aLAIR1 or IgG Control

Supplemental Methods

Cell Culture

Murine GBM lines KR158B, KR158B-Luciferase (Luc), KR158B-CD70-Luc, GL261-Luc, or Human GBM line U87 was cultured in Dulbecco's Modified Eagle Medium (DMEM, Thermo Fisher Scientific, 11965092) with 10% Fetal Bovine Serum (FBS, Avantor, 89510) and 1% Penicillin-Streptomycin (Thermo Fisher Scientific,15140163) without sodium pyruvate. The Murine Lewis lung carcinoma line (LLC1) was cultured using Dulbecco's Modified Eagle Medium F12 (DMEM/F12, Thermo Fisher Scientific, 12634028) supplemented with 10% Fetal FBS and 1% Penicillin-Streptomycin. THP-1 line was cultured with RPMI 1640 media with 10% Fetal FBS and 1% Penicillin-Streptomycin. GP2-293 and 293T/17 cells were cultured in DMEM with 10% FBS and 1% Penicillin-Streptomycin containing sodium pyruvate. Human T cells were cultured with AIM-V media (Thermo Fisher Scientific, 087-0112DK) containing 100 IU/ml IL-2 (R&D systems, 202-IL-500) with 5% Human AB serum (Fisher Scientific, HP1022HI)(1). Human Monocytes/MΦs were cultured with AIM-V media with 5% Human AB serum. Cells were kept in 37℃ incubators with 5% CO2.

Cell Isolations

1. Human PBMC and peripheral blood lymphocytes (PBL) isolations**:** PBMC were isolated from Buffy Coats of healthy donors (LifeSouth Community Blood Centers, Gainesville, FL) using density gradient centrifugation method. Briefly, the blood was diluted in PBS followed by Histopaque-1077 (Sigma-Aldrich, H8889-500ML) spin down for 400 x g, 30 min at room temperature. Then, the red blood cells (RBC) were removed by 10X RBC Lysis Buffer (Thermo Fisher Scientific, 00-4300-54), incubating for 3 min in a water bath. The PBL were isolated by plating the PBMC for 2 hours within AIM-V media plus 5% Human AB serum. The non-adherent cells were collected and stored at liquate nitrogen

2. Mouse T cell isolation: Healthy C57/B6 mice spleens were utilized to isolate murine T cells. Splenocytes were obtained by mashing the spleen with a cell strainer (Fisher Scientific, 08-771-2) and removing red blood cells using a 10X RBC Lysis Buffer. Mouse peripheral blood was collected through the fiscal vein, 50-100µl per mouse. The isolation of T cells was carried out using the Pan T kit from Miltenyi Biotec (130-095-130). Subsequently, the T cells were cultured in RPMI 1640 media (11-875-119) supplemented with 30IU/ml human IL-2, 10% FBS, 1% Non-Essential Amino Acids (NEAA, 11-140-050), 1% Sodium Pyruvate (11-360-070), 1% antibiotic-antimycotic (15240062), 0.1% 2-Mercaproethanol (21-985-023), and 1% L-Glutamine (25030-164) sourced from Thermo Fisher Scientific.

3. Mouse tumor single cell suspension: To prepare a single-cell suspension from tumor tissue, we used an enzymatic digestion protocol based on the 'Triple Enzyme Mouse Tumor Digestion' method provided by the NCI. Briefly, the tumor tissues were resected and digested with 1mg/ml Collagenase (C5138), 20 Units/ml DNase (D5025), and 0.1 mg/ml Hyaluronidase (H6254) from Millipore Sigma in gentleMACS™ Octo Dissociator (Miltenyi Biotec) for getting single-cell suspension.

4. Mouse bone marrow isolation

Bone Marrow Isolation Using the Flushing Method. Cut open both ends of the bone to expose the bone marrow; Fill a 10 mL syringe with 10 mL ice-cold RPMI 1640 medium and attach to a 21-gauge needle; Flush out the bone marrow into a centrifuge tube(2). Then, the RBCs were removed by 10X RBC Lysis Buffer, incubating for 3 min in a water bath.

CAR T Cells Transduction

The human or mouse T cells were transduced with either human control vector (pMSGV8, Ctrl), 8R-70CAR, L2-8R-70CAR, or mouse CD70CAR, and the transduction efficiency was determined as described previously (1, 3). Briefly, CD70CAR with different modifications was generated via transient transfection of GP2-293 cells. For retrovirus production, GP2-293 cells were transfected with 2 µg of pMD2.G (Addgene 12259) and 2 µg of described CAR plasmid using 10 µl of Lipofectamine 2000 (Thermo Fisher Scientific, 11668019) in 700 µl of OPTI-MEM (Thermo Fisher Scientific, 31985070). The supernatants were collected 48 hours post-transfection following the CAR transduction procedure.

Human T cells, isolated from PBMCs, were activated 72 hours prior to transduction using anti-CD3/anti-CD28 Dynabeads (Thermo Fisher Scientific, 11161D) at a cell-to-bead ratio of 1:1. Mouse T cells were isolated from splenocytes using the Pan T Cell Isolation Kit, and activated for 48 hours on plates coated with anti-CD3 (Biolegend, 100340, 1 µg/ml) and anti-CD28 (Biolegend, 102116, 2 µg/ml) antibodies. To facilitate transduction, non-tissue culture-treated plates were coated with 20 µg/ml of RetroNectin (Clontech Laboratories, T100A). The CAR T cells were used 72 hours post-transduction.

M2-like MΦ Generation

1. Human $CD14^+$ cells were isolated from human PBMC by $CD14^+$ positive selection beads (Miltenyi Biotec, 130-050-201), then treated with 20 ng/ml M-CSF (PeproTech, AF-300-25) for 5 days, and then cells were then spiked with 20 ng/ml M-CSF, IL-4 (AF-200-04), IL-6 (AF-200-06), IL-13 (AF-200) from PeproTech, and 20 ng/ml IL-10 (R&D Systems, 217-IL-010) for an additional 4 days in AIM-V media. Flow cytometry was performed to assess human M2-like MΦ phenotype ($CD14^+CD80^-CD206^+/CD163^+$)

Mouse Bone Marrow (BM) isolation cells were collected from Lair1$^{+/+}$ and Lair1$^{-/-}$ C57/B6 mice and cultured with mouse M-CSF (PeproTech, 315-02-50) at a concentration of 100 ng/ml for 7 days. The culture medium was supplemented with 20 ng/ml mIL-4 (214-14), 20 ng/ml mIL-10 (210-10), 20 ng/ml mIL-13 (210-13) from PeproTech(4)in collagen I (5µg/ml, Millipore Sigma, CC050) and Collagen IV (5µg/ml, Millipore Sigma, CC076) coated plate for 48 hours. Western blotting was used to determine FXIII-A expression between *Lair1*$^{+/+}$ and *Lair1*$^{-/-}$ M2-like MΦ.

FXIII-A Experiments

BM-derived M2-like MΦ (2x10^5/ml) were cultured with 10 µg/ml FXIII-A_2 protein (Zedira GmbH, T061) in Collagen I and Collagen IV (total 10µg/ml) coated plate for 48 hours. Flow cytometry was performed to assess human M2-like MΦ phenotype (CD45$^+$CD11b$^-$F4/80$^+$Arg-1$^+$). KR158B-CD70-Luc cells (1x10^5/ml) were cultured with 10 µg/ml FXIII-A2 protein in Collagen I and Collagen IV (total 10µg/ml) coated plate for 24 hours, IF was performed to assess collagen IV expression.

In Vivo Imaging System (IVIS)

The process of IVIS was determined as described previously (1, 3). Briefly, Isoflurane-anesthetized animals were imaged using the IVIS system (Xenogen, Alameda, CA). The luminescent signal of Luciferase was detected 5 min after 50µl d-luciferin (PerkinElmer) injection intraperitoneally. The average radiant efficiency ([p/s/cm^2/sr]/[µW/cm^2]) within the ROI was used for quantitative fluorescence analysis. The average radiance (p/s/cm^2/sr) within the ROI was used for all quantitative luminescence analyses.

Tumor-bearing (TB) Mice and Treatment

TB mice were grouped based on IVIS measurements taken 1 day before treatment (-day 1, or -D1) to ensure similar initial tumor sizes. The average luminance ranged from 1×10^4 to 1×10^5, with comparable mean luminance across groups. aLAIR1 (R&D Systems, MAB100921) or IgG control

(R&D Systems, AB-105-C), the detailed information described in Functional antibodies, **Supplemental Table 1**. The mouse aLAIR1/IgG was administered via Intraperitoneal (i.p.) injection at a dose of 200 µg every other day from day 0 to day 8, 5 doses total. For the combination therapy, TB mice received 5 Gy total body irradiation on -day 1, followed by an intravenous (i.v.) injection of 1×10^7 CD70CAR T cells on day 0, with aLAIR1 administered at 200 µg/dose every other day from day 1 to day 9, 5 doses in total. For the L2-8R-70CAR and 8R-70CAR treatments, TB mice received 5 Gy total body irradiation on -day 1, followed by two intravenous i.v. injections of 1×10^7 CAR T cells on days 0 and 3. The tumor increases were calculated based on the baseline (Luminance -D1) of each mouse. e.g., The tumor increases on day 28 were calculated using the formula: (Luminance D28 - Luminance -D1)/ Luminance -D1 x 100%. The survival of TB mice was monitored per veterinary recommendations, and partial blinding was implemented in the study. Across all mouse models (KR158B-Luc, GL261-Luc, LLC1, and KR158B-CD70-Luc), no significant changes in body weight were observed during the initial two weeks (or four weeks for the KR158B-CD70-Luc model) following treatment.

Timepoints For Tissue Collection from Mice

Aside from the endpoint of each TB mouse, tumor tissues were collected based on measurements obtained through IVIS imaging for brain tumors or calipers for LLC1 tumors, following the observation of noticeable trends of differences among the groups. Specifically, tumor tissues from KR158B-Luc TB mice were collected within 32±7 days, from KR158B-CD70-Luc mice within 42±7 days, and from LLC1 tumors within 12±3 days.

3D In Vitro Immunotherapy Assay

The 3D in vitro immunotherapy assay (iVITA) for evaluating cancer-immune interactions has been previously described (5). Briefly, tumor spheroids were generated by homogeneously mixing suspended cancer cells (4×10^3 cells/well) with inert liquid-like solid (LLS) microgels and culturing

them in a Darcy plate under continuous perfusion (6). Tumor spheroids began forming within 48 hours and matured by day 7. To facilitate cell adhesion and migration, we employed collagen type I (COL1-LLS) bio-conjugated microgels using EDC-NHS chemistry(5-7). A defined ratio (5:1) of M2-like MΦ to CAR or NAS T ($8x10^3$:$1.6x10^3$/well) cells was uniformly distributed around the tumor by homogenizing the cells at a selected concentration in COL1-LLS and depositing the mixture into a glass-bottom 96-well plate. Next, a custom-built Nikon Stage Mount 3D bioprinting micromanipulator (BioPelle™; Aurita Bioscience, BPLE_NKN_FC) was used to precisely position the solid tumor models within the working distance of the Nikon A1R confocal objective (20 X /0.75, Nikon Plan Apo VC). The plate was then centrifuged at 100xg with gentle acceleration and deceleration settings. Following centrifugation, growth media was carefully added, and the plate was secured in a custom-made stage incubator (Microscope Incubator, Aurita Bioscience, INC_NKN_STD) on a Nikon A1R confocal microscope equipped with a high-definition Galvano scanner for long-term in situ imaging. The co-cultures were maintained in incubators at 37°C and 5% CO_2. Using the iVITA platform, we successfully captured and quantified key T cell functions, including trafficking, T cell/M2-like MΦ interactions, T cell/tumor interaction or infiltration, and tumor area analysis (5). For tracking and quantification, the entire confocal z-stack (150 μm range, step size: 2.4 μm) was reviewed to ensure uniform 3D distribution of T cells and M2-like MΦ surrounding the tumor at each 1-hour imaging interval. Maximum intensity projections of the time lapse were created, and individual channels were analyzed using FIJI (version 2.9.0/1.53t). The channels included red (excitation: 561 nm, emission: 570-616 nm) for T cells labeled with CellTracker™ Orange CMRA (Thermo Fisher Scientific, C34551), green (excitation: 488 nm, emission: 500-550 nm) for M2-like MΦ (labeled with CellTracker™ Green CMFDA, Thermo Fisher Scientific, C7025), and tumors cells were not stained with a fluorescent reporter. Cell segmentation was performed using intensity thresholds to ensure at least 95% overlap between thresholded objects and cell signals. Small particles (0-20 μm^2 in the T-cell channel and 0-50 μm^2 in the M2-like MΦ and tumor channels) were excluded as noise. The percentage of T and M2-like

MΦ; CAR T and tumor interactions was calculated by normalizing the intersected areas (M2-like MΦ vs. T cells, and T cells vs. tumors) to the total T cell area at each 1-hour interval. T cell migration was tracked using the FIJI TrackMate Linear Assignment Problem (LAP) tracker, with a maximum frame-to-frame linking distance and an allowable track segment gap closure of 150 µm.

Chipcytometry

Chipcytometry procedure was performed by Canopy Biosciences (Hannover, Germany). Tissue cryo-sections were prepared on a cryostat (NX50) with a thickness of 7 µm and stored overnight at -80°C before fixation in ice-cold acetone for 5 min, followed by 3 min of 90% and 70% EtOH, respectively. Fixed tissue sections were rinsed in PBS for 6 min and then loaded onto tissue chips. The chips were filled with storage buffer and stored at 4°C. Chips were rinsed with washing buffer for the staining procedure, and tissue autofluorescence was imaged in the FS560 channel. The autofluorescence images were used to assess tissue structure and integrity and to select regions of interest.

The Chipcytometry assay consists of iterative staining, imaging, and photo-bleaching cycles. The three steps are repeated until all desired markers have been stained and imaged. After initial bleach and background imaging, antibodies were diluted from their stock solution in storage buffer to a total volume of 600 µl and briefly mixed according to the stain plan shown in the legend of **Supplemental Figure 5**. The antibody working solution was then pipetted dropwise into the liquid adapter of the chip, taking care not to introduce air bubbles into the chip. The chip was incubated for 15 min at room temperature before the working solution was flushed by pipetting 1 mL of storage buffer into the chip and then washed with 3 x 5 mL wash buffer with 2-5 min pause between wash steps. After washing, the chips were immediately put on the ZellScanner ONE instrument (Canopy Biosciences) and imaged, completing the cycle. This procedure followed the

stain plan outlined in the legend of **Supplemental Figure 5** until all markers were stained. Image data were processed and analyzed using the ZellExplorer Application (Canopy Biosciences).

Cytokine Production

To assess the cytokine release in the 3D culture, the culture supernatants were harvested and assayed 72 hours later by the multiplexing analysis performed by Eve Technologies Corp. (Calgary, Alberta) on the Luminex™ 200 system (Luminex). Briefly, markers were simultaneously measured in the samples using Human Cytokine 48-Plex Discovery Assay® manufactured by Millipore Sigma. Individual analyte sensitivity values are available in the MILLIPLEX® MAP protocol. Individual ELISA was performed in-house using kits purchased from Thermo Fisher Scientific to confirm the results obtained above (G-CSF: BMS2001INST and CCL3: 88-7035-22). To evaluate the functionality of aLAIR1 in counteracting M2-like MΦ-mediated T-cell suppression in vitro using a 2D culture system. A flat-bottom 96-well plate was coated overnight at 4 °C with a collagen I/IV (10 µg/ml). The plate was then washed and air-dried at room temperature. 8R-CD70CAR T cells and M2-like MΦ were derived from the same human blood donor. 8R-CD70CAR T cells were co-cultured with M2-like MΦ and U87 GBM line at a ratio of M2-like MΦ: 8R-70CAR T: Tumor=5:1:2.5 ($2x10^5$:$4x10^4$: $1x10^5$) overnight, in the presence or absence of IgG or aLAIR1 (5 µg/ml) IFN-γ assays were performed by ELISA (Thermo Fisher Scientific, KHC4021). The LAIR2 release from L2-8R-70CAR T cells at multiple time points was determined by ELISA (R&D Systems, DY2665). To evaluate the anti-tumor effect of L2-8R-70CAR and 8R-70CAR T cells in human, the CAR T cells were co-cultured overnight with U87/pGBM#1 at a ratio of 1:1 ($1x10^5$: $1x10^5$). IFN-γ assays were performed by ELISA. To evaluate the anti-tumor effect of L2-8R-70CAR and 8R-70 CAR T cells in mice, the CAR T cells were co-cultured overnight with KR158B-CD70-Luc/KR158B-Luc at a ratio of 1:1 ($1x10^5$: $1x10^5$). IFN-γ assays were performed by ELISA (Thermo Fisher Scientific, BMS606).

Antibody Binding Evaluation

Functional ELISA was performed to evaluate whether Human aLAIR1 inhibits LAIR1–collagen interaction. Firstly, increasing concentrations (0.002, 0.005, 0.009, 0.19, 0.38, 0.75, 1.5, 3, 6, 12, 24, 48, 96, 192μg/ml) of human LAIR1-His tag protein (R&D systems, 2664-LR) were incubated on collagen I/IV–coated plates (10μg/ml) for 2 hours at room temperature. Binding was quantified by His-tag ELISA, a reagent from R&D systems (BAM050, DY994, DY998, DY999), and EC_{50} was identified. Then 1μg/ml (approximately value of EC_{50}) LAIR1-His protein was pre-incubated with increasing concentrations (0.013, 0.025, 0.05, 0.1, 0.2, 0.4, 0.8, 1.59, 3.18, 6.25, 12.5, 25, 50, 100, 200, 400μg/ml) of human aLAIR1(Hycult biotech, HM2364) or IgG (Hycult biotech, HI1016) for 1 hour, then added to uncoated plates for 2 hours. Three collagen-coated wells served as maximal (Max, 100%) binding controls. Binding was quantified by His-tag ELISA and reported as % response relative (% to Max) to the LAIR1-only collagen-coated control. Finally, aLAIR1 or IgG was pre-incubated with LAIR1-His, as described above, followed by incubation on collagen I/IV-coated plates for 2 hours at room temperature. Binding was quantified by His-tag ELISA. The inhibition was calculated as 100% minus the relative response (%), i.e., Inhibition = 100% – % response relative. The IC_{50} value was calculated by nonlinear regression using a four-parameter logistic model.

Flow Cytometry

For fluorescent conjugated antibodies surface staining, cells were stained with various markers at room temperature for 15 min, washed with PBS, and resuspended in PBS containing 1:1000 dilution of LIVE/DEAD™ Fixable Dead Cell Stain Kit (Thermo Fisher Scientific, C34557). Washed once and resuspended in FACS buffer (PBS with 2% fetal calf serum) before analysis on a flow cytometer. For intracellular/intranuclear staining, such as Arg-1, cells were first labeled with LIVE/DEAD™ Fixable Dead Cell Stain Kit as described above. Cells were then fixed/permeabilized using the Foxp3 Transcription Factor Staining Buffer Set (Thermo Fisher

Scientific, 00-5523-00) for 1 hour at 4 °C and were stained with the surface maker and Arg-1 antibodies (Thermo Fisher Scientific, 25-3697-82). To evaluate the Gramz B and TNF-α in T cells, the cells were cultured with GolgiStop (BD, 554724, 0.67µl/ml) in 37 °C incubator for 6 hours, followed by fixed/permeabilized using the Foxp3 Transcription Factor Staining Buffer Set for 1 hour at 4 °C and were stained with the surface maker and intracellular antibodies. Detailed information on antibodies is presented in **Supplemental Table 1**. All the samples were analyzed using Canto II™ Flow Cytometer, and acquired data were analyzed using FlowJo software version 10.8.1. Gating strategies are displayed in above.

T-cell Proliferation Assay

T-cell proliferation was evaluated using flow cytometry following 3 days of stimulation with anti-CD3/anti-CD28 Dynabeads (Thermo Fisher Scientific). A flat-bottom 96-well plate was coated overnight at 4 °C with a collagen I & IV mixture (Sigma, total 10 µg/ml). The plate was then washed and air-dried at room temperature. T cells were stained with CellTrace Violet (Thermo Fisher Scientific, C34557) and co-cultured with M2-like MΦ derived from the same donor's PBMCs. The cells were added to each well at a 5:1 ratio (2 × 10^5 M2-like MΦ: 4 × 10^4 T cells per well). Anti-CD3/anti-CD28 Dynabeads (Thermo Fisher Scientific) were included at a 1:1 T-cell-to-bead ratio, with 4 × 10^4 beads per well. Daily treatments included aLAIR1, IgG control (Hycult Biotech, 5 µg/ml), LAIR1 agonist (8) (BD, 550810, 5 µg/ml), or PBS (Thermo Fisher Scientific, 10 µl). After 3 days of culture, T-cell proliferation was assessed by Flow Cytometry (gating on $CD3^+Celltrace^-$).

Cell Apoptosis Elevation

To elevate the function of haLAIR1 on $LAIR1^+$ cells, the THP-1 cells and PBMC-induced M2-like MΦ ($1x10^5$/ml) were cultured with 5 µg/ml of IgG or aLAIR1 for 3 days in a collagen I/IV coated plate. Flow cytometry assessed these cells' viability using Annexin V (Biolegend, 640941) and

Propidium Iodide (PI, Thermo Fisher Scientific, P1304MP). Annexin V^+PI^- cells represent early apoptosis cells, and Annexin V^+PI^+ cells are late apoptosis cells.

In-house Single-Cell RNA Sequencing and Data Analysis

Tumor single-cell suspensions were first washed with PBS, and their viability was confirmed through Trypan Blue staining. These suspensions were subsequently labeled and combined using the 3' CellPlex Kit Set A. Following this, they were loaded onto the Chromium Single Cell Chip G (10x Genomics) as per the manufacturer's guidelines, aiming for a target capture rate of approximately 30,000 cells per sample. The pooled single-cell RNA-seq libraries were then prepared according to the manufacturer's instructions, utilizing the Chromium Single Cell 3′ V3.1 Solution (10x Genomics). All single-cell cDNA was divided into two types of libraries: 3' Gene Expression libraries and Cell Multiplexing libraries. Both library types were subjected to paired-end, dual indexing sequencing (SI-TT for gene expression and SI-NN for multiplexing) on the Illumina Novaseq platform. This sequencing process included a 28-base read 1 containing cell barcodes and unique molecular identifiers (UMI) and a 150-base read 2 for mRNA insert. The sequencing depth aimed to achieve more than 20,000 read pairs per cell for gene expression and 5,000 read pairs per cell for multiplexing. The data were analyzed by the Department of Neurosurgery at the University of Florida using the same analysis method. The demultiplexed cells were aligned to the mouse mm10 genome using Cellranger 7.0 (10x genomics). Subsequently, the data was analyzed using Seurat 4.0 (9). The analysis began with quality control metrics, where cells were assessed based on mRNA count (>250), barcodes per cell (>500), cell complexity (log10GenesPerUMI > 0.80), and percentage of mitochondrial genes (<20%). SingleR v.2.4.1 was employed for cell cluster deconvolution, utilizing cell type signature genes obtained from the differentially expressed genes (DEGs) within the clusters (10). Moreover, CellChat 2.1.0 was utilized to explore cell-to-cell interactions (11). To identify differentially expressed genes

(DEGs) on specific cell types between groups, the Limma-voom 3.58.1 method was employed (12). Subsequently, ggplot2 3.4.1 was used to generate relevant plots (13).

Western Blotting

The BM and M2-like MΦ cells were treated with a RIPA buffer (Thermo Fisher Scientific, 89900) containing a cocktail of Protease and Phosphatase Inhibitor Cocktail (Thermo Fisher Scientific, 78440) and placed on ice for 20 min and then centrifuged the samples at 4℃ at 13,000 x g for 15 min. The lysate supernatant was collected, and the protein concentration was determined by Pierce™ BCA Protein Assay Kits (Thermo Fisher Scientific, 23225). The same amount of proteins was analyzed by SDS-PAGE (Bio-Red) and transferred to polyvinylidene fluoride (PVDF) membranes (Thermo Fisher Scientific, 88518). The membrane was blocked in TBST (Thermo Fisher Scientific, J77500.K2) with Superblock buffer (Thermo Fisher Scientific, 37515) for 1 h and then kept at 4℃ overnight with anti-FXIII-A1 primary antibody (Santa Cruz Biotechnology, sc-271122, 1:100). Detailed information of antibodies is presented in **Supplemental Table 1**. The membrane was then washed with TBST at least 3 times and incubated at room temperature with goat-anti-rabbit IgG HRP (R&D systems, HAF09, 1:500) for 1 hour before exposure and photography.

Immunofluorescence

Immunofluorescence (IF) was performed for CD45, Tmem119, LAIR1, EGFR, collagen I, collagen IV expression, CD8, and CXCR2. Tumor brain samples from humans or mice were freshly embedded in OCT, slowly frozen in a slush of dry ice and 95% alcohol, and then cryosectioned (5 or 10 µm). Sections were incubated with Superblock buffer with 0.2% Triton X-100 (Thermo Fisher Scientific, A16046.AE) for 1 hour before staining and stained with primary antibodies: rabbit anti-human LAIR1 (Cell Signaling, 60061, 1:100); rat anti-human CD45 (Thermo Fisher Scientific,

MA5-17687, 1:100); mouse anti-human EGFR (Thermo Fisher Scientific, MA5-13070, 1:200); mouse anti-human Tmem119 (Cell Signaling, 41134, 1:50), Rabbit anti-mouse collagen I (R&D Systems, NB600-408, 1:100), rabbit anti-mouse collagen IV (Abcam, ab19808, 1:100), Rabbit anti-human CXCR2 (Gene Tex, GTX639056, 1:50), Rat anti-mouse CD8 (Thermo Fisher Scientific, MA1-145, 1:100) at 4 °C for overnight, followed by secondary antibodies coupled with a fluorophore (goat anti-rabbit IgG-Alexa Fluor488, and goat anti-rat IgG- Fluor568, Goat anti-mouse IgG-Alexa Fluor647, Thermo Fisher Scientific, 1:500) at room temperature for 1 h. Detailed information on antibodies is presented in **Supplemental Table 1.** DAPI (Thermo Fisher Scientific, D1306) was used for nuclei staining. Additional sections were prepared and stained with flour-conjugated secondary antibodies only to confirm the specificity of the primary antibodies for their indicated epitopes. Images were acquired using a Nikon A1RMP Confocal Microscope, using the following visible excitation lasers (405nm, 488nm, 561nm, and 647nm) and emission filter bands (450/50, 525/50, 595/50, 700/75). Mono-LASER based segregated stepwise image acquiring algorithm set up as default from LASERs 405nm, 488nm, 561nm, and 647nm to minimize spectral spillover between channels. For imaging quantitation, density analysis was performed with Fiji/ImageJ Software (14). Voxel-based co-localization as well as 3D reconstruction of the confocal images was performed using the Imaris Cell Imaging Software x 64 9.7.0 (Oxford Instruments). For imaging cell number analysis was performed using Qupath-0.5.1 software. To determine if LAIR1 is on tumor-infiltrating MΦ or resident microglia, $CD45^+$ cells were divided into $CD45^{high}$ (top 50%) and $CD45^{low}$ (bottom 50%) populations. The Collagen I & IV levels (measured as mean fluorescence intensity, MFI) were calculated by the formula: (Total intensity – Background intensity)/ Total nuclei. The % of CAR T cell infiltration was calculated by the formula: Total mouse $CD8^+$ and human $CXCR2^+$ double-positive cells/ Total nuclei.

Immunohistochemistry (IHC) of Cancer Tissues

The IHC images of tumor tissues were acquired from the Human Protein Atlas, version 23.0 (https://www. proteinatlas.org/) (15). The original images can be found:

Colorectal Cancer: Patient ID: 693

https://www.proteinatlas.org/ENSG00000167613-LAIR1/cancer/colorectal+cancer#

Lung Cancer: Patient ID: 2222

https://www.proteinatlas.org/ENSG00000167613-LAIR1/cancer/lung+cancer#img

Melanoma: Patient ID: 2534

https://www.proteinatlas.org/ENSG00000167613-LAIR1/cancer/melanoma#img

Ovarian Cancer: Patient ID: 2391

https://www.proteinatlas.org/ENSG00000167613-LAIR1/cancer/ovarian+cancer#img

Renal Carcinoma: Patient ID: 1481

https://www.proteinatlas.org/ENSG00000167613-LAIR1/cancer/renal+cancer#img

Stomach Cancer: Patient ID: 2142

https://www.proteinatlas.org/ENSG00000167613-LAIR1/cancer/stomach+cancer#img

Testis Cancer: Patient ID: 1777

https://www.proteinatlas.org/ENSG00000167613-LAIR1/cancer/testis+cancer#img

Lymphoma: Patient ID: 1734

https://www.proteinatlas.org/ENSG00000167613-LAIR1/cancer/lymphoma#img

Supplemental Table 1. Antibodies used in this study

ANTIBODIES	PROVIDER	IDENTIFIER	APPLICATION
**Functional antibodies**			
Mouse anti-human LAIR1	Hycult biotech	Cat# HM2364<br>Clone: NKTA255<br>RRID:100ugAB_2942052	LAIR1 Blockade
Rabbit anti-mouse LAIR1	R&D Systems	Cat# MAB100921<br>Clone: 2459B<br>RRID: AB_3657110	LAIR1 Blockade
Rat anti-mouse PD-1	BioXCell	Cat# BE0146<br>Clone: RMP1-14<br>RRID: AB_10949053	PD-1 Blockade
Mouse IgG	Hycult biotech	Cat# HI1016-50ug<br>Clone: MOPC-21<br>RRID: AB_2942051	Control
Rabbit IgG	R&D Systems	Cat# AB-105-C<br>Clone: 2459B<br>RRID: AB_354266	Control
Mouse anti-human LAIR1 (LAIR1 agonist)	BD	Cat# 550810<br>Clone: DX26<br>RRID: AB_393895	LAIR1 agonist
Ultra-LEAF™ Purified Anti-mouse CD3ε	Biolegend	Cat# 100340<br>Clone: 145-2C11<br>RRID: AB_11149115	T cell stimulation
Ultra-LEAF™ Purified Anti-mouse CD28	Biolegend	Cat# 102116<br>Clone: 37.51<br>RRID: AB_11147170	T cell stimulation
**Detecting antibodies**			
Rat anti-human CD45	Thermo Fisher Scientific	Cat# MA5-17687;<br>Clone: YAML501.4<br>RRID: AB_2539077	IF (1:100)
Rabbit anti-human LAIR1	Cell Signaling	Cat# 60061<br>Clone: E7X6I<br>RRID: AB_2238514	IF (1:100)
Mouse anti-Human Tmem119	Cell Signaling	Cat # 41134<br>Clone: E3E4T<br>RRID: AB_3094467	IF (1:50)
Mouse anti-human EGFR	Thermo Fisher Scientific	Cat# **MA5-13070**<br>Clone: H11<br>RRID: AB_10977527	IF (1:200)
Anti-mouse CD8	Thermo Fisher Scientific	Cat# 11-0081-82<br>Clone: 53-6.7<br>RRID: AB_464915	IF (1:100)
Rabbit anti-mouse Collagen IV	Abcam	Cat# ab19808<br>Clone: COL4A1<br>RRID: AB_445160	IF (1:100)
Rabbit anti-human CXCR2	Gene Tex	Cat# GTX639056<br>Clone: HL2604	IF (1:50)

Rat anti-mouse CD8	Thermo Fisher Scientific	Cat# MA1-145<br>Clone: 2.43<br>RRID: AB_2536854	IF (1:100)
PerCP/Cyanine5.5 hamster anti-mouse CD3	BD	Cat# 551163<br>Clone: 145-2C11<br>RRID: AB_394082	FC (1:100)
APC rat anti-mouse CD45	BD	Cat# 559864<br>Clone: 30-F11<br>RRID: AB_398672	FC (1:100)
PE/Cyanine7 rat anti-mouse CD45	Biolegend	Cat# 103114<br>Clone: 30-F11<br>RRID: AB_312979	FC (1:100)
APC rat anti-mouse CD45	BD	Cat# 561018<br>Clone: 30-F11<br>RRID: AB_398672	FC (1:100)
APC/Cyanine7 rat anti-mouse CD45	BD	Cat# 557659<br>Clone: 30-F11<br>RRID: AB_396774	FC (1:100)
PerCP/Cyanine5.5 rat anti-mouse CD11b	BD	Cat# 561114<br>Clone: M1/70<br>RRID: AB_2033995	FC (1:100)
APC/Cyanine7 anti-mouse F4/80	Biolegend	Cat# 123118<br>Clone: BM8<br>RRID: AB_893489	FC (1:100)
PE Armenian hamster anti-mouse LAIR1	Thermo Fisher Scientific	Cat# 12-3051-82<br>Clone: 113<br>RRID: AB_1210738	FC (1:100)
PE-Cyanine7 rat anti-mouse Arg-1	Thermo Fisher Scientific	Cat# 25-3697-82<br>Clone: A1exF5<br>RRID: AB_2734841	FC (1:100)
PE-Cyanine7 rat anti-mouse CD4	BD	Cat# 552775<br>Clone: RM4-5<br>RRID: AB_394461	FC (1:100)
anti-mouse CD70	BD	Cat# 555286<br>Clone: FR70<br>RRID: AB_395705	FC (1:100)
APC/Cyanine7 anti-human CD14	Biolegend	Cat# 301820<br>Clone: M5E2<br>RRID: AB_493695	FC (1:100)
PE/Cyanine7 anti-human CD206	Biolegend	Cat# 321124<br>Clone: 15-2<br>RRID: AB_10933248	FC (1:100)
APC anti-human CD163	Biolegned	Cat# 333610<br>Clone: GHI/61<br>RRID: AB_2291272	FC (1:100)
PE-Cyanine7 moue anti-human CD45	Biolegned	Cat# 368532<br>Clone: 2D1<br>RRID: AB_2715892	FC (1:100)

BV421 mouse anti-human CD3	BD	Cat# 563798<br>Clone: SK7<br>RRID: AB_2744383	FC (1:100)
APC mouse anti-human CD70	Biolegned	Cat# 355110<br>Clone: 113-16<br>RRID: AB_2562481	FC (1:100)
FITC mouse anti-human CXCR2	Biolegend	Cat# 320704<br>Clone: 5E8/CXCR2<br>RRID: AB_439805	FC (1:100)
PE Mouse anti-human LAIR1	Thermo Fisher Scientific	Cat# 12-3059-42<br>Clone: NKTA255<br>RRID: AB_11042284	FC (1:100)
PE mouse anti-Human Granzyme B	BD	Cat# 561142<br>Clone: GB11<br>RRID: AB_10561690	FC (1:100)
PE-Cyanine7 moue anti-human TNF-a	Biolegend	Cat# 502930<br>Clone: MAb11<br>RRID: AB_2204079	FC (1:100)
Mouse anti FXIII-A	Santa Cruz Biotechnology	Cat# sc-271122<br>Clone: A-4<br>RRID: AB_10608225	WB (1:100)
Goat anti-rabbit IgG HRP	R&D Systems	Cat# HAF008<br>RRID: AB_357235	WB (1:500)
Goat anti-rabbit Alexa Fluor 488	Thermo Fisher Scientific	Cat# A-11034<br>RRID: AB_2576217	IF (1:500)
Goat anti-rabbit Alexa Fluor 568	Thermo Fisher Scientific	Cat# A-11011<br>RRID: AB_143157	IF (1:500)
Goat anti-rat Alexa Fluor 568	Thermo Fisher Scientific	Cat# A-11077<br>RRID: AB_2534121	IF (1:500)
Goat anti-mouse Alexa Fluor 647	Thermo Fisher Scientific	Cat# A-21235<br>RRID: AB_2535804	IF (1:500)
Goat anti-Hamster Alexa Fluor 674	Thermo Fisher Scientific	Cat# A-21451<br>RRID: AB_2535868	IF (1:500)

Note: IF: Immunofluorescence, FC: Flow Cytometry, WB: Western Blotting

Reference

1. Jin L, Tao H, Karachi A, Long Y, Hou AY, Na M, et al. CXCR1- or CXCR2-modified CAR T cells co-opt IL-8 for maximal antitumor efficacy in solid tumors. *Nature Communications.* 2019;10(1):4016.
2. Song R, Bafit M, Tullett KM, Tan PS, Lahoud MH, O'Keeffe M, et al. A Simple and Rapid Protocol for the Isolation of Murine Bone Marrow Suitable for the Differentiation of Dendritic Cells. *Methods and Protocols.* 2024;7(2):20.
3. Jin L, Ge H, Long Y, Yang C, Chang YE, Mu L, et al. CD70, a novel target of CAR T-cell therapy for gliomas. *Neuro Oncol.* 2018;20(1):55-65.
4. Zarif JC, Hernandez JR, Verdone JE, Campbell SP, Drake CG, and Pienta KJ. A phased strategy to differentiate human CD14+monocytes into classically and alternatively activated macrophages and dendritic cells. *Biotechniques.* 2016;61(1):33-41.

5. Nguyen DT, Liu R, Ogando-Rivas E, Pepe A, Pedro D, Qdaisat S, et al. Bioconjugated liquid-like solid enhances characterization of solid tumor - chimeric antigen receptor T cell interactions. *Acta Biomater.* 2023;172:466-79.
6. Nguyen DT, Famiglietti JE, Smolchek RA, Dupee Z, Diodati N, Pedro DI, et al. 3D In Vitro Platform for Cell and Explant Culture in Liquid-like Solids. *Cells.* 2022;11(6).
7. Nguyen DT, Pedro DI, Pepe A, Rosa JG, Bowman JI, Trachsel L, et al. Bioconjugation of COL1 protein on liquid-like solid surfaces to study tumor invasion dynamics. *Biointerphases.* 2023;18(2):021001.
8. Meyaard L, Adema GJ, Chang C, Woollatt E, Sutherland GR, Lanier LL, et al. LAIR-1, a Novel Inhibitory Receptor Expressed on Human Mononuclear Leukocytes. *Immunity.* 1997;7(2):283-90.
9. Hao Y, Hao S, Andersen-Nissen E, Mauck WM, Zheng S, Butler A, et al. Integrated analysis of multimodal single-cell data. *Cell.* 2021;184(13):3573-87.e29.
10. Aran D, Looney AP, Liu L, Wu E, Fong V, Hsu A, et al. Reference-based analysis of lung single-cell sequencing reveals a transitional profibrotic macrophage. *Nature Immunology.* 2019;20(2):163-72.
11. Jin S, Guerrero-Juarez CF, Zhang L, Chang I, Ramos R, Kuan C-H, et al. Inference and analysis of cell-cell communication using CellChat. *Nature Communications.* 2021;12(1):1088.
12. Law CW, Chen Y, Shi W, and Smyth GK. voom: precision weights unlock linear model analysis tools for RNA-seq read counts. *Genome Biology.* 2014;15(2):R29.
13. Wickham H, and Wickham H. *Data analysis.* Springer; 2016.
14. Schneider CA, Rasband WS, and Eliceiri KW. NIH Image to ImageJ: 25 years of image analysis. *Nature Methods.* 2012;9(7):671-5.
15. Uhlén M, Fagerberg L, Hallström BM, Lindskog C, Oksvold P, Mardinoglu A, et al. Tissue-based map of the human proteome. *Science.* 2015;347(6220):1260419.
