# Supplementary figures and images for: Targeting immune checkpoint LAIR1 with antibody blockade or 3-in-1 CAR T cells enhances antitumor response
Source: J Clin Invest. 2025 Jul 1;135(16):e184043. doi: 10.1172/JCI184043 (PMC12352909; doi:10.1172/JCI184043)

FXIII-A

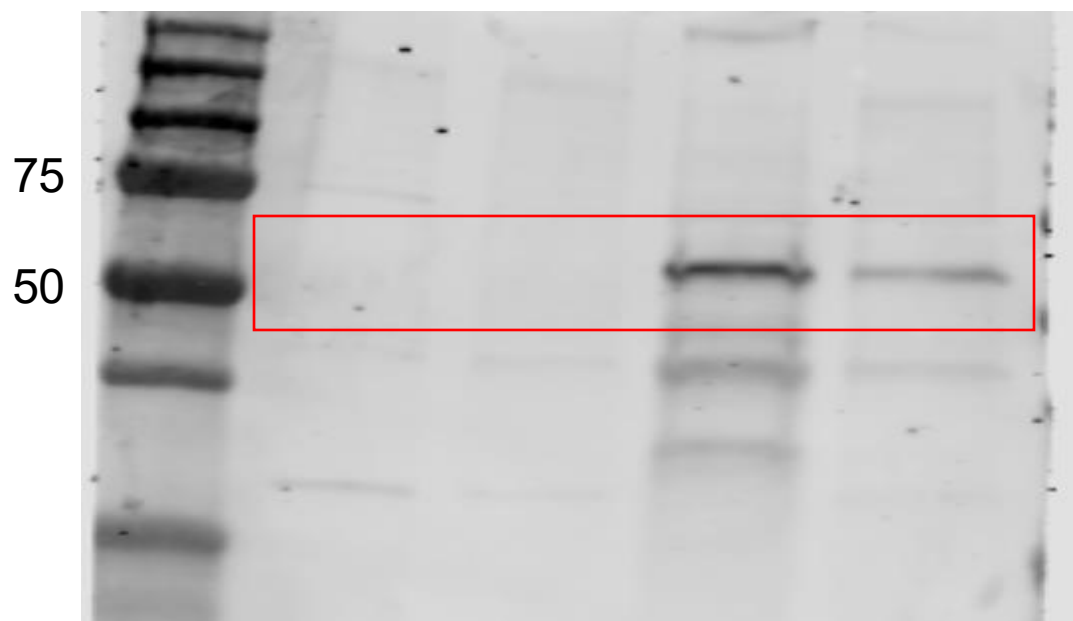

Actin

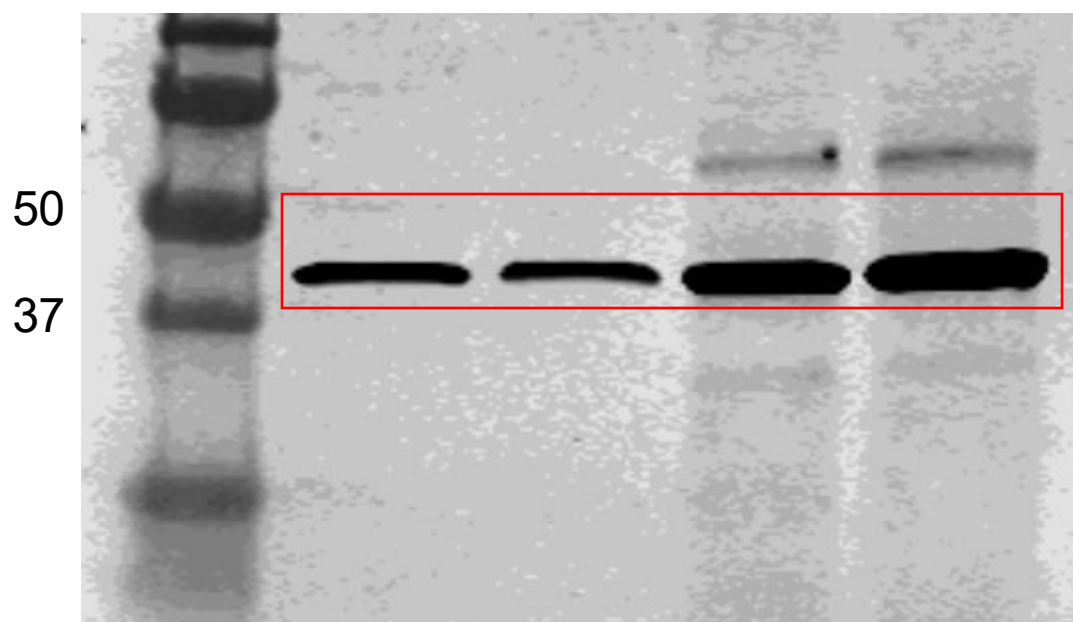

Original western blots images in **Figure 6K**.

Supplement: Unedited blot and gel images [file jci-135-184043-s008.pdf]
